# The synthesis of α-aryl-α-aminophosphonates and α-aryl-α-aminophosphine oxides by the microwave-assisted Pudovik reaction

**DOI:** 10.3762/bjoc.13.10

**Published:** 2017-01-12

**Authors:** Erika Bálint, Ádám Tajti, Anna Ádám, István Csontos, Konstantin Karaghiosoff, Mátyás Czugler, Péter Ábrányi-Balogh, György Keglevich

**Affiliations:** 1 MTΑ-BME Research Group for Organic Chemical Technology, 1521 Budapest, Hungary; 2 Department of Organic Chemistry and Technology, Budapest University of Technology and Economics, 1521 Budapest, Hungary; 3 Department Chemie, Ludwig-Maximilians-Universität München, 81377 München, Germany; 4 Hungarian Academy of Sciences, Research Centre for Natural Sciences, Institute of Organic Chemistry, 1519 Budapest, Hungary

**Keywords:** α-aryl-α-aminophosphine oxides, α-aryl-α-aminophosphonates, microwave, Pudovik reaction

## Abstract

A family of α-aryl-α-aminophosphonates and α-aryl-α-aminophosphine oxides was synthesized by the microwave-assisted solvent-free addition of dialkyl phosphites and diphenylphosphine oxide, respectively, to imines formed from benzaldehyde derivatives and primary amines. After optimization, the reactivity was mapped, and the fine mechanism was evaluated by DFT calculations. Two α-aminophosphonates were subjected to an X-ray study revealing a racemic dimer formation made through a N–H···O=P intermolecular hydrogen bridges pair.

## Introduction

α-Aminophosphonates and related derivatives, considered as the structural analogues of α-amino acids, have significant importance, especially in medicinal [1–3] and agricultural chemistry [4–5], due to their potential biological activity. The two major synthetic routes towards α-aminophosphonate derivatives embrace the Kabachnik–Fields (phospha-Mannich) three-component condensation, where an amine, an aldehyde or ketone and a >P(O)H reagent, such as a dialkyl phosphite or a secondary phosphine oxide react in a one-pot manner [6–10], and the Pudovik (aza-Pudovik) reaction, in which a >P(O)H species is added on the double bond of imines [11–14]. In this article, the latter pathway is utilized for the synthesis of α-aryl-α-aminophosphonates and α-aryl-α-aminophosphine oxides. In most cases, the additions were carried out in the presence of a catalyst and solvent. The use of many types of catalysts, such as acids (HCOOH) [15] or bases (NaOH [16], 1,8-diazabicyclo[5.4.0]undec-7-ene (DBU) [17], tetramethylguanidine (TMG) [18–19]), *p*-toluenesulfonyl chloride [20], metal salts (MgSO_4_) [21], CdI_2_ [22]), metal complexes (BF_3_·EtO_2_ [23–24], *t*-PcAlCl [15]), and phase-transfer catalysts [25] were reported. The enantioselective hydrophosphonylation of imines was also described, where chiral catalysts were applied in various solvents [26–32]. There are a few examples, where the reactions were performed in a solvent in the absence of any catalyst [33–38], while in a few cases the catalysts, i.e., PTSA [39], Na [40], TEA [21] or MoO_2_Cl_2_ [41], were used under solvent-free conditions. As for the microwave (MW)-assisted additions, only two cases were reported, but the catalytic variations were carried out in kitchen MW ovens [32,42], and thus, do lack of exact temperatures, these results cannot be reproduced. From a ’green chemical’ point of view, the solvent-free and catalyst-free additions are of interest, however, in these reactions, relatively long reaction times (1.5–10 h), and/or unreasonably large excesses (50–150 equiv) of the dialkyl phosphite were applied [43–52].

The synthesis of α-aryl-α-aminophosphine oxides by the addition of secondary phosphine oxides to imines is much less studied. Only a few publications were found and the reported reactions were performed in solvent (in DEE, THF or toluene) [53–56], or in the presence of a chiral catalyst [57]. There is only one solvent and catalyst-free example [58], but in this case a long reaction time (9 h) was required. In the Pudovik synthesis of α-aminophosphine oxides, the MW-assisted accomplishment has not been utilized at all. In the current paper, we wished to develop a facile catalyst and solvent-free MW-assisted method for the synthesis of α-aryl-α-aminophosphonates and α-aryl-α-aminophosphine oxides by the addition of dialkyl phosphites or diphenylphosphine oxide to the double bond of imines, and aimed at the preparation of new derivatives.

## Results and Discussion

### Synthesis of α-aryl-α-aminophosphonates and α-aminophosphine oxides

**Scheme 1:**
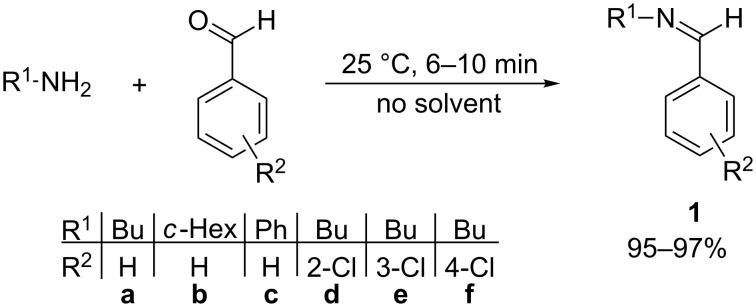
Synthesis of starting *N*-benzylideneamines **1**.

At first, the imine starting materials **1** were prepared by the condensation of benzaldehyde and its chloro-substituted derivatives with primary amines, such as butyl-, cyclohexylamine or aniline at room temperature under solvent-free conditions (Scheme 1).

Then, the reaction of *N*-benzylidene(butyl)amine (**1a**) with four different dialkyl phosphites and diphenylphosphine oxide was investigated under MW-assisted solvent-free conditions searching for the optimum temperature and reaction time (Table 1). The products were α-aminophosphonates **2a–d** and α-aminophosphine oxide **2e**.

**Table 1:** Addition of dialkyl phosphites and diphenylphosphine oxide to *N*-benzylidenebutylamine (**1a**).

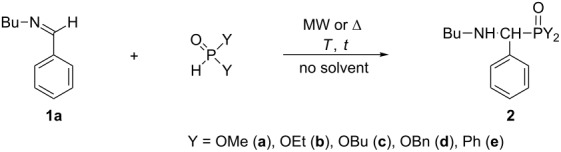								

Entry	Mode of heating	>P(O)H	>P(O)H (equiv)	*T* (°C)	*t* (min)	Composition (%)^a^		Yield^b^ (%)
						**1a**	**2**	

1	MW	DMP	1	80	30	5	95 (**2a**)	73
2	Δ	DMP	1	80	30	21	79 (**2a**)	–
3	MW	DMP	1	100	30	4	90^c^ (**2a**)	–
4	MW	DMP	1.2	100	30	0	94^c^ (**2a**)	–
5	MW	DEP	1	80	60	24	76 (**2b**)	–
6	MW	DEP	1	100	30	5	95 (**2b**)	–
7	MW	DEP	1.2	100	30	0	100 (**2b**)	85
8	Δ	DEP	1.2	100	30	17	83 (**2b**)	–
9	MW	DBuP	1.2	100	30	1	99 (**2c**)	90
10	MW	DBnP	1.2	100	30	0	100^d^ (**2d**)	69
11^e^	MW	DPPO	1.2	100	10	0	100^d^ (**2e**)	89

^a^On the basis of GC. ^b^After column chromatography. ^c^In these experiments byproduct **3** was also formed in a proportion of 6% based on GC–MS: 
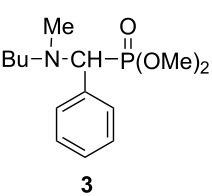
 ^d^On the basis of HPLC. ^e^Under N_2_ atmosphere.

The addition of 1 equivalent of dimethyl phosphite (DMP) to imine **1a** at 80 °C was almost complete after 30 min, and dimethyl ((butylamino)(phenyl)methyl)phosphonate (**2a**) could be isolated in a yield of 73% (Table 1, entry 1). Under similar conditions, the comparative thermal experiment led to a lower conversion of 79% (Table 1, entry 2). A full conversion could be achieved by heating at 100 °C under MW for 30 min although 1.2 equivalent of DMP were required. The formation of 6% of the *N*-methylated by-product **3** {δ_p_ (CDCl_3_) 25.4, [M + H]^+^_found_ = 286.1582, C_14_H_25_NO_3_P requires 286.1567} was inevitable (Table 1, entries 3 and 4). Similar *N*-methylations have been observed also in other cases [59–60]. At 80 °C, diethyl phosphite (DEP) was less reactive than DMP, as after an irradiation time of 1 h, the conversion was only 76% (Table 1, entry 5). At 100 °C/30 min, the reaction proceeded better (Table 1, entry 6), but a complete conversion was experienced only when using 1.2 equivalents of DEP (Table 1, entry 7). In the latter case, aminophosphonate **2b** was obtained in a yield of 85%. The comparative thermal experiment led again to a significantly lower conversion (Table 1, entry 8). Changing for dibutyl phosphite (DBuP), and applying it in a 1.2-fold quantity at 100 °C for 30 min, the addition was practically quantitative, and aminophosphonate **2c** was obtained in a yield of 90% (Table 1, entry 9). Under similar conditions, the reaction of dibenzyl phosphite (DBnP) was also clear-cut and afforded dibenzyl ((butylamino)(phenyl)methyl)phosphonate (**2d**) in 69% yield (Table 1, entry 10). Finally, diphenylphosphine oxide (DPPO) was added to imine **1a**. After 10 min irradiation at 100 °C, complete conversion was observed and aminophosphine oxide **2e** was obtained in a yield of 89% (Table 1, entry 11).

Next *N*-benzylidene(cyclohexyl)amine (**1b**) was tried in the aza-Pudovik reaction (Table 2).

Its reaction with 1.2 equivalents of DMP at 80 °C for 30 min led to aminophosphonate **4a** at a conversion of 89% (Table 2, entry 1). Even at 100 °C, no quantitative conversion could be achieved (Table 2, entry 2). There was need for 1.5 equivalents of DMP to attain a conversion of 99%. In this case, dimethyl ((cyclohexylamino)(phenyl)methyl)phosphonate (**4a**) was isolated in a yield of 87% (Table 2, entry 3). The comparative thermal experiment led to a somewhat lower conversion (Table 2, entry 4). The addition of DEP and DBuP at 100 °C on the C=N moiety of imine **1b** was quantitative or almost quantitative, respectively. The corresponding aminophosphonates **4b** and **4c** could be prepared in yields of approximately 92% (Table 2, entries 5 and 6). The reaction of *N*-benzylidene(cyclohexyl)amine (**1b**) with DBnP at 100 °C afforded the target compound **4d** in a conversion of 100% that could be isolated in a yield of only 68% (Table 2, entry 7). The phosphinoylation of imine **1b** was carried out at 100 °C for 10 min. The yield of ((cyclohexylamino)(phenyl)methyl)diphenylphosphine oxide (**4e**) was 88% (Table 2, entry 8). It can be noted that the reactivity of *N*-benzylidene(butyl)amine (**1a**) and *N*-benzylidene(cyclohexyl)amine (**1b**) is comparable.

**Table 2:** Addition of >P(O)H reagents to *N*-benzylidene(cyclohexyl)amine (**1b**).

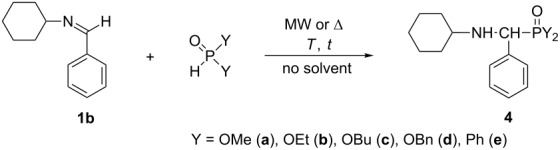								

Entry	Mode of heating	>P(O)H	>P(O)H (equiv)	*T* (°C)	*t* (min)	Composition (%)^a^		Yield^b^ (%)
						**1b**	**4**	

1	MW	DMP	1.2	80	30	11	89 (**4a**)	–
2	MW	DMP	1.2	100	30	7	93^c^ (**4a**)	–
3	MW	DMP	1.5	100	30	1	99 (**4a**)	87
4	Δ	DMP	1.5	100	30	9	91 (**4a**)	–
5	MW	DEP	1.2	100	30	0	100 (**4b**)	91
6	MW	DBuP	1.2	100	30	4	96^c^ (**4c**)	93
7	MW	DBnP	1.2	100	30	0	100^d^ (**4d**)	68
8^e^	MW	DPPO	1.2	100	10	0	100^d^ (**4e**)	88

^a^On the basis of GC. ^b^After column chromatography. ^c^There was no change for further irradiation. ^d^On the basis of HPLC. ^e^Under N_2_ atmosphere.

*N*-Benzylideneaniline (**1c**) revealed a somewhat enhanced reactivity in the reaction with the >P(O)H species (Table 3). The electron-donating butyl and cyclohexyl groups decrease the partial positive charge on the carbon atom of the >C=N- unit, as compared to the phenyl substituent. Hence, complete additions could be accomplished already at 80 °C within reaction times of 10–30 min. The yields of aminophosphonates **5a–c** were in the range of 92–97%, while the benzyl ester (**5d**) was obtained in a yield of 70% (Table 3, entries 1–4). The aminophosphine oxide **5e** was isolated in an 89% yield (Table 3, entry 5).

**Table 3:** MW-assisted addition of >P(O)H reagents to *N*-benzylideneaniline (**1c**).

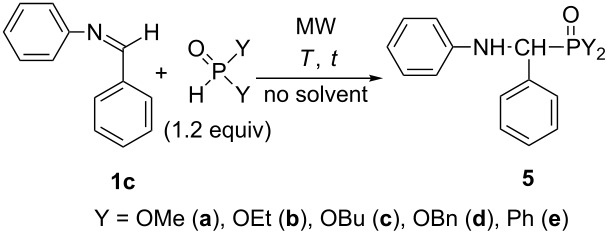						

Entry	>P(O)H	*T* (°C)	*t* (min)	Composition (%)^a^		Yield^b^ (%)
				**1c**	**5**	

1	OMe	80	10	1	99 (**5a**)	92
2	OEt	80	10	0	100 (**5b**)	93
3	OBu	80	20	0	100 (**5c**)	97
4	OBn	80	30	0	100^c^ (**5d**)	70
5^d^	Ph	80	10	0	100^c^ (**5e**)	89

^a^On the basis of GC. ^b^After column chromatography. ^c^On the basis of HPLC. ^d^Under N_2_ atmosphere.

Next, *N*-chlorobenzylidene(butyl)amines **1d–f** were reacted with dialkyl phosphites at 80–100 °C to obtain the corresponding α-aminophosphonates **6a–c**, **7a–c** and **8a–c**. The results are collected in Table 4. The dialkyl ((butylamino)(chlorophenyl)methyl)phosphonates (**6–8**, **a–c**) were prepared in yields of 72–94% (Table 4, entries 1–9). There were no observable differences in the reactivities, as compared to the unsubstituted model compound **1a**.

**Table 4:** MW-assisted addition of dialkyl phosphites to *N*-chlorobenzylidene(butyl)amines **1d**–**f**.

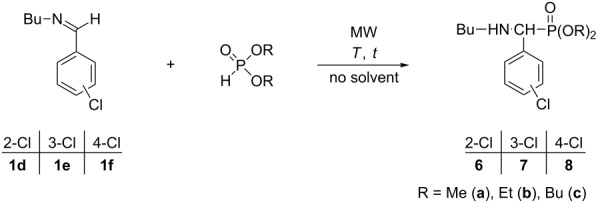								

Entry	Imine	>P(O)H	>P(O)H (equiv)	*T* (°C)	*t* (min)	Composition (%)^a^		Yield^b^ (%)
						**1**	Product	

1	**1d**	DMP	1	80	30	1	99 (**6a**)	91
2	**1d**	DEP	1.2	100	20	0	100(**6b**)	72
3	**1d**	DBuP	1.2	100	30	0	100 (**6c**)	74
4	**1e**	DMP	1	80	30	12	88 (**7a**)	80
5	**1e**	DEP	1.2	100	20	3	97 (**7b**)	94
6	**1e**	DBuP	1.2	100	30	7	93 (**7c**)	73
7	**1f**	DMP	1	80	30	12	88 (**8a**)	75
8	**1f**	DEP	1.2	100	20	0	100 (**8b**)	80
9	**1f**	DBuP	1.2	100	30	2	98 (**8c**)	81

^a^On the basis of GC. ^b^After column chromatography.

All together 24 compounds including 21 α-aryl-α-aminophosphonates and three α-aryl-α-aminophosphine oxides were obtained by column chromatography and characterized. Among the aminophosphonates, 16 are new, and five are known in the literature, two out of the three aminophosphine oxides are new compounds.

### Study on the addition of diethyl phosphite to *N*-benzylidene(butyl)amine by in situ FTIR spectroscopy

**Scheme 2:**
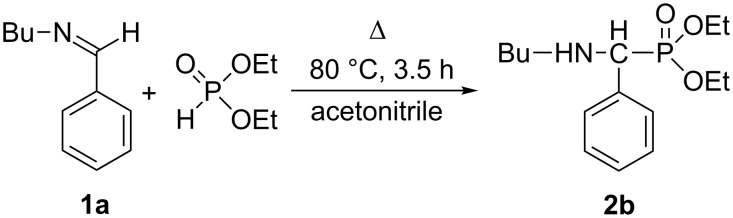
Addition of diethyl phosphite to *N*-benzylidene(butyl)amine in acetonitrile.

**Figure 1:**
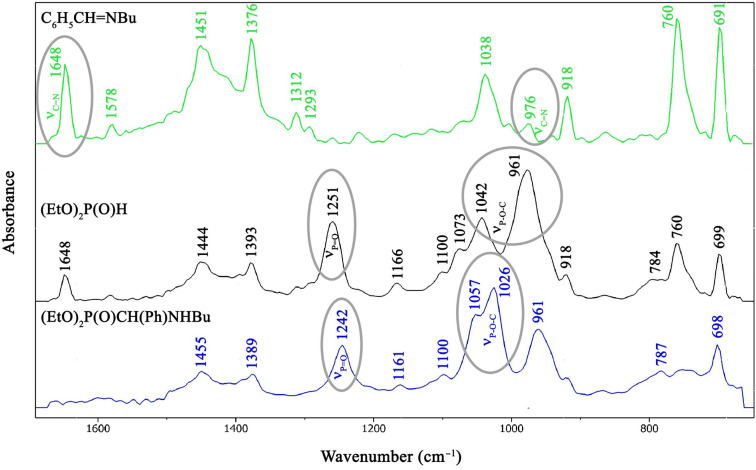
IR spectra of the reaction components in acetonitrile solution.

Then, we wished to follow the addition reaction of DEP to *N*-benzylidene(butyl)amine (**1a**) at 80 °C in acetonitrile (Scheme 2) by in situ Fourier transform IR spectroscopy. Initially the IR spectra of the reaction components were recorded: imine **1a**, DEP and diethyl ((butylamino)(phenyl)methyl)phosphonate (**2b**) (Table 5 and Figure 1). The spectrum of imine **1a** has a strong absorption band at 1648 cm^−1^ corresponding to ν_C=N_. At the same time, DEP may be identified by the strong signals at 961, 1042 and 1251 cm^−1^ assigned to the ν_P–O–C_ and the ν_P=O_ vibrations, respectively. The similar ν_P–O–C_ and ν_P=O_ absorptions of the product α-aminophosphonate **2b** were somewhat shifted to 1026, 1057 and 1242 cm^−1^, respectively.

**Table 5:** Characteristic IR absorptions of the reaction components.

C_6_H_5_CH=NBu (**1a**)		(EtO)_2_P(O)H		(EtO)_2_P(O)CH(Ph)NHBu (**2b**)	

976 cm^−1^	ν_C=N_	961 cm^−1^	ν_P–O–C_	1026 cm^−1^	ν_P–O–C_
1648 cm^−1^	ν_C=N_	1042 cm^−1^	ν_P–O–C_	1057 cm^−1^	ν_P–O–C_
		1251 cm^−1^	ν_P=O_	1242 cm^−1^	ν_P=O_

**Figure 2:**
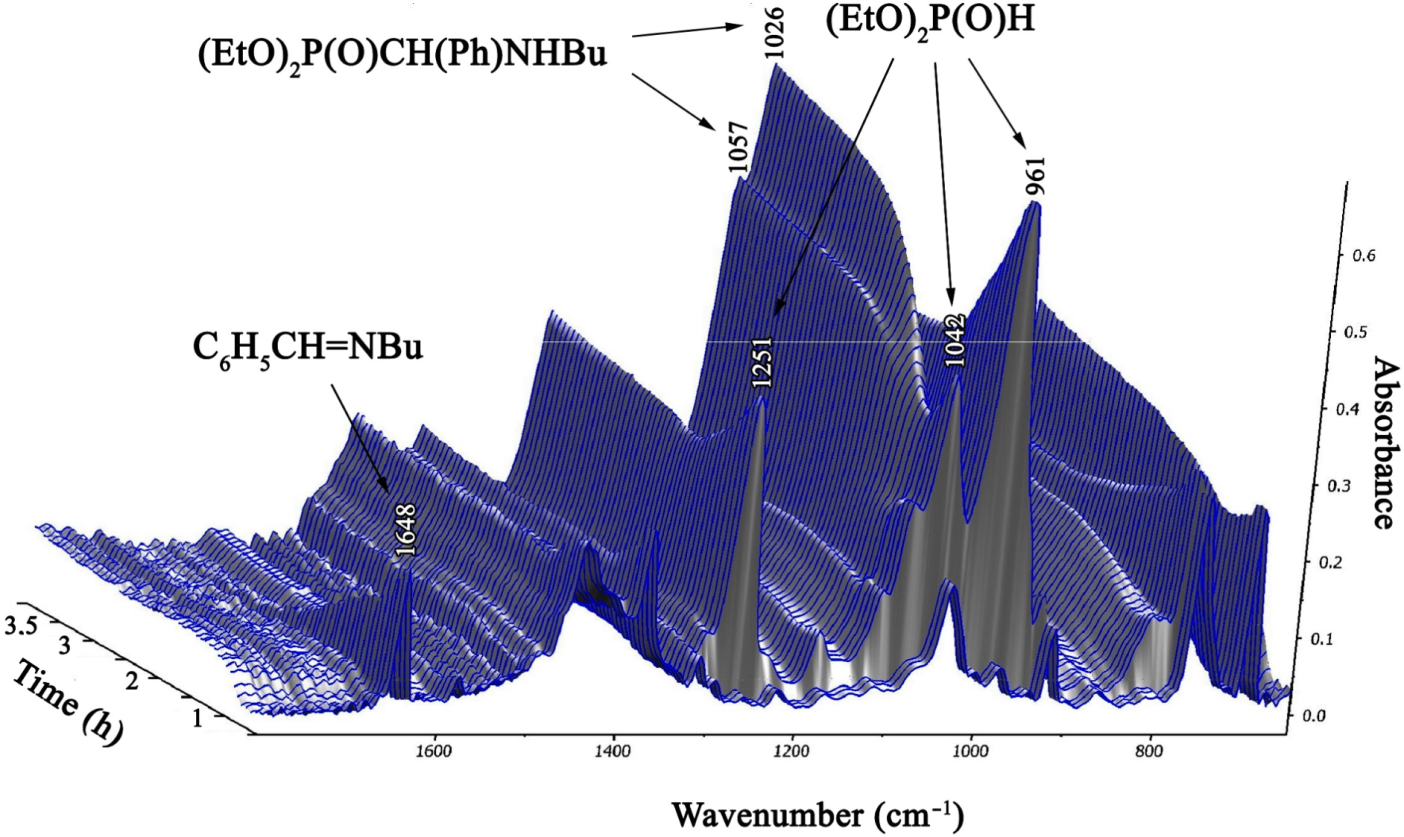
A segment of the time-dependent IR spectrum for the addition of diethyl phosphite to *N*-benzylidene(butyl)amine (**1a**) in acetonitrile under formation of diethyl ((butylamino)(phenyl)methyl)phosphonate (**2b**).

A segment of the time-dependent IR spectrum (3D diagram) can be seen in Figure 2.

**Figure 3:**
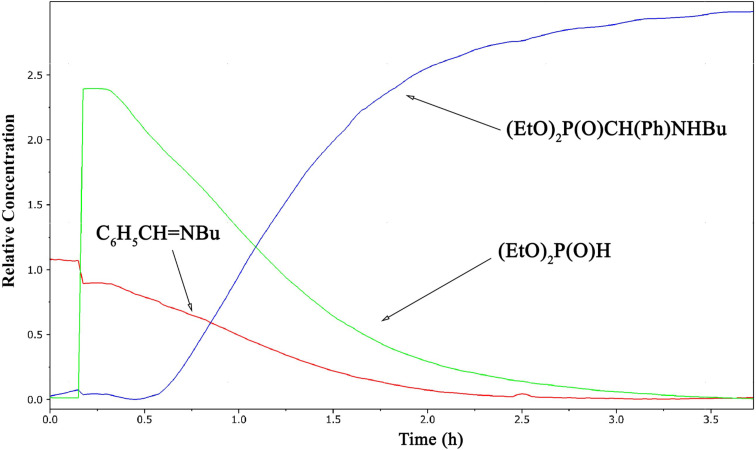
Concentration profiles of the reaction components in the addition reaction at 80 °C in acetonitrile.

The obtained results from this study are shown in Figure 3 and show the concentration profile of the starting components imine **1a** and DEP and the product **2b**. The diagram was constructed by a so-called deconvolution on the basis of the decrease/increase of the different absorptions on the reaction time scale. This calculation (MCR-ALS, multivariate curve resolution – alternating least squares) gives the concentration profiles of the components and also the spectra of pure components. From Figure 3, it can be seen that the addition reaction was complete after 3.5 h.

Finally, the characteristic IR absorptions were taken from the calculated IR spectra and compared with those obtained in acetonitrile solutions (Table 6). As can be seen, the agreement is rather good.

**Table 6:** IR absorptions measured and obtained from the 3D diagram after deconvolution (in cm^−1^).

C_6_H_5_CH=NBu (**1a**)		(EtO)_2_P(O)H		(EtO)_2_P(O)CH(Ph)NHBu (**2b**)	
Measured	Obtained	Measured	Obtained	Measured	Obtained

1648	1648	1251	1262	1242	1246
976	976	1073	1073	1057	1053
		1042	1046	1026	1026
		961	980	961	961

### An X-ray crystallographic study on the α-aminophosphonates **5b** and **5d**

**Figure 4:**
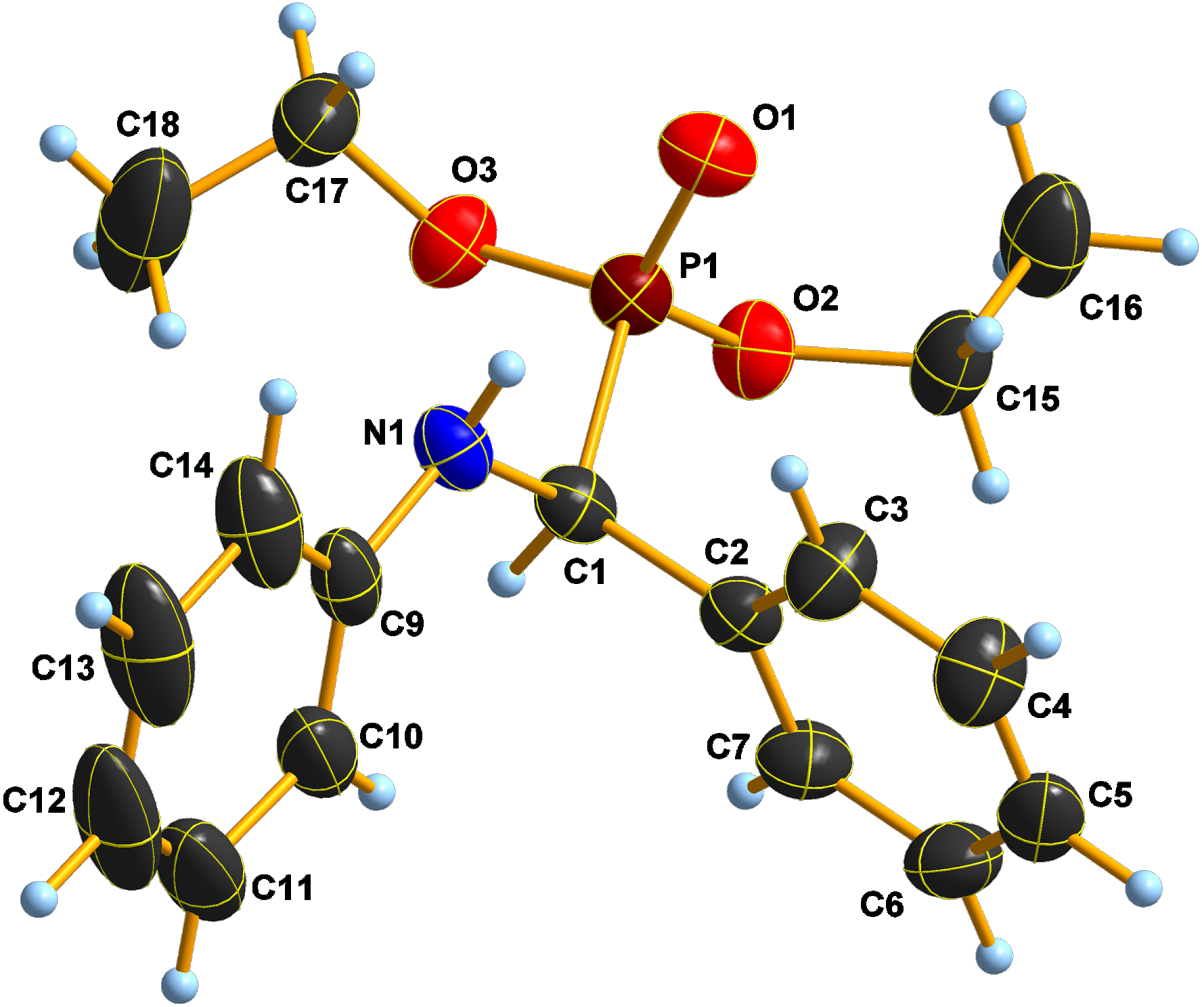
Atomic numbering with anisotropic displacements plot of **5b** at −100 °C.

Large crystals of **5b** are composed of very thin plates and one of these plates was dissected and used for X-ray measurement at low temperatures. There seems to be a slight disorder, which becomes evident in the ellipsoids of the aniline ring and one of the methyl carbon atoms (Figure 4).

The crystal structure of compound **5b** was published almost 40 years ago [61], and a subsequent NMR and theoretical study was also done for comparative purposes [62]. This latter work claims *SS* and *RR* dimers, which is obviously wrong, as the centrosymmetric dimers formed must be *RS* due to symmetry. The crystal structure of **5b** does not undergo a phase transition at −100 ºC, hence, it must be similar to the sample studied in the earlier work at room temperature [62]. Apart from the formation of the centrosymmetric dimer through a strong N–H···O=P hydrogen bridge (cf. Table 7), there is a C–H^…^O short contact to an ester oxygen atom of a next, translated and inverted **5b** molecule, as well. In this way, sheets of symmetry center related dimers fused into endless sheets are formed in the crystal (cf. Supporting Information File 1, Figure S1).

**Figure 5:**
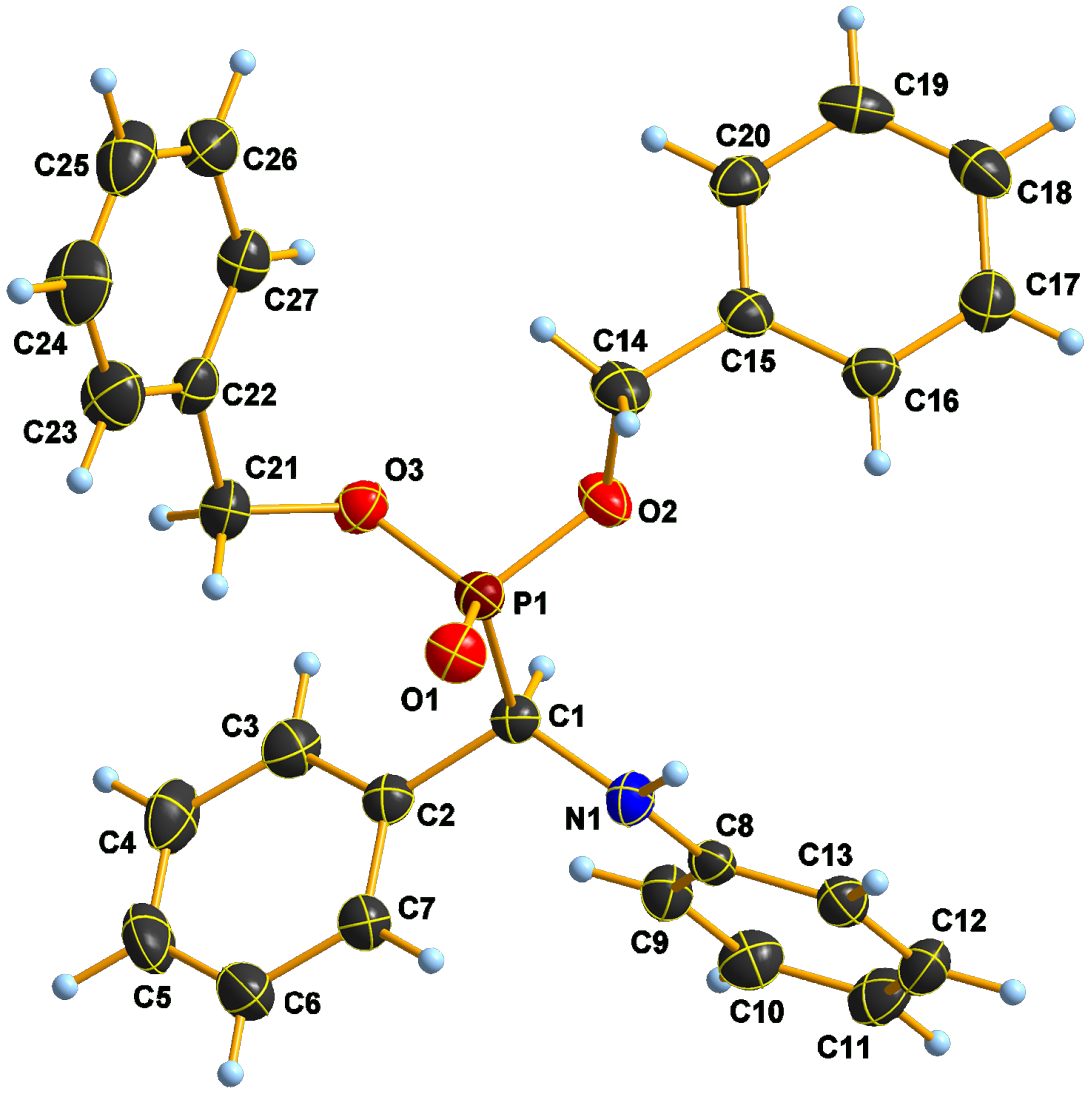
Atomic numbering with anisotropic displacements plot of **5d** at −100 °C.

In the crystal structure of **5d** (Figure 5), the formation of hydrogen-bonded dimers occurs through the same N–H···O=P hydrogen bonds in the solid state (Table 7 and Supporting Information File 1, Figure S2), too.

**Table 7:** Hydrogen bond dimensions (Å, Deg) for compounds **5b** and **5d**.^a^

**5b**				

D–H···A	D–H (Å)	H···A (Å)	D···A (Å)	D–H···A (°)
N1–H1A···O1^b^	0.88(4)	2.09(4)	2.957(3)	167(4)
C7–H7···O3^c^	0.94(3)	2.48(3)	3.367(3)	159(1)

**5d**				

N1–H1A···O1^d^	0.87(2)	2.20(2)	3.051(2)	166(2)

^a^D, donor; A, acceptor; ^b^symmetry related operator = 1−x,−y,−z; ^c^symmetry related operator = 1−x, 1−y,−z; ^d^symmetry related operator = 1−x,−y,2−z.

A short intramolecular H(1)···H(10) distance in **5b** appears to be nearly by −0.3 Å shorter than the sum of vdW radii (2.11(2) << 2.40), and is somewhat similar to those observed in other aminophosphonates [63]. No such short distance is seen in compound **5d**, where all H···H contacts appear around or longer than the respective sums of van der Waals radii, and the only short attractive contact is in the H-bridge promoted dimer.

### Theoretical study on the addition of the >P(O)H species to *N*-benzylideneamines

The calculations were carried out by the B3LYP/6-31G (d,p) method. In the solvent-free reaction, the dielectric constant of the P reagent was estimated as 78.3553. This value was taken into account as an implicit solvent effect. First, the tautomeric equilibria of dialkyl phosphites and diphenylphosphine oxide were studied (Table 8). It was not surprising to find that the pentavalent tetracoordinated form **A** was by 25–28 kJ mol^−1^ more stable than the trivalent form **B**.

**Table 8:** Relative enthalpies, free energies and entropies for the tautomeric forms in case of methoxy and phenyl substituents.

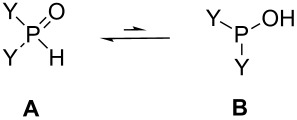							

Y = MeO	Δ*H* [kJ mol^−1^]	Δ*G* [kJ mol^−1^]	Δ*S* [J (mol K)^−1^]	Y = Ph	Δ*H* [kJ mol^−1^]	Δ*G* [kJ mol^−1^]	Δ*S* [J (mol K)^−1^]

**A**	0	0	0	**A**	0	0	0
**B**	27.7	26.4	1.1	**B**	24.7	22.3	1.9

The calculations predicted the attack of the trivalent form **B** of the P reagent on the nitrogen atom of the C=N unit of the imine **1**. Moreover, the P–C and the N–H bonds are formed in a single concerted step, via five-membered transition states **TS 2**, **4**, and **5** (Table 9) and the additions are in all cases exothermic. The reactions with dimethyl phosphite are more favorable than those with diphenylphosphine oxide. It can also be seen that the additions to *N*-benzylideneaniline have the greatest driving force of ca. 31 kJ mol^−1^. The energy content of the TSs fall in the range of 77–89 kJ mol^−1^.

**Table 9:** Relative enthalpies, free energies and entropies for the addition reaction of >P(O)H species to imines.

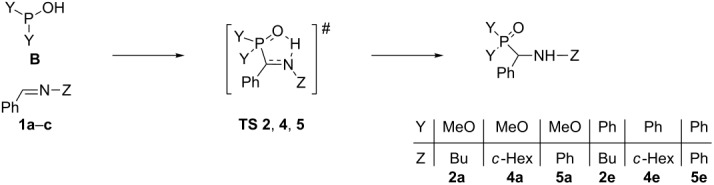							

Y = MeO	Δ*H*^0^/Δ*H*^#^ [kJ mol^−1^]	Δ*G*^0^/Δ*G*^#^ [kJ mol^−1^]	Δ*S*^0^/Δ*S*^#^ [J (mol K)^−1^]	Y = Ph	Δ*H*^0^/*Δ*H^#^ [kJ mol^−1^]	Δ*G*^0^/Δ*G*^#^ [kJ mol^−1^]	Δ*S*^0^/Δ*S*^#^ [J (mol K)^−1^]

Z = Bu				Z = Bu			
**B** + **1a** → **2a**	−21.1	−4.7	−13.1	**B** + **1a** → **2e**	−10.9	3.5	−11.5
**TS 2a**	83.9	100.5	−13.3	**TS 2e**	80.8	92.4	−9.3

Z = *c*-hex				Z = *c*-hex			
**B** + **1b** → **4a**	−16.6	−5.6	−8.8	**B** + **1b** → **4e**	−7.6	8.3	−12.8
**TS 4a**	89.2	96.7	−6.0	**TS 4e**	85.3	100.8	−12.4

Z = Ph				Z = Ph			
**B** + **1c** → **5a**	−31.3	−17.4	−11.2	**B** + **1c** → **5e**	−30.9	−15.3	−12.5
**TS 5a**	89.0	98.8	−7.9	**TS 5e**	77.4	90.9	−10.8

**Figure 6:**
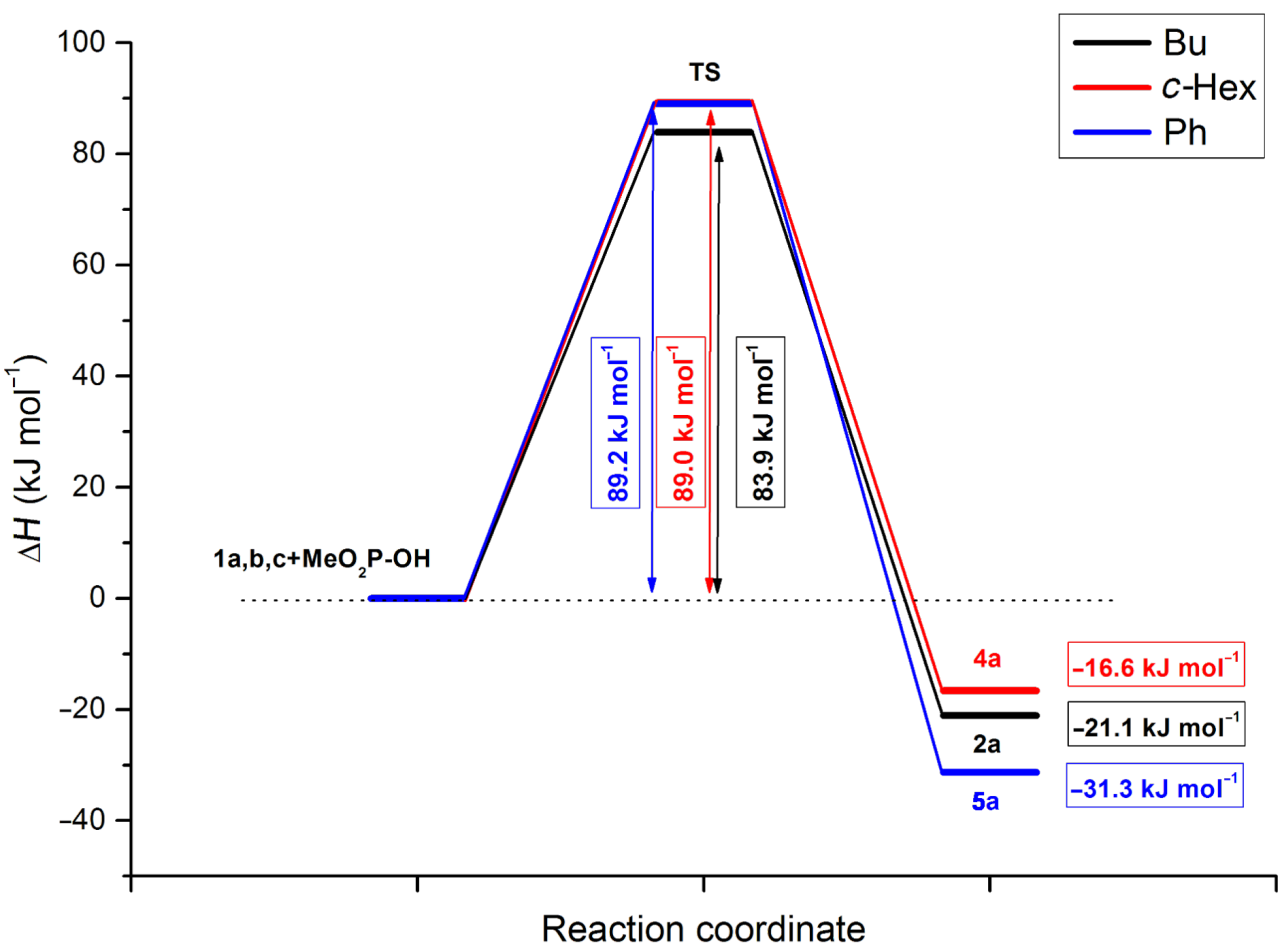
The energy diagram for the reaction with dimethyl phosphite.

**Figure 7:**
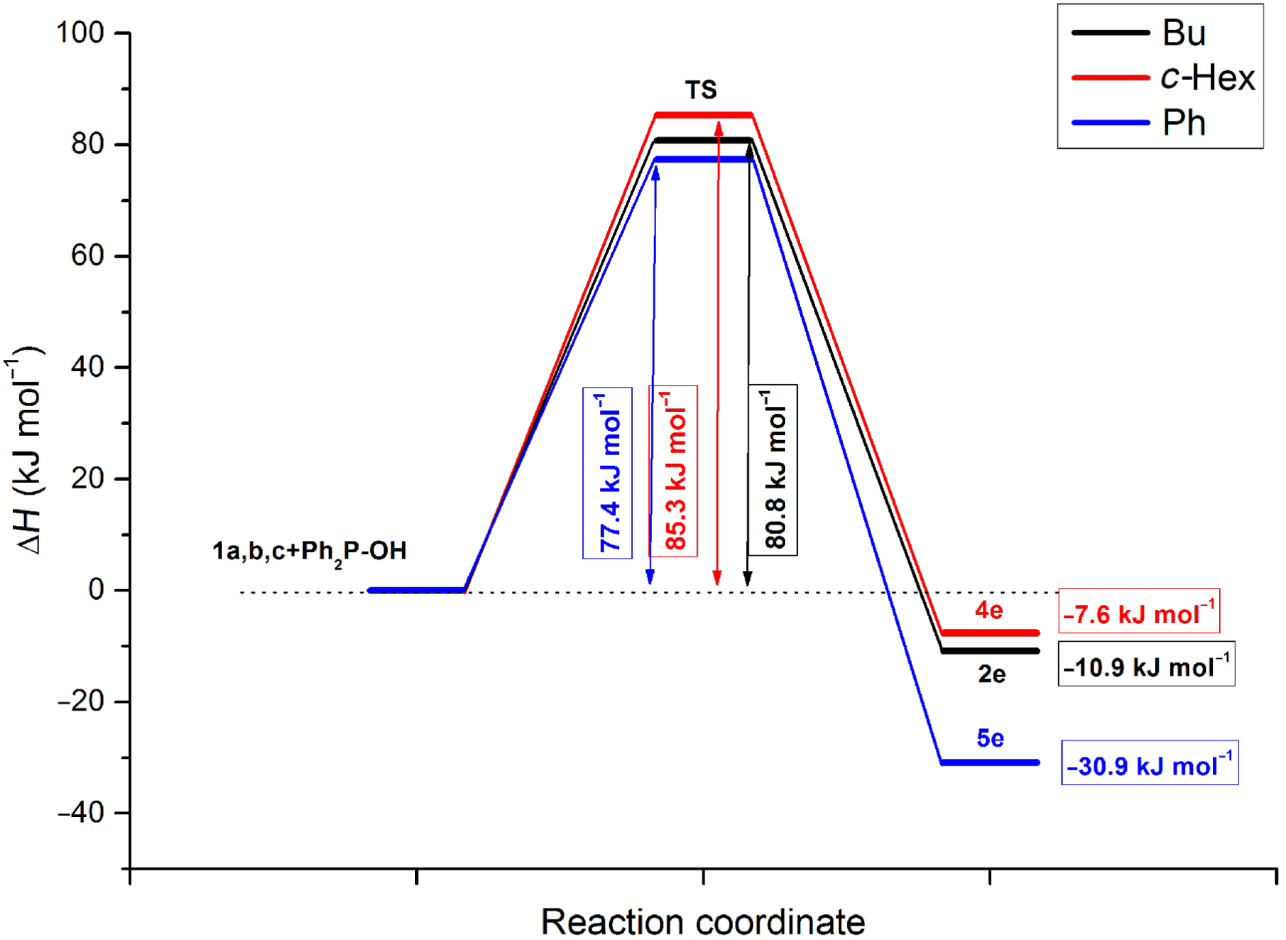
The energy diagram for the reaction with diphenylphosphine oxide.

The energy diagrams for the reactions with dimethyl phosphite and diphenylphosphine oxide are shown in Figure 6 and Figure 7, respectively.

## Conclusion

In conclusion, we have developed an efficient, solvent-free and catalyst-free MW-assisted method for the Pudovik synthesis of α-aryl-α-aminophosphonates and α-aryl-α-aminophosphine oxides. This method is a novel approach for the preparation of the target compounds, and was optimized for each case. Twenty-four derivatives were isolated and characterized. Except six compounds, these were new compounds. Furthermore, the reactivity was mapped, and the mechanism was evaluated by B3LYP/6-31G (d,p) calculations. The crystal structure of two α-aminophosphonates was studied by X-ray analysis suggesting centrosymmetric dimers.

## Supporting Information

File 1 Experimental, X-ray crystallographic data, and NMR spectra.
