# Supplementary material for: The synthesis of α-aryl-α-aminophosphonates and α-aryl-α-aminophosphine oxides by the microwave-assisted Pudovik reaction
Source: Beilstein J Org Chem. 2017 Jan 12;13:76–86. doi: 10.3762/bjoc.13.10 (PMC5238548; doi:10.3762/bjoc.13.10)
Supplement: File 1 — Experimental, X-ray crystallographic data, and NMR spectra. [file Beilstein_J_Org_Chem-13-76-s001.pdf]

Supporting Information
for
The synthesis of α-aryl-α-aminophosphonates and α-aryl-α-aminophosphine oxides by the microwave-assisted Pudovik reaction

Erika Bálint[1*], Ádám Tajti[2], Anna Ádám[2], István Csontos[2], Konstantin Karaghiosoff[3], Mátyás Czugler[2], Péter Ábrányi-Balogh[4] and György Keglevich[2*]

Address: [1]MTA-BME Research Group for Organic Chemical Technology, 1521 Budapest, Hungary, [2]Department of Organic Chemistry and Technology, Budapest University of Technology and Economics, 1521 Budapest, Hungary, [3]Department Chemie, Ludwig-Maximilians-Universität München, 81377 München, Germany and [4]Hungarian Academy of Sciences, Research Centre for Natural Sciences, Institute of Organic Chemistry, 1519 Budapest, Hungary

Email: Erika Bálint* - ebalint@mail.bme.hu; György Keglevich* - gkeglevich@mail.bme.hu
*Corresponding author

Experimental, X-ray crystallographic data, and NMR spectra

Table of contents

General information S2
General procedure for the preparation of *N*-benzylideneamines S3
General procedure for the synthesis of the α-aminophosphonates and α-aminophosphine oxides........ S4
Real time monitoring of the addition of diethyl phosphite to *N*-benzylidene(butyl)amine S12
Single crystal X-ray diffraction measurements........ S13
Quantum chemical calculations S16
^{31}P NMR, ^{1}H NMR and ^{13}C NMR spectra........ S17
References S51

General information

The reactions were carried out in a 300 W CEM Discover focused microwave reactor (CEM Microwave Technology Ltd., Buckingham, UK) equipped with a pressure controller using 20–30 W irradiation under isothermal conditions.

GC measurements were performed on an HP5890 series 2 GC-FID chromatograph, using a 15 m × 0.18 mm Restek, Rtx-5 column with a film layer of 0.20 μm. The temperature of the column was initially held at 40 °C for 1 min, followed by programming at 25 °C/min up to 300 °C, and a final period at 300 °C (isothermal) for 10 min. The temperature of the injector was 290 °C, and of the FID detector 300 °C. The carrier gas was N_2.

GC–MS measurements were performed on an Agilent 6890 N-GC-5973 N-MSD chromatograph, using a 30 m × 0.25 mm Restek, Rtx-5SILMS column with a film layer of 0.25 μm. The initial temperature of column was 45 °C for 1 min, followed by programming at 10 °C/min up to 310 °C and a final period at 310 °C (isothermal) for 17 min. The temperature of the injector was 250 °C. The carrier gas was He and the operation mode was splitless.

LC–MS experiments were performed with an Agilent 1200 liquid chromatography system coupled with a 6130 quadrupole mass spectrometer equipped with an ESI ion source (Agilent Technologies, Palo Alto, CA, USA). Analysis was performed at 40 °C on a Gemini C18 column (150 mm × 4.6 mm, 3 μm; Phenomenex, Torrance, CA, USA) with a mobile phase flow rate of 0.6 mL/min. The composition of eluent A was 0.1% NH_4HCO_3 in water; eluent B was 0.1% NH_4HCO_3 and 8% water in acetonitrile; 0→3 min 5% B, 3→13 min gradient, 13→20 min 95% B. The injection volume was 20 μL. The chromatographic profile was registered at 222 nm. The MSD operating parameters were as follows: positive ionization mode, scan spectra from *m*/*z* 100 to 1000, drying gas temperature 300 °C, nitrogen-flow rate 12 L/min, nebulizer pressure 60 psi, capilary voltage 2500 V.

High resolution mass spectrometric measurements were performed using a Q-TOF Premier mass spectrometer in positive electrospray mode.

The ^{31}P, ^{13}C, ^{1}H NMR spectra were taken in $CDCl_3$ solution on a Bruker AV-300 or DRX-500 spectrometer operating at 121.5, 75.5 and 300 or 202.4, 125.7 and 500 MHz, respectively. Chemical shifts are downfield relative to 85% H_3PO_4 and TMS. Non-equivalence effects were observed in ^{1}H and $^{13}C\{^{1}H\}$ NMR spectra. Corresponding pairs of resonances were marked with (I) and (II), respectively.

In situ FTIR measurements were conducted using a ReactIR 1000 equipment (Mettler-Toledo Inc., Columbus, OH). An attenuated total reflectance (ATR) diamond probe was placed in a

100-mL four-necked flask equipped with a dropping funnel, a condenser, a thermometer, and a magnetic stirrer. The temperature was maintained by using an appropriately adjusted oil bath.

General procedure for the preparation of *N*-benzylideneamines

A mixture of 25 mmol of amine [butylamine (2.5 mL), cyclohexylamine (2.9 mL), aniline (2.3 mL)] and 25 mmol of aldehyde [benzaldehyde (2.6 mL), 2-chlorobenzaldehyde (2.8 mL), 3-chlorobenzaldehyde (2.8 mL), 4-chlorobenzaldehyde (3.5 g)] was stirred for 6–10 min at rt (25 °C). After completion, 10 mL of dichloromethane were added to the mixture, dried (Na_2SO_4) and filtered. Evaporation of the volatile components provided the products **1a–f**.

***N*-Benzylidene(butyl)amine (1a)**

Yield: 97% (3.9 g) of compound **1a** as pale yellow oil; ^{13}C NMR ($CDCl_3$) δ 13.9 (CH_3), 20.5 (CH_3*C*H_2), 33.0 (*C*H_2CH_2N), 61.5 (CH_2N), 128.0 (C_2), 128.6 (C_3), 130.4 (C_4), 136.4 (C_1), 160.7 (CH=N); δ [S1] ($CDCl_3$) 13.5, 20.1, 32.6, 61.1, 127.6, 128.1, 130.0, 136.0, 160.2; $[M+H]^+_{found}$ = 162.1282, $C_{11}H_{16}N$ requires 162.1277.

***N*-Benzylidene(cyclohexyl)amine (1b)**

Yield: 96% (4.5 g) of compound **1b** as yellow oil; ^{13}C NMR ($CDCl_3$) δ 24.8 (C_3), 25.7 (C_4), 34.4 (C_2), 70.0 (C_1), 128.1 ($C_{3'}$), 128.5 ($C_{2'}$), 130.3 ($C_{4'}$), 136.6 ($C_{1'}$), 158.6 (CH=N); δ [S1] ($CDCl_3$) 24.8, 25.6, 34.4, 70.0, 128.0, 128.5, 130.3, 136.6, 158.5; $[M+H]^+_{found}$ = 188.1441, $C_{13}H_{18}N$ requires 188.1434.

***N*-Benzylideneaniline (1c)**

Yield: 96% (4.3 g) of compound **1c** as yellow crystal; Mp: 47-48 °C; Mp [S2]: 50-51 °C; ^{13}C NMR ($CDCl_3$) δ 120.9 (C_2), 125.9 (C_4), 128.77 ($C_{3'}$), 128.81 (C_3), 129.1 ($C_{2'}$), 131.4 ($C_{4'}$), 136.2 ($C_{1'}$), 152.1 (C_1), 160.4 (CH=N); δ [S1] ($CDCl_3$) 120.9, 125.9, 128.79, 128.83, 129.2, 131.4, 136.3, 152.1, 160.4; $[M+H]^+_{found}$ = 182.0972, $C_{13}H_{12}N$ requires 182.0964.

***N*-2-Chlorobenzylidene(butyl)amine (1d)**

Yield: 95% (4.6 g) of compound **1d** as pale yellow oil; ^{13}C NMR ($CDCl_3$) δ 13.9 (CH_3), 20.5 (CH_3*C*H_2), 32.9 (*C*H_2CH_2N), 61.6 (CH_2N), 127.0 (C_5), 128.3 (C_6), 129.7 (C_3), 131.3 (C_4), 133.4 (C_2), 134.9 (C_1), 157.5 (CH=N); δ [S3] (DMSO) 13.85, 20.42, 32.92, 61.56, 126.92, 128.26, 129.68, 131.21, 133.40, 134.88, 157.44; $[M+H]^+_{found}$ = 196.0895, $C_{11}H_{15}NCl$ requires 196.0888.

N-3-Chlorobenzylidene(butyl)amine (1e)

Yield: 97% (4.7 g) of compound **1e** as yellow oil; ^{13}C NMR ($CDCl_3$) δ 13.9 (CH_3), 20.5 (CH_3*C*H_2), 32.9 (*C*H_2CH_2N), 61.4 (CH_2N), 126.3 (C_6), 127.7 (C_2), 129.8 (C_5), 130.4 (C_4), 134.8 (C_1), 138.2 (C_3), 159.2 (CH=N); ^{1}H NMR ($CDCl_3$) δ 0.95 (t, J_{HH} = 7.3, 3H, CH_3), 1.31-1.46 (m, 2H, CH_3C*H_2*), 1.61-1.75 (m, 2H, *C*H_2CH_2N), 3.62 (t, J_{HH} = 7.0, 2H, CH_2N), 7.29-7.41 (m, 2H, C_4H, C_5H), 7.53-7.59 (m, 1H, C_6H), 7.75 (s, 1H, C_2H), 8.22 (s, 1H, CH=N); $[M+H]^+_{found}$ = 196.0892, $C_{11}H_{15}NCl$ requires 196.0888.

N-4-chlorobenzylidene(butyl)amine (1f)

Yield: 96% (4.7 g) of compound **1f** as yellow oil; ^{13}C NMR ($CDCl_3$) δ 13.9 (CH_3), 20.5 (CH_3*C*H_2), 33.0 (*C*H_2CH_2N), 61.4 (CH_2N), 128.8 (C_3), 129.2 (C_2), 134.9 (C_1), 136.3 (C_4), 159.3 (CH=N); δ [S4] ($CDCl_3$) 13.9, 20.4, 32.9, 61.4, 128.8, 129.2, 134.9, 136.3, 159.3; $[M+H]^+_{found}$ = 196.0887, $C_{11}H_{15}NCl$ requires 196.0888.

General procedure for the synthesis of the α-aminophosphonates and α-aminophosphine oxides

A mixture of 1 mmol imine derivate [**1a** (0.16 g), **1b** (0.19 g), **1c** (0.18 g), **1d-e** (0.20 g)] and >P(O)H derivative (1.0 mmol [dimethyl phosphite (0.092 mL), diethyl phosphite (0.13 mL)] or 1.2 mmol [dimethyl phosphite (0.11 mL), diethyl phosphite (0.15 mL), dibutyl phosphite (0.23 mL), dibenzyl phosphite (0.27 mL), diphenylphosphine oxide (0.24 g)] or 1.5 mmol [dimethyl phosphite (0.14 mL)]) was irradiated in a sealed tube at 80–100 °C for 10–60 min in a CEM Microwave reactor equipped with a pressure controller. The volatile components were removed under reduced pressure. The residue obtained was purified by flash column chromatography using silica gel and dichloromethane/methanol 97:3 or ethyl acetate/hexane 1:1 as the eluent to afford α-aminophosphonates and α-aminophosphine oxides.

Dimethyl ((butylamino)(phenyl)methyl)phosphonate (2a)

Yield: 73% (0.20 g) of compound **2a** as colorless oil; ^{31}P NMR ($CDCl_3$) δ 26.0; ^{13}C NMR ($CDCl_3$) 13.9 (*C*H_3CH_2), 20.3 (CH_3*C*H_2), 32.0 (d, J_{CP} = 0.5, *C*H_2CH_2N), 47.7 (d, $^3J_{CP}$ = 16.7, CH_2N), 53.5 (d, $^2J_{CP}$ = 7.0, CH_3O^I), 53.7 (d, $^2J_{CP}$ = 7.1, CH_3O^{II}), 60.8 (d, $^1J_{CP}$ = 153.4, CHP), 127.9 (d, J_{CP} = 3.2, C_4), 128.4 (d, $^3J_{CP}$ = 6.2, C_2), 128.5 (d, J_{CP} = 2.5, C_3), 136.0 (d, $^2J_{CP}$ = 3.8, C_1); ^{1}H NMR ($CDCl_3$) δ 0.86 (t, J_{HH} = 7.2, 3H, C*H_3*CH_2), 1.21-1.38 (m, 2H, CH_3C*H_2*), 1.38-1.50 (m, 2H, C*H_2*CH_2N), 1.94 (s, 1H, NH), 2.36-2.59 (m, 2H, CH_2N), 3.55 (d, J_{HP} = 10.4, 3H,

CH_3O^I), 3.74 (d, J_{HP} = 10.5, 3H, CH_3O^{II}), 4.05 (d, J_{HP} = 20.1, 1H, CHP), 7.26-7.45 (m, 5H, ArH); $[M+H]^+_{found}$ = 272.1408, $C_{13}H_{23}NO_3P$ requires 272.1410.

Diethyl ((butylamino)(phenyl)methyl)phosphonate (2b)

Yield: 85% (0.25 g) of compound **2b** as colorless oil; ^{31}P NMR ($CDCl_3$) δ 23.8; δ [S5] ($CDCl_3$) 23.6; $[M+H]^+_{found}$ = 300.1734, $C_{15}H_{27}NO_3P$ requires 300.1723.

Dibutyl ((butylamino)(phenyl)methyl)phosphonate (2c)

Yield: 90% (0.32 g) of compound **2c** as colorless oil; ^{31}P NMR ($CDCl_3$) δ 24.2; ^{13}C NMR ($CDCl_3$) 13.6 ($\mathit{C}H_3(CH_2)_3O^I$), 13.7 ($\mathit{C}H_3(CH_2)_3O^{II}$), 14.0 ($\mathit{C}H_3(CH_2)_3N$), 18.6 ($\mathit{C}H_2(CH_2)_2O^I$), 18.7 ($\mathit{C}H_2(CH_2)_2O^{II}$), 20.3 ($\mathit{C}H_2(CH_2)_2N$), 32.0 (d, J_{CP} = 0.5, $\mathit{C}H_2CH_2N$), 32.5 (d, $^3J_{CP}$ = 5.8, $\mathit{C}H_2CH_2O^I$), 32.6 (d, $^3J_{CP}$ = 5.8, $\mathit{C}H_2CH_2O^{II}$), 47.8 (d, $^3J_{CP}$ = 16.5, CH_2N), 61.2 (d, $^1J_{CP}$ = 152.7, CHP), 66.4 (d, $^2J_{CP}$ = 7.2, CH_2O^I), 66.6 (d, $^2J_{CP}$ = 7.3, CH_2O^{II}), 127.7 (d, J_{CP} = 3.2, C_4), 128.3 (d, J_{CP} = 2.5, C_3), 128.5 (d, $^3J_{CP}$ = 6.2, C_2), 136.5 (d, $^2J_{CP}$ = 4.1, C_1); ^1H NMR ($CDCl_3$) δ 0.84 (t, J_{HH} = 7.2, 3H, $C\mathit{H}_3(CH_2)_3O^I$), 0.86 (t, J_{HH} = 6.8, 3H, $C\mathit{H}_3(CH_2)_3N$), 0.90 (t, J_{HH} = 7.2, 3H, $C\mathit{H}_3(CH_2)_3O^{II}$), 1.16-1.53 (m, 10H, $C\mathit{H}_2C\mathit{H}_2CH_2N$, $C\mathit{H}_2CH_2CH_2O$, $C\mathit{H}_2CH_2O^I$), 1.54-1.67 (m, 2H, $C\mathit{H}_2CH_2O^{II}$), 1.98 (s, 1H, NH), 2.37-2.60 (m, 2H, CH_2N) 3.69-3.82 (m, 1H, CH_A, CH_2O^I), 3.84-3.95 (m, 1H, CH_B, CH_2O^I), 3.95-4.09 [4.02 (d, J_{HP} = 19.6, CHP) overlapped by the multiplet of CH_2O^{II} total int. 3H], 7.23-7.37 (m, 3H, C_2H, C_4H), 7.37-7.46 (m, 2H, C_3H); $[M+H]^+_{found}$ = 356.2360, $C_{19}H_{35}NO_3P$ requires 356.2349.

Dibenzyl ((butylamino)(phenyl)methyl)phosphonate (2d)

Yield: 69% (0.29 g) of compound **2d** as colorless oil; ^{31}P NMR ($CDCl_3$) δ 25.3; ^{13}C NMR ($CDCl_3$) 13.9 (CH_3), 20.2 ($CH_3\mathit{C}H_2$), 32.0 (d, J_{CP} = 0.4, $\mathit{C}H_2CH_2N$), 47.8 (d, $^3J_{CP}$ = 16.7, CH_2N), 61.4 (d, $^1J_{CP}$ = 152.6, CHP), 68.1 (d, $^2J_{CP}$ = 6.8, CH_2O^I), 68.3 (d, $^2J_{CP}$ = 7.1, CH_2O^{II}), 127.7 ($C_{2'}{}^I$), 127.85 (d, J_{CP} = 3.1, C_4), 127.88 ($C_{2'}{}^{II}$), 128.15 ($C_{4'}{}^I$), 128.22 ($C_{4'}{}^{II}$), 128.37 ($C_{3'}{}^I$), 128.440 (d, J_{CP} = 2.5, C_3), 128.444 ($C_{3'}{}^{II}$), 125.5 (d, $^3J_{CP}$ = 6.3, C_2), 136.0 (d, $^2J_{CP}$ = 3.7, C_1), 136.3 (d, $^3J_{CP}$ = 6.2, $C_{1'}{}^I$), 136.5 (d, $^3J_{CP}$ = 6.2, $C_{1'}{}^{II}$); ^1H NMR ($CDCl_3$) δ 0.84 (t, J_{HH} = 7.2, 3H, $C\mathit{H}_3$), 1.17-1.33 (m, 2H, $CH_3C\mathit{H}_2$), 1.33-1.45 (m, 2H, $C\mathit{H}_2CH_2N$), 1.84 (s, 1H, NH), 2.34-2.58 (m, 2H, CH_2N), 4.06 (d, J_{HP} = 20.0, 1H, CHP), 4.73 (dd, J_{HP} = 11.9, J_{HH} = 8.1, 1H, CH_A, CH_2O^I), 4.88 (dd, J_{HP} = 11.9, J_{HH} = 7.2, 1H, CH_B, CH_2O^I), 5.02 (qd, J_{HP} = 11.9, J_{HH} = 8.0, 2H, CH_2O^{II}), 7.08-7.19 (m, 2H, $C_{4'}H$), 7.21-7.46 (m, 13H, $C_{2\text{-}4}H$, $C_{2'}H$ and $C_{3'}H$), $[M+H]^+_{found}$ = 424.2037, $C_{25}H_{31}NO_3P$ requires 424.2036.

((Butylamino)(phenyl)methyl)diphenylphosphine oxide (2e)

Yield: 89% (0.32 g) of compound **2e** as white crystal; Mp: 166-167 °C; ^{31}P NMR ($CDCl_3$) δ 31.1; ^{13}C NMR ($CDCl_3$) δ 13.9 (CH_3), 20.2 (CH_3*C*H_2), 31.9 (*C*H_2CH_2N), 47.7 (d, $^3J_{CP}$ = 13.8, CH_2N), 63.5 (d, $^1J_{CP}$ = 79.4, CHP), 127.6 (d, J_{CP} = 2.7, C_4), 128.0 (d, $^2J_{CP}$ = 11.5, $C_{2'}^{I}$), 128.1 (d, J_{CP} = 2.1, C_3), 128.3 (d, $^2J_{CP}$ = 11.4, $C_{2'}^{II}$), 128.8 (d, $^3J_{CP}$ = 5.2, C_2), 131.2 (d, $^1J_{CP}$ = 95.6, $C_{1'}^{I}$), 131.3 (d, $^1J_{CP}$ = 97.0, $C_{1'}^{II}$), 131.5 (d, J_{CP} = 2.8, $C_{4'}^{I}$), 131.7 (d, $^3J_{CP}$ = 8.7, $C_{3'}^{I}$), 131.8 (d, J_{CP} = 3.0, $C_{4'}^{II}$), 132.1 (d, $^3J_{CP}$ = 8.6, $C_{3'}^{II}$), 135.6 (d, $^2J_{CP}$ = 1.8, C_1); ^{1}H NMR ($CDCl_3$) δ 0.82 (t, J_{HH} = 7.2, 3H, CH_3), 1.16-1.31 (m, 2H, CH_3CH_2), 1.32-1.46 (m, 2H, CH_2CH_2N), 1.76 (s, 1H, NH), 2.34-2.48 (m, 1H, CH_A, CH_2N), 2.50-2.64 (m, 1H, CH_B, CH_2N), 4.45 (d, J_{HP} = 11.3, 1H, CHP), 7.09-7.22 (m, 5H, $C_{2-4}H$), 7.27-7.62 (m, 8H, $C_{2'}H$, $C_{4'}H$, $C_{3'}H^{I}$), 7.80-7.92 (m, 2H, $C_{3'}H^{II}$); $[M+H]^+_{found}$ = 364.1830, $C_{23}H_{27}NOP$ requires 364.1825.

Dimethyl ((cyclohexylamino)(phenyl)methyl)phosphonate (4a)

Yield: 87% (0.26 g) of compound **4a** as colorless oil; ^{31}P NMR ($CDCl_3$) δ 26.5; δ [S6] ($CDCl_3$) 26.5; $[M+H]^+_{found}$ = 298.1559, $C_{15}H_{25}NO_3P$ requires 298.1567.

Diethyl ((cyclohexylamino)(phenyl)methyl)phosphonate (4b)

Yield: 91% (0.30 g) of compound **4b** as colorless; ^{31}P NMR ($CDCl_3$) δ 24.1; δ [S5] ($CDCl_3$) 24.3; $[M+H]^+_{found}$ = 326.1890, $C_{17}H_{29}NO_3P$ requires 326.1880.

Dibutyl ((cyclohexylamino)(phenyl)methyl)phosphonate (4c)

Yield: 93% (0.35 g) of compound **4c** as colorless oil; ^{31}P NMR ($CDCl_3$) δ 24.2; ^{13}C NMR ($CDCl_3$) 13.6 (CH_3^{I}), 13.7 (CH_3^{II}), 18.6 ($CH_3CH_2^{I}$), 18.7 ($CH_3CH_2^{II}$), 24.4 (C_3^{I}), 24.9 (C_3^{II}), 26.1 (C_4), 32.0 (C_2^{I}), 32.4 (d, $^3J_{CP}$ = 5.9, $CH_2CH_2O^{I}$), 32.6 (d, $^3J_{CP}$ = 5.9, $CH_2CH_2O^{II}$), 34.4 (C_2^{II}), 53.5 (d, $^3J_{CP}$ = 15.5, C_1), 57.6 (d, $^1J_{CP}$ = 153.5, CHP), 66.3 (d, $^2J_{CP}$ = 7.3, CH_2O^{I}), 66.8 (d, $^2J_{CP}$ = 7.2, CH_2O^{II}), 127.6 (d, J_{CP} = 3.2, $C_{4'}$), 128.3 (d, J_{CP} = 2.5, $C_{3'}$), 128.5 (d, $^3J_{CP}$ = 6.2, $C_{2'}$), 137.0 (d, $^2J_{CP}$ = 2.8, $C_{1'}$); ^{1}H NMR ($CDCl_3$) δ 0.82 (t, J_{HH} = 7.3, 3H, CH_3^{I}), 0.91 (t, J_{HH} = 7.3, 3H, CH_3^{II}), 0.99-1.29 (m, 7H, $C_{2-4}H_{eq}$, $CH_3CH_2^{I}$), 1.30-1.47 (m, 4H, $CH_3CH_2^{II}$, $CH_2CH_2O^{I}$), 1.48-1.73 (m, 6H, C_2H_{ax}, C_3H_{ax}, $CH_2CH_2O^{II}$), 1.88 (bs, 2H, NH, C_4H_{ax}), 2.28-2.43 (m, 1H, C_1H), 3.63-3.76 (m, 1H, CH_A, CH_2O^{I}), 3.80-3.93 (m, 1H, CH_B, CH_2O^{I}), 3.96-4.14 (m, 2H, CH_2O^{II}), 4.19 (d, J_{HP} = 22.0, CHP), 7.23-7.36 (m, 3H, $C_{2'}H$, $C_{4'}H$), 7.37-7.43 (m, 2H, $C_{3'}H$); $[M+H]^+_{found}$ = 382.2501, $C_{21}H_{37}NO_3P$ requires 382.2506.

Dibenzyl ((cyclohexylamino)(phenyl)methyl)phosphonate (4d)

Yield: 68% (0.31 g) of compound **4d** as colorless oil; ^{31}P NMR ($CDCl_3$) δ 25.0; ^{13}C NMR ($CDCl_3$) 24.4 (C_3^{I}), 24.8 (C_3^{II}), 26.0 (C_4), 32.0 (C_2^{I}), 34.3 (C_2^{II}), 53.6 (d, $^3J_{CP}$ = 15.0, C_1), 57.9 (d, $^1J_{CP}$ = 153.3, CHP), 67.9 (d, $^2J_{CP}$ = 7.0, CH_2O^{I}), 68.6 (d, $^2J_{CP}$ = 7.0, CH_2O^{II}), 127.7 ($C_{2''}^{I}$), 127.8 (d, J_{CP} = 3.2, $C_{4'}$), 127.9 ($C_{2''}^{II}$), 128.1 ($C_{4''}^{I}$), 128.2 ($C_{4''}^{II}$), 128.4 ($C_{3''}^{I}$), 128.44 ($C_{3''}^{II}$), 128.45 (d, J_{CP} = 2.4, $C_{3'}$), 128.50 (d, $^3J_{CP}$ = 6.3, $C_{2'}$), 136.4 (d, $^3J_{CP}$ = 6.2, $C_{1''}^{I}$), 136.6 (d, $^2J_{CP}$ = 2.5, $C_{1'}$), 136.7 (d, $^3J_{CP}$ = 6.1, $C_{1''}^{II}$); ^{1}H NMR ($CDCl_3$) δ 0.89-1.24 (m, 5H, $C_{2\text{-}4}H_{ax}$), 1.44-1.73 (m, 4H, C_2H_{ax}, C_3H_{ax}), 1.76-1.90 (m, 2H, NH, C_4H_{ax}), 2.35 (bs, 1H, C_1H) 4.26 (d, J_{HP} = 22.1, 1H, CHP), 4.68 (dd, J_{HP} = 11.7, J_{HH} = 8.1, 1H, CH_A, CH_2O^{I}), 4.86 (dd, J_{HP} = 11.8, J_{HH} = 7.1, 1H, CH_B, CH_2O^{I}), 5.07 (qd, J_{HP} = 11.9, J_{HH} = 7.7, 2H, CH_2O^{II}), 7.12 (bs, 2H, $C_{4''}$H), 7.21-7.44 (m, 13H, $C_{2'\text{-}4'}$H, $C_{2''}$H, $C_{3''}$H), $[M+H]^+_{found}$ = 450.2207, $C_{27}H_{33}NO_3P$ requires 450.2193.

((Cyclohexylamino)(phenyl)methyl)diphenylphosphine oxide (4e)

Yield: 88% (0.34 g) of compound **4e** as white crystal; Mp: 189-190 °C; ^{31}P NMR ($CDCl_3$) δ 31.5; ^{13}C NMR ($CDCl_3$) δ 24.2 (C_3^{I}), 24.7 (C_3^{II}), 26.0 (C_4), 31.7 (C_2^{I}), 34.4 (C_2^{II}), 53.6 (d, $^3J_{CP}$ = 12.8, C_1), 59.9 (d, $^1J_{CP}$ = 79.8, CHP), 127.4 (d, J_{CP} = 2.7, $C_{4'}$), 127.9 (d, $^2J_{CP}$ = 11.5, $C_{2''}^{I}$), 128.1 (d, J_{CP} = 2.1, $C_{3'}$), 128.3 (d, $^2J_{CP}$ = 11.4, $C_{2''}^{II}$), 128.8 (d, $^3J_{CP}$ = 5.1, $C_{2'}$), 131.41 (d, J_{CP} = 2.7, $C_{4''}^{I}$), 131.42 (d, $^1J_{CP}$ = 96.4, $C_{1''}^{I}$), 131.63 (d, $^3J_{CP}$ = 8.7, $C_{3''}^{I}$), 131.64 (d, $^1J_{CP}$ = 97.2, $C_{1''}^{II}$), 131.7 (d, J_{CP} = 2.8, $C_{4''}^{II}$), 132.1 (d, $^3J_{CP}$ = 8.6, $C_{3''}^{II}$), 136.0 (d, $^2J_{CP}$ = 1.6, $C_{1'}$); ^{1}H NMR ($CDCl_3$) δ 0.97-1.21 (m, 5H, $C_{2\text{-}4}H_{eq}$), 1.48-1.60 (m, 4H, C_2H_{ax} and C_3H_{ax}), 1.87 (bs, 1H, C_4H_{ax}), 2.04 (s, 1H, NH), 2.34 (bs, 1H, C_1H), 4.63 (d, J_{HP} = 11.6, 1H, CHP), 7.16 (bs, 5H, $C_{2'\text{-}4'}$H), 7.22-7.31 (m, 2H, $C_{3''}H^{I}$), 7.33-7.42 (m, 1H, $C_{4''}H^{I}$), 7.43-7.59 (m, 5H, $C_{2''}$H, $C_{4''}H^{II}$), 7.85-7.97 (m, 2H, $C_{3''}H^{II}$); $[M+H]^+_{found}$ = 390.1968, $C_{25}H_{29}NOP$ requires 390.1981.

Dimethyl ((phenylamino)(phenyl)methyl)phosphonate (5a)

Yield: 92% (0.27 g) of compound **5a** as yellow crystal; Mp: 94-95 °C; Mp [S7]: 90-92 °C; ^{31}P NMR ($CDCl_3$) δ 25.2; δ [S8] ($CDCl_3$) 25.3; $[M+H]^+_{found}$ = 292.1103, $C_{15}H_{19}NO_3P$ requires 292.1097.

Diethyl ((phenylamino)(phenyl)methyl)phosphonate (5b)

Yield: 93% (0.29 g) of compound **5b** as pale blue crystal; Mp: 91-92 °C; Mp [S9]: 90-91 °C; ^{31}P NMR ($CDCl_3$) δ 22.8; δ [S9] ($CDCl_3$) 23.2; $[M+H]^+_{found}$ = 320.1418, $C_{17}H_{23}NO_3P$ requires 320.1410.

Dibutyl ((phenylamino)(phenyl)methyl)phosphonate (5c)

Yield: 97% (0.36 g) of compound **5c** as greenish-blue oil; ^{31}P NMR ($CDCl_3$) δ 22.7; ^{13}C NMR ($CDCl_3$) 13.5 (CH_3^{I}), 13.6 (CH_3^{II}), 18.6 ($CH_3\mathit{C}H_2^{I}$), 18.7 ($CH_3\mathit{C}H_2^{II}$), 32.4 (d, $^3J_{CP}$ = 5.7, $\mathit{C}H_2CH_2O^{I}$), 32.6 (d, $^3J_{CP}$ = 5.7, $\mathit{C}H_2CH_2O^{II}$), 56.1 (d, $^1J_{CP}$ = 150.8, CHP), 67.0 (d, $^2J_{CP}$ = 7.4, CH_2O^{I}), 67.1 (d, $^2J_{CP}$ = 7.5, CH_2O^{II}), 113.9 (C_2), 118.4 (C_4), 127.90 (d, $^3J_{CP}$ = 5.6, $C_{2'}$), 127.92 (d, J_{CP} = 2.8, $C_{4'}$), 128.6 (d, J_{CP} = 2.7, $C_{3'}$), 129.2 (C_3), 136.0 (d, $^2J_{CP}$ = 2.7, $C_{1'}$), 146.4 (d, $^3J_{CP}$ = 14.5, C_1); δ [S10] ($CDCl_3$) 13.33, 13.37, 13.39, 18.35, 18.49, 18.55, 32.13, 32.2, 32.26, 32.33, 32.39, 55.13, 56.63, 65.28, 65.35, 66.65, 66.72, 66.74, 66.81, 113.66, 113.69, 118.16, 127.72, 128.37, 128.43, 135.86, 135.88, 146.14, 146.28; ^{1}H NMR ($CDCl_3$) δ 0.82 (t, J_{HH} = 7.3, 3H, CH_3^{I}), 0.88 (t, J_{HH} = 7.3, 3H, CH_3^{II}), 1.16-1.29 (m, 2H, $CH_3C\mathit{H}_2^{I}$), 1.29-1.49 (m, 4H, $CH_3C\mathit{H}_2^{II}$, $C\mathit{H}_2CH_2O^{I}$), 1.51-1.72 (m, 2H, $C\mathit{H}_2CH_2O^{II}$), 3.53-3.68 (m, 1H, CH_A, CH_2O^{I}), 3.81-3.94 (m, 1H, CH_B, CH_2O^{I}), 3.95-4.14 (m, 2H, CH_2O^{II}), 4.77 (d, J_{HP} = 24.2, CHP), 5.32 (bs, 1H, NH), 6.60 (d, J_{HH} = 7.8, 2H, C_2H), 6.68 (t, J_{HH} = 7.3, 1H, C_4H), 7.09 (t, J_{HH} = 7.9, 2H, C_3H), 7.20-7.38 (m, 3H, $C_{2'}$H, $C_{4'}$H), 7.42-7.51 (m, 2H, $C_{3'}$H); δ [S10] ($CDCl_3$) 0.82 (t, J = 7.4, 3H), 0.88 (t, J = 7.4, 3H), 1.17-1.27 (m, 8H), 3.54-4.14 (m, 5H), 4.73 (d, J = 24.2, 1H), 6.57-6.60 (m, 2H), 6.66-6.70 (m, 1H), 7.07-7.11 (m, 2H), 7.30-7.33 (m, 3H), 7.45-7.48 (m, 2H); $[M+H]^+_{found}$ = 376.2044, $C_{21}H_{31}NO_3P$ requires 376.2036.

Dibenzyl ((phenylamino)(phenyl)methyl)phosphonate (5d)

Yield: 70% (0.31 g) of compound **5d** as yellow crystal; Mp: 118-119 °C; ^{31}P NMR ($CDCl_3$) δ 23.6; ^{13}C NMR ($CDCl_3$) 56.3 (d, $^1J_{CP}$ = 150.4, CHP), 68.5 (d, $^2J_{CP}$ = 6.9, CH_2O^{I}), 68.6 (d, $^2J_{CP}$ = 7.0, CH_2O^{II}), 113.9 (C_2), 118.5 (C_4), 127.88 ($C_{2''}^{I}$), 127.93 (d, $^3J_{CP}$ = 5.6, $C_{2'}$), 127.98 (d, J_{CP} = 2.4, $C_{4'}$), 128.05 ($C_{2''}^{II}$), 128.4 ($C_{4''}^{I}$), 128.46 ($C_{4''}^{II}$), 128.49 ($C_{3''}^{I}$), 128.6 ($C_{3''}^{II}$), 128.7 (d, J_{CP} = 2.7, $C_{3'}$), 129.1 (C_3), 135.7 (d, $^2J_{CP}$ = 2.6, $C_{1'}$), 135.9 (d, $^3J_{CP}$ = 6.0, $C_{1''}^{I}$), 136.0 (d, $^3J_{CP}$ = 5.8, $C_{1''}^{II}$), 146.1 (d, $^3J_{CP}$ = 14.5, C_1); ^{1}H NMR ($CDCl_3$) δ 4.59 (dd, J_{HP} = 11.7, J_{HH} = 8.3, 1H, CH_A, CH_2O^{I}), 4.69 (bs, 1H, NH), 4.80 (d, J_{HP} = 24.5, 1H, CHP), 4.86 (dd, J_{HP} = 11.7, J_{HH} = 7.6, 1H, CH_B, CH_2O^{I}), 5.00 (qd, J_{HP} = 11.7, J_{HH} = 8.4, 2H, CH_2O^{II}), 6.53 (d, J_{HH} = 7.7, 2H, C_2H), 6.68 (t, J_{HH} = 7.3, 1H, C_4H), 7.03-7.16 [7.08 (t, J_{HH} = 7.6, 2H, C_2H) overlapped by the multiplet of $C_{4''}$H total int. 4H], 7.20-7.38 (m, 11H, $C_{2'}$H, $C_{4'}$H, $C_{2''}$H, $C_{3''}$H), 7.40-7.49 (m, 2H, $C_{3'}$H); $[M+H]^+_{found}$ = 444.1717, $C_{27}H_{27}NO_3P$ requires 444.1723.

((Phenylamino)(phenyl)methyl)diphenylphosphine oxide (5e)

Yield: 89% (0.34 g) of compound **5e** as yellow crystal; Mp: 252-253 °C; Mp [S11]: 252 °C; ^{31}P NMR ($CDCl_3$) δ 33.2; δ [S12] ($CDCl_3$) 33.6; $[M+H]^+_{found}$ = 384.1525, $C_{25}H_{23}NOP$ requires 384.1512.

Dimethyl ((butylamino)(2-chlorophenyl)methyl)phosphonate (6a)

Yield: 91% (0.28 g) of compound **6a** as colorless oil; ^{31}P NMR ($CDCl_3$) δ 25.5; ^{13}C NMR ($CDCl_3$) 13.9 ($\mathit{C}H_3CH_2$), 20.2 ($CH_3\mathit{C}H_2$), 31.9 (d, J_{CP} = 0.6, $\mathit{C}H_2CH_2N$), 47.5 (d, $^3J_{CP}$ = 16.4, CH_2N), 53.3 (d, $^2J_{CP}$ = 6.8, CH_3O^I), 53.9 (d, $^2J_{CP}$ = 6.8, CH_3O^{II}), 55.8 (d, $^1J_{CP}$ = 155.7, CHP), 127.2 (d, J_{CP} = 2.8, C_5), 128.9 (d, J_{CP} = 3.1, C_3), 129.3 (d, $^3J_{CP}$ = 4.3, C_6), 129.5 (d, J_{CP} = 2.2, C_4), 134.3 (d, $^2J_{CP}$ = 1.2, C_1), 134.6 (d, $^3J_{CP}$ = 9.0, C_2); ^{1}H NMR ($CDCl_3$) δ 0.86 (t, J_{HH} = 7.3, 3H, $C\mathit{H}_3CH_2$), 1.23-1.37 (m, 2H, $CH_3C\mathit{H}_2$), 1.37-1.49 (m, 2H, $C\mathit{H}_2CH_2N$), 1.69 (bs, 1H, NH), 2.46 (t, J_{HH} = 6.9, 2H, CH_2N), 3.54 (d, J_{HP} = 10.5, 3H, CH_3O^I), 3.84 (d, J_{HP} = 10.6, 3H, CH_3O^{II}), 4.74 (d, J_{HP} = 21.4, 1H, CHP), 7.23 (t, J_{HH} = 7.6, 1H, C_4H), 7.32 (t, J_{HH} = 7.5, 1H, C_5H), 7.38 (d, J_{HH} = 7.9, 1H, C_6H), 7.64 (d, J_{HH} = 7.8, 1H, C_3H); $[M+H]^+_{found}$ = 306.1029, $C_{13}H_{22}NO_3PCl$ requires 306.1020.

Diethyl ((butylamino)(2-chlorophenyl)methyl)phosphonate (6b)

Yield: 72% (0.24 g) of compound **6b** as colorless oil; ^{31}P NMR ($CDCl_3$) δ 21.4; ^{13}C NMR ($CDCl_3$) 13.9 ($\mathit{C}H_3CH_2CH_2$), 16.2 (d, $^3J_{CP}$ = 5.8, $\mathit{C}H_3CH_2O^I$), 16.5 (d, $^3J_{CP}$ = 5.9, $\mathit{C}H_3CH_2O^{II}$), 20.2 ($CH_3\mathit{C}H_2CH_2$), 32.0 (d, J_{CP} = 0.6, $\mathit{C}H_2CH_2N$), 47.6 (d, $^3J_{CP}$ = 16.3, CH_2N), 56.1 (d, $^1J_{CP}$ = 155.3, CHP), 62.8 (d, $^2J_{CP}$ = 7.1, CH_2O^I), 63.2 (d, $^2J_{CP}$ = 6.9, CH_2O^{II}), 127.1 (d, J_{CP} = 3.0, C_5), 128.8 (d, J_{CP} = 3.0, C_3), 129.37 (d, $^3J_{CP}$ = 4.3, C_6), 129.40 (d, J_{CP} = 2.1, C_4), 134.7 (d, $^2J_{CP}$ = 1.6, C_1), 134.8 (d, $^3J_{CP}$ = 8.7, C_2); ^{1}H NMR ($CDCl_3$) δ 0.85 (t, J_{HH} = 7.2, 3H, $C\mathit{H}_3CH_2CH_2$), 1.11 (t, J_{HH} = 7.1, 3H, $C\mathit{H}_3CH_2O^I$), 1.22-1.48 [1.33 (t, J_{HH} = 7.1, $C\mathit{H}_3CH_2O^{II}$) overlapped by the multiplet of $C\mathit{H}_3C\mathit{H}_2CH_2$ total int. 7H], 1.88 (bs, 1H, NH), 2.47 (t, J_{HH} = 6.9, 2H, CH_2N), 3.73-3.87 (m, 1H, CH_A, CH_2O^I), 3.87-4.02 (m, 1H, CH_B, CH_2O^I), 4.09-4.31 (m, 2H, CH_2O^{II}), 4.70 (d, J_{HP} = 21.3, 1H, CHP), 7.21 (t, J_{HH} = 7.6, 1H, C_4H), 7.29 (d, J_{HH} = 7.7, 1H, C_6H), 7.35 (t, J_{HH} = 7.8, 1H, C_5H), 7.66 (d, J_{HH} = 7.7, 1H, C_3H); $[M+H]^+_{found}$ = 334.1318, $C_{15}H_{26}NO_3PCl$ requires 334.1333.

Dibutyl ((butylamino)(2-chlorophenyl)methyl)phosphonate (6c)

Yield: 74% (0.29 g) of compound **6c** as colorless oil; ^{31}P NMR ($CDCl_3$) δ 23.2; ^{13}C NMR ($CDCl_3$) 13.5 ($\mathit{C}H_3(CH_2)_3O^I$), 13.6 ($\mathit{C}H_3(CH_2)_3O^{II}$), 13.9 ($\mathit{C}H_3(CH_2)_3N$), 18.5 ($\mathit{C}H_2(CH_2)_2O^I$),

18.7 ($CH_2(CH_2)_2O^{II}$), 20.2 ($CH_2(CH_2)_2N$), 32.0 (d, J_{CP} = 0.6, CH_2CH_2N), 32.4 (d, $^3J_{CP}$ = 5.9, $CH_2CH_2O^I$), 32.7 (d, $^3J_{CP}$ = 5.8, $CH_2CH_2O^{II}$), 47.6 (d, $^3J_{CP}$ = 16.4, CH_2N), 56.1 (d, $^1J_{CP}$ = 155.6, CHP), 66.4 (d, $^2J_{CP}$ = 7.4, CH_2O^I), 66.9 (d, $^2J_{CP}$ = 7.1, CH_2O^{II}), 127.1 (d, J_{CP} = 2.9, C_5), 128.7 (d, J_{CP} = 2.9, C_3), 129.3 (d, J_{CP} = 2.0, C_4), 129.4 (d, $^3J_{CP}$ = 4.3, C_6), 134.7 (d, $^3J_{CP}$ = 8.8, C_2), 134.9 (d, $^2J_{CP}$ = 1.5, C_1); ^{1}H NMR ($CDCl_3$) δ 0.82 (t, J_{HH} = 7.3, 3H, $CH_3(CH_2)_3O^I$), 0.86 (t, J_{HH} = 7.0, 3H, $CH_3(CH_2)_3N$), 0.93 (t, J_{HH} = 7.3, 3H, $CH_3(CH_2)_3O^{II}$), 1.14-1.51 (m, 10H, $CH_2CH_2CH_2N$, $CH_2CH_2CH_2O$, $CH_2CH_2O^I$), 1.60-1.72 (m, 2H, $CH_2CH_2O^{II}$), 1.88 (bs, 1H, NH), 2.47 (t, J_{HH} = 6.6, 2H, CH_2N), 3.67-3.80 (m, 1H, CH_A, CH_2O^I), 3.81-3.95 (m, 1H, CH_B, CH_2O^I), 4.03-4.23 (m, 2H, CH_2O^{II}), 4.70 (d, J_{HP} = 21.3, CHP), 7.22 (t, J_{HH} = 7.6, 1H, C_4H), 7.29 (d, J_{HH} = 6.8, 1H, C_6H), 7.35 (t, J_{HH} = 7.7, 1H, C_5H), 7.65 (d, J_{HH} = 7.7, 1H, C_3H); $[M+H]^+_{found}$ = 390.1950, $C_{19}H_{34}NO_3PCl$ requires 390.1959.

Dimethyl ((butylamino)(3-chlorophenyl)methyl)phosphonate (7a)

Yield: 80% (0.24 g) of compound **7a** as pale yellow oil; ^{31}P NMR ($CDCl_3$) δ 25.2; ^{13}C NMR ($CDCl_3$) 13.9 (CH_3CH_2), 20.2 (CH_3CH_2), 31.9 (d, J_{CP} = 0.7, CH_2CH_2N), 47.8 (d, $^3J_{CP}$ = 16.5, CH_2N), 53.6 (d, $^2J_{CP}$ = 6.9, CH_3O^I), 53.8 (d, $^2J_{CP}$ = 7.1, CH_3O^{II}), 60.5 (d, $^1J_{CP}$ = 153.1, CHP), 126.6 (d, $^3J_{CP}$ = 6.0, C_6), 128.1 (d, J_{CP} = 3.2, C_5), 128.5 (d, $^3J_{CP}$ = 6.3, C_2), 129.7 (d, J_{CP} = 2.6, C_4), 134.5 (d, $^2J_{CP}$ = 2.8, C_1), 138.4 (d, J_{CP} = 9.0, C_3); ^{1}H NMR ($CDCl_3$) δ 0.87 (t, J_{HH} = 7.2, 3H, CH_3CH_2), 1.23-1.37 (m, 2H, CH_3CH_2), 1.37-1.51 (m, 2H, CH_2CH_2N), 1.88 (bs, 1H, NH), 2.37-2.57 (m, 2H, CH_2N), 3.62 (d, J_{HP} = 10.5, 3H, CH_3O^I), 3.75 (d, J_{HP} = 10.5, 3H, CH_3O^{II}), 4.03 (d, J_{HP} = 20.3, 1H, CHP), 7.25-7.33 (m, 3H, $C_{4-6}H$), 7.41 (s, 1H, C_2H); $[M+H]^+_{found}$ = 306.1016, $C_{13}H_{22}NO_3PCl$ requires 306.1020.

Diethyl ((butylamino)(3-chlorophenyl)methyl)phosphonate (7b)

Yield: 94% (0.31 g) of compound **7b** as pale yellow oil; ^{31}P NMR ($CDCl_3$) δ 22.7; ^{13}C NMR ($CDCl_3$) 13.9 ($CH_3CH_2CH_2$), 16.3 (d, $^3J_{CP}$ = 5.8, $CH_3CH_2O^I$), 16.4 (d, $^3J_{CP}$ = 5.8, $CH_3CH_2O^{II}$), 20.3 ($CH_3CH_2CH_2$), 31.9 (d, J_{CP} = 0.5, CH_2CH_2N), 47.9 (d, $^3J_{CP}$ = 16.4, CH_2N), 60.9 (d, $^1J_{CP}$ = 152.2, CHP), 62.9 (d, $^2J_{CP}$ = 6.9, CH_2O^I), 63.1 (d, $^2J_{CP}$ = 7.1, CH_2O^{II}), 126.7 (d, $^3J_{CP}$ = 5.9, C_6), 127.9 (d, J_{CP} = 3.2, C_5), 128.6 (d, $^3J_{CP}$ = 6.1, C_2), 129.6 (d, J_{CP} = 2.6, C_4), 134.3 (d, $^2J_{CP}$ = 3.0, C_1), 138.6 (d, J_{CP} = 4.5, C_3); ^{1}H NMR ($CDCl_3$) δ 0.87 (t, J_{HH} = 7.2, 3H, $CH_3CH_2CH_2$), 1.19 (t, J_{HH} = 7.0, 3H, $CH_3CH_2O^I$), 1.24-1.37 [1.28 (t, J_{HH} = 7.1, $CH_3CH_2O^{II}$) overlapped by the multiplet of $CH_3CH_2CH_2$ total int. 5H], 1.38-1.50 (m, 2H, CH_2CH_2N), 2.02 (bs, 1H, NH), 2.38-2.58 (m, 2H, CH_2N), 3.85-4.16 [4.01 (d, J_{HP} = 19.9, CHP) overlapped by

the multiplet of CH_2O total int. 5H], 7.22-7.36 (m, 3H, $C_{4-6}H$), 7.42 (s, 1H, C_2H); $[M+H]^+_{found}$ = 334.1336, $C_{15}H_{26}NO_3PCl$ requires 334.1333.

Dibutyl ((butylamino)(3-chlorophenyl)methyl)phosphonate (7c)

Yield: 73% (0.28 g) of compound **7c** as pale yellow oil; ^{31}P NMR ($CDCl_3$) δ 22.8; ^{13}C NMR ($CDCl_3$) 13.56 ($CH_3(CH_2)_3O^I$), 13.60 ($CH_3(CH_2)_3O^{II}$), 13.9 ($CH_3(CH_2)_3N$), 18.6 ($CH_2(CH_2)_2O^I$), 18.7 ($CH_2(CH_2)_2O^{II}$), 20.2 ($CH_2(CH_2)_2N$), 32.0 (d, J_{CP} = 0.5, CH_2CH_2N), 32.5 (d, $^3J_{CP}$ = 5.9, $CH_2CH_2O^I$), 32.6 (d, $^3J_{CP}$ = 5.9, $CH_2CH_2O^{II}$), 47.9 (d, $^3J_{CP}$ = 16.4, CH_2N), 60.9 (d, $^1J_{CP}$ = 152.2, CHP), 66.6 (d, $^2J_{CP}$ = 7.2, CH_2O^I), 66.8 (d, $^2J_{CP}$ = 7.3, CH_2O^{II}), 126.7 (d, $^3J_{CP}$ = 5.9, C_6), 127.9 (d, J_{CP} = 3.2, C_5), 128.6 (d, $^3J_{CP}$ = 6.1, C_2), 129.6 (d, J_{CP} = 2.7, C_4), 134.3 (d, $^2J_{CP}$ = 2.9, C_1), 138.8 (d, J_{CP} = 4.6, C_3); ^{1}H NMR ($CDCl_3$) δ 0.866 (t, J_{HH} = 7.3, 3H, $CH_3(CH_2)_3O^I$), 0.870 (t, J_{HH} = 7.2, 3H, $CH_3(CH_2)_3N$), 0.91 (t, J_{HH} = 7.2, 3H, $CH_3(CH_2)_3O^{II}$), 1.20-1.55 (m, 10H, $CH_2CH_2CH_2N$, $CH_2CH_2CH_2O$, $CH_2CH_2O^I$), 1.55-1.64 (m, 2H, $CH_2CH_2O^{II}$), 1.85 (bs, 1H, NH), 2.38-2.57 (m, 2H, CH_2N), 3.80-3.96 (m, 2H, CH_2O^I), 3.96-4.09 [4.01 (d, J_{HP} = 20.0, CHP) overlapped by the multiplet of CH_2O^{II} total int. 3H], 7.22-7.34 (m, 3H, $C_{4-6}H$), 7.41 (s, 1H, C_2H); $[M+H]^+_{found}$ = 390.1946, $C_{19}H_{34}NO_3PCl$ requires 390.1959.

Dimethyl ((butylamino)(4-chlorophenyl)methyl)phosphonate (8a)

Yield: 75% (0.23 g) of compound **8a** as pale yellow oil; ^{31}P NMR ($CDCl_3$) δ 25.4; ^{13}C NMR ($CDCl_3$) 13.9 (CH_3CH_2), 20.3 (CH_3CH_2), 32.0 (d, J_{CP} = 0.5, CH_2CH_2N), 47.7 (d, $^3J_{CP}$ = 16.6, CH_2N), 53.5 (d, $^2J_{CP}$ = 7.0, CH_3O^I), 53.8 (d, $^2J_{CP}$ = 7.1, CH_3O^{II}), 60.3 (d, $^1J_{CP}$ = 153.4, CHP), 128.7 (d, J_{CP} = 2.6, C_3), 129.7 (d, $^3J_{CP}$ = 6.2, C_2), 133.7 (d, $^2J_{CP}$ = 3.9, C_1), 134.6 (d, J_{CP} = 4.5, C_4); ^{1}H NMR ($CDCl_3$) δ 0.86 (t, J_{HH} = 7.2, 3H, CH_3CH_2), 1.24-1.35 (m, 2H, CH_3CH_2), 1.37-1.48 (m, 2H, CH_2CH_2N), 1.85 (bs, 1H, NH), 2.35-2.55 (m, 2H, CH_2N), 3.60 (d, J_{HP} = 10.5, 3H, CH_3O^I), 3.74 (d, J_{HP} = 10.5, 3H, CH_3O^{II}), 4.04 (d, J_{HP} = 20.0, 1H, CHP), 7.30-7.40 (m, 4H, ArH); $[M+H]^+_{found}$ = 306.1014, $C_{13}H_{22}NO_3PCl$ requires 306.1020.

Diethyl ((butylamino)(4-chlorophenyl)methyl)phosphonate (8b)

Yield: 80% (0.27 g) of compound **8b** as pale yellow oil; ^{31}P NMR ($CDCl_3$) δ 23.0; ^{13}C NMR ($CDCl_3$) 13.9 ($CH_3CH_2CH_2$), 16.4 (d, $^3J_{CP}$ = 5.7, $CH_3CH_2O^I$), 16.5 (d, $^3J_{CP}$ = 5.7, $CH_3CH_2O^{II}$), 20.3 ($CH_3CH_2CH_2$), 32.0 (d, J_{CP} = 0.7, CH_2CH_2N), 47.8 (d, $^3J_{CP}$ = 16.5, CH_2N), 60.7 (d, $^1J_{CP}$ = 152.6, CHP), 62.9 (d, $^2J_{CP}$ = 7.0, CH_2O^I), 63.0 (d, $^2J_{CP}$ = 7.1, CH_2O^{II}), 128.6 (d, J_{CP} = 2.6, C_3), 129.8 (d, $^3J_{CP}$ = 6.1, C_2), 133.5 (d, $^2J_{CP}$ = 3.9, C_1), 135.0 (d, J_{CP} = 4.8, C_4); δ [S10] ($CDCl_3$) 16,16; 16,21; 16,33; 53,43; 54,72; 56,22; 63,23; 63,30; 63,33; 63,40; 113,78;

118,60; 118,74; 128,71; 128,74; 129,06; 129,11; 129,16; 129,36; 133,66; 134,53; 134,56; 145,89; 146,03; 152,64; ^{1}H NMR ($CDCl_3$) δ 0.86 (t, J_{HH} = 7.2, 3H, $CH_3CH_2CH_2$), 1.19 (t, J_{HH} = 7.1, 3H, $CH_3CH_2O^I$), 1.23-1.36 [1.28 (t, J_{HH} = 7.0, $CH_3CH_2O^{II}$) overlapped by the multiplet of $CH_3CH_2CH_2$ total int. 5H], 1.36-1.50 (m, 2H, CH_2CH_2N), 1.90 (bs, 1H, NH), 2.36-2.56 (m, 2H, CH_2N), 3.83-4.15 [4.01 (d, J_{HP} = 19.9, CHP) overlapped by the multiplet of CH_2O total int. 5H], 7.28-7.42 (m, 4H, ArH); δ [S10] ($CDCl_3$) 1,15 (t, J = 7.09, 3H), 1,28 (t, J = 7,09, 3H), 3,72–4,18 (m, 4H), 4,73 (d, J = 24,49, 1H), 6,54–6,57 (m, 2H), 6,68–6,72 (m, 1H), 7,07–7,12 (m, 2H), 7,29–7,31 (m, 2H), 7,39–7,42 (m, 2H); $[M+H]^+_{found}$ = 334.1337, $C_{15}H_{26}NO_3PCl$ requires 334.1333.

Dibutyl ((butylamino)(4-chlorophenyl)methyl)phosphonate (8c)

Yield: 81% (0.32 g) of compound **8c** as colorless oil; ^{31}P NMR ($CDCl_3$) δ 23.5; ^{13}C NMR ($CDCl_3$) 13.56 ($CH_3(CH_2)_3O^I$), 13.60 ($CH_3(CH_2)_3O^{II}$), 13.9 ($CH_3(CH_2)_3N$), 18.6 ($CH_2(CH_2)_2O^I$), 18.7 ($CH_2(CH_2)_2O^{II}$), 20.3 ($CH_2(CH_2)_2N$), 32.0 (d, J_{CP} = 0.5, CH_2CH_2N), 32.5 (d, $^3J_{CP}$ = 5.8, $CH_2CH_2O^I$), 32.6 (d, $^3J_{CP}$ = 5.8, $CH_2CH_2O^{II}$), 47.9 (d, $^3J_{CP}$ = 16.5, CH_2N), 60.7 (d, $^1J_{CP}$ = 152.7, CHP), 66.5 (d, $^2J_{CP}$ = 7.2, CH_2O^I), 66.7 (d, $^2J_{CP}$ = 7.4, CH_2O^{II}), 128.5 (d, J_{CP} = 2.6, C_3), 129.8 (d, $^3J_{CP}$ = 6.1, C_2), 133.5 (d, $^2J_{CP}$ = 4.0, C_1), 135.1 (d, J_{CP} = 4.7, C_4); ^{1}H NMR ($CDCl_3$) δ 0.86 (t, J_{HH} = 7.2, 6H, $CH_3(CH_2)_3O^I$, $CH_3(CH_2)_3N$), 0.91 (t, J_{HH} = 7.4, 3H, $CH_3(CH_2)_3O^{II}$), 1.20-1.53 (m, 10H, $CH_2CH_2CH_2N$, $CH_2CH_2CH_2O$, $CH_2CH_2O^I$), 1.53-1.68 (m, 2H, $CH_2CH_2O^{II}$), 1.81 (bs, 1H, NH), 2.36-2.56 (m, 2H, CH_2N), 3.77-3.95 (m, 2H, CH_2O^I), 3.95-4.08 [4.00 (d, J_{HP} = 19.8, CHP) overlapped by the multiplet of CH_2O^{II} total int. 3H], 7.26-7.40 (m, 4H, ArH); $[M+H]^+_{found}$ = 390.1962, $C_{19}H_{34}NO_3PCl$ requires 390.1959.

Real time monitoring of the addition of diethyl phosphite to *N*-benzylidene(butyl)amine

To the mixture of imine **1a** (30 mmol, 4.8 g) in 20 mL acetonitrile, diethyl phosphite (30 mmol, 3.9 mL) was added drop wise at rt with intensive stirring. After complete addition the mixture was heated gradually from 25 °C to 80 °C and stirred for 3.5 h at this temperature.

Single crystal X-ray diffraction measurements

A crystal of **5b** was mounted on a glass fiber. Cell parameters were determined by least-squares from the respective setting angles. Completeness was equal or greater to 2θ = 99.4% for **5b** and 99.6% for **5d**. Multi-scan absorption correction was applied to the data with minimum and maximum transmission factors of 0.809 and 1.00 for **5b** and 0.988 and 1.00 for **5d**, respectively. Initial structure models were obtained by direct methods [S13] and subsequent difference syntheses. Anisotropic full-matrix least-squares refinements on F^2 were applied for all non-hydrogen atoms and the model was refined to convergence [S13]. Hydrogen atomic positions were calculated from assumed geometries where appropriate. N–H hydrogen atoms were located from difference electron density maps. Hydrogen atoms were included in structure factor calculations and the non-trivial hydrogen positions and their isotropic displacements were refined. Other H atoms were kept riding on their anchor atoms, with isotropic displacement parameters of these hydrogen atoms were approximated from the U(eq) value of the atom they were bonded. The maximum and minimum residual electron densities in the final difference maps range 0.6 and −0.4 $e \cdot Å^{-3}$ and were normal for both. All further calculations and drawings were done by using PLATON [S14] and Diamond [S15]. Crystal structure data are deposited with the Cambridge Crystallographic Data Centre under CCDC 1508647 (**5b**) and 1508648 (**5d**) and can be obtained free of charge upon application.

Table S1: Essential crystallographic data of the **5b** and **5d** single crystal diffraction experiments and model refinements.

	**5b**	**5d**
Formula	$C_{17}H_{22}NO_3P$	$C_{27}H_{26}NO_3P$
F. W.	319.32	443.46
Space grp.	P-1 (No. 2)	P-1 (No. 2)
a, b, c [Å]	8.7262(5) 9.8627(6) 10.0063(6)	9.7723(5) 10.7780(6) 11.1060(7)
α, β, γ [^{o}]	94.125(5) 94.790(5) 91.430(5)	101.830(5) 94.026(4) 90.170(4)
V [Å^3]	855.54(9)	1141.90(11)
Z	2	2
$D_{(c)}$ [Mg/m^3]	1.240	1.290
μ(MoKα) [/mm]	0.172	0.150
Cryst. Size [mm]	0.06 x 0.35 x 0.40	0.08 x 0.15 x 0.15
Temp. (K)	173	173
Radiation λ [Å]	MoKa 0.71073	MoKa 0.71073
θ Min-Max [^{o}]	4.5, 26.4	4.2, 26.4
Tot., Uniq. Data, R(int)	10779, 3477, 0.044	14708, 4650, 0.043
Obsd data [I > 2.0 σ(I)]	2578	3473
Nref, Npar	3477, 219	4650, 315
R_1, wR^2, S	0.0524, 0.1427, 1.06	0.0427, 0.1054, 1.03
Max./ Av. Shift/Error	0.00, 0.00	0.00, 0.00
Min/Max Res.Den. [e/Å^3]	-0.34, 0.62	-0.36, 0.41

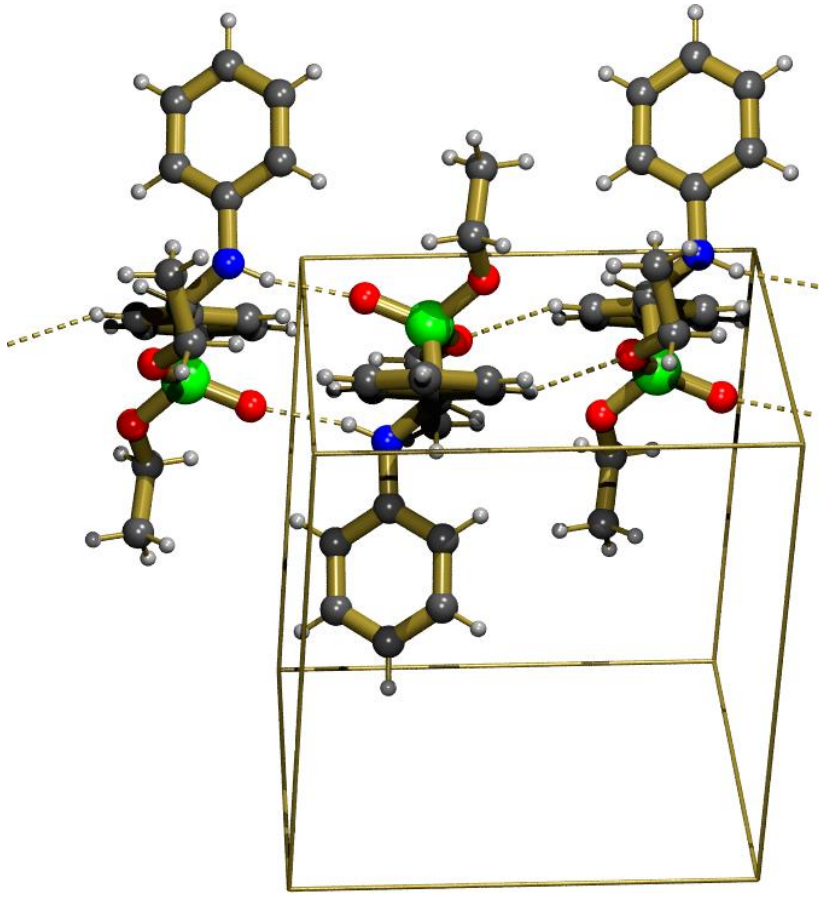

Figure S1: Pairs of **5b** molecules fused through N–H···O and C–H···O hydrogen bridges into *RS*-centrosymmetric dimeric infinite sheets. Broken lines indicate H-bridges.

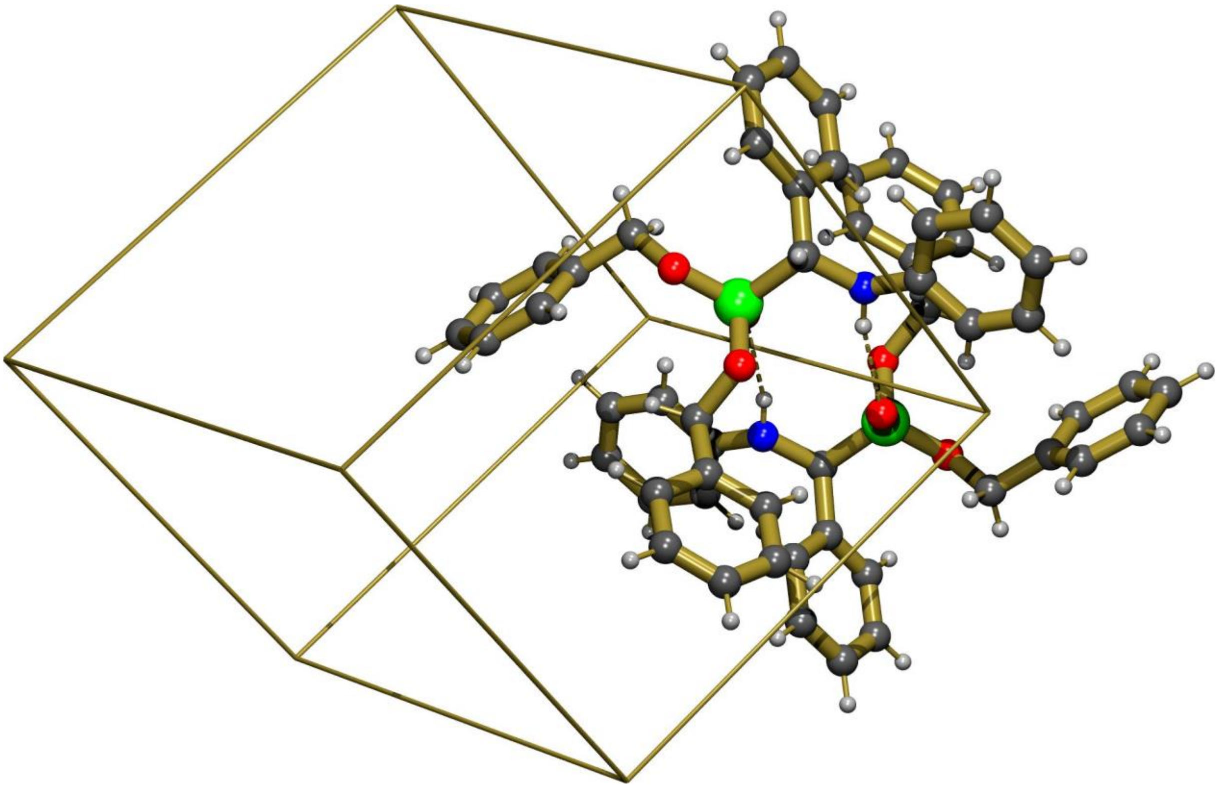

Figure S2: An isolated *RS* dimer of **5d** molecules formed around a symmetry center.

Quantum chemical calculations

In all quantum chemical calculations, the B3LYP/6-31G(d,p) method [S16-S20] was used implemented in the Gaussian09 program package [S21]. During the calculations including geometry optimizations, scanning and IRC optimizations, transition-state determinations and frequency calculations, the IEFPCM [S22,S23] implicit method was used taking into account the effect of water as the solvent, approximating the dielectric constant of the reaction mixture. The reaction pathways and energetics were calculated by scanning and IRC calculations to verify that a calculated transition state indeed connects the two corresponding minima. The transition states were optimized with the QST3 method. Transition states were identified by having one imaginary frequency in the Hessian matrix. All the geometries and transition states were optimized and frequency calculations were made to assure that the structures are in a local minimum on a saddle point, respectively.

^{31}P NMR, ^{1}H NMR and ^{13}C NMR spectra

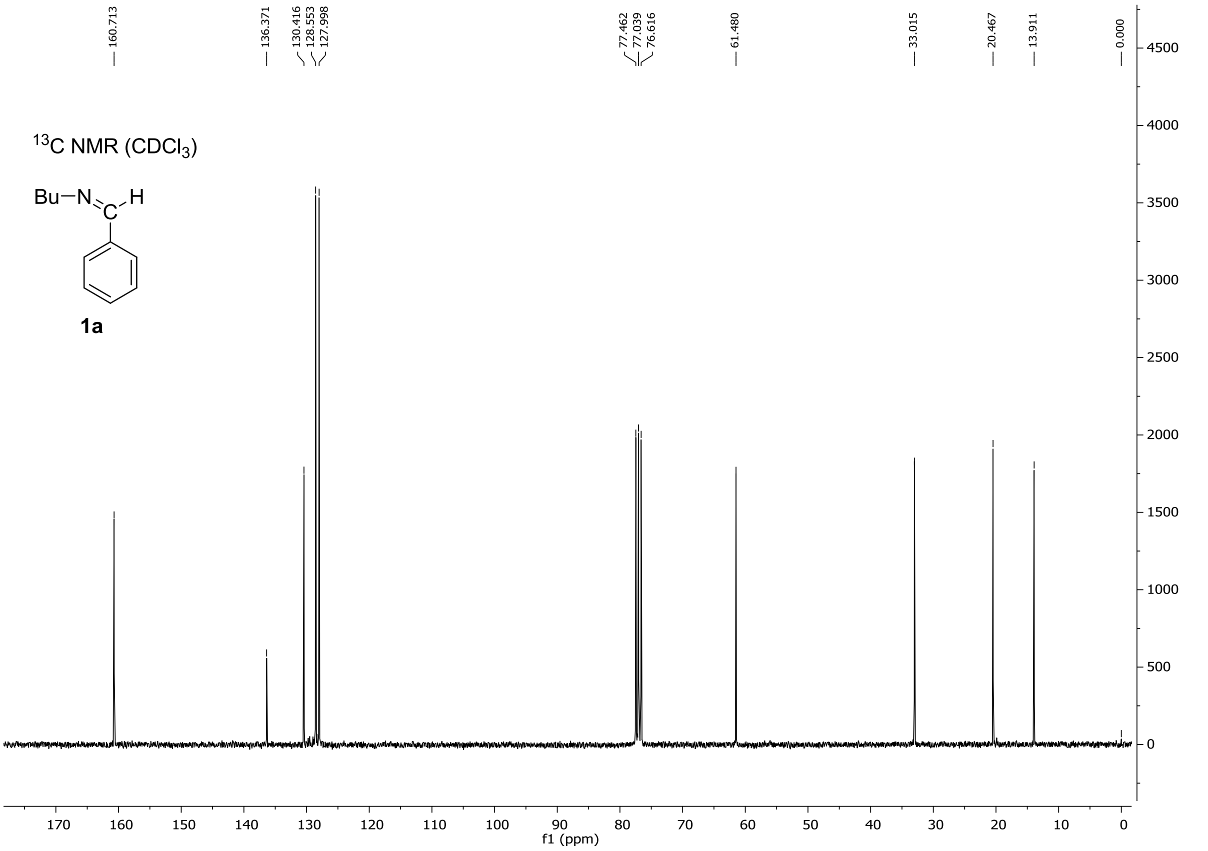

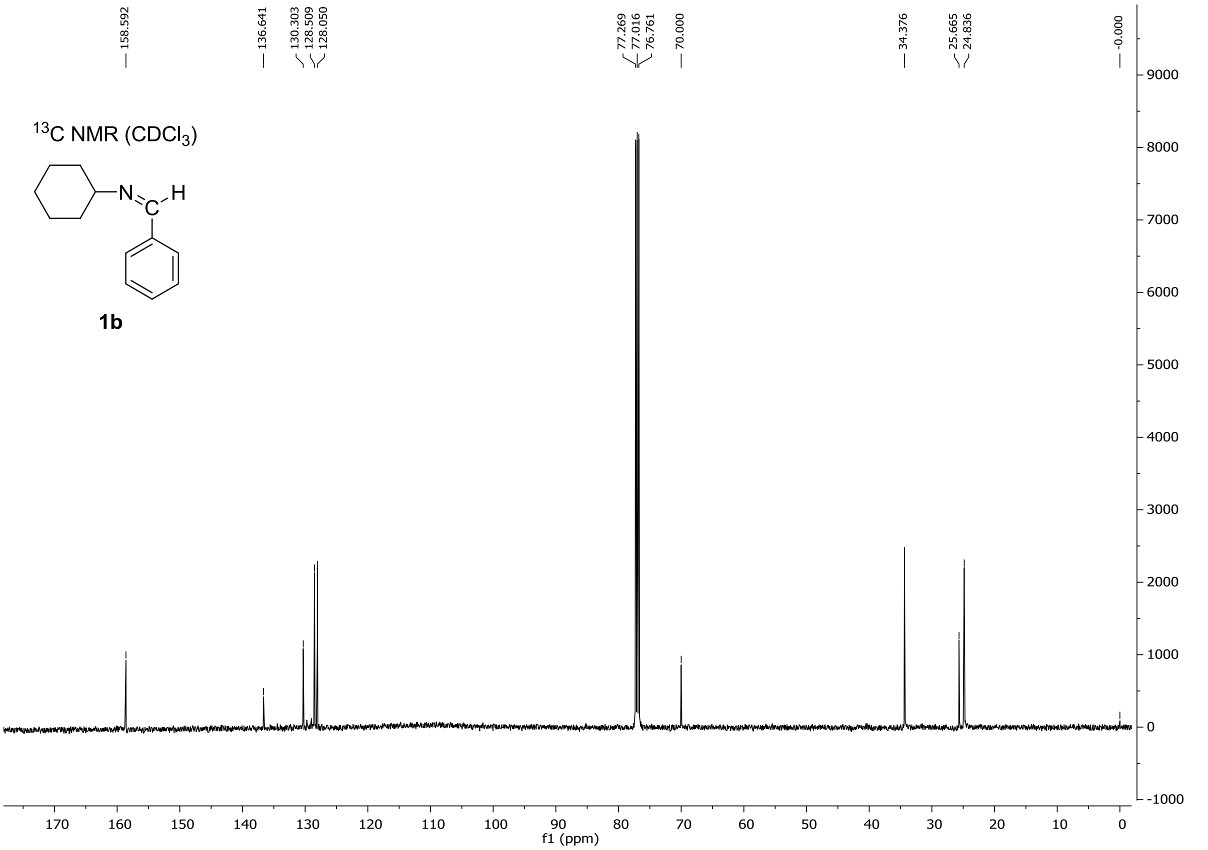

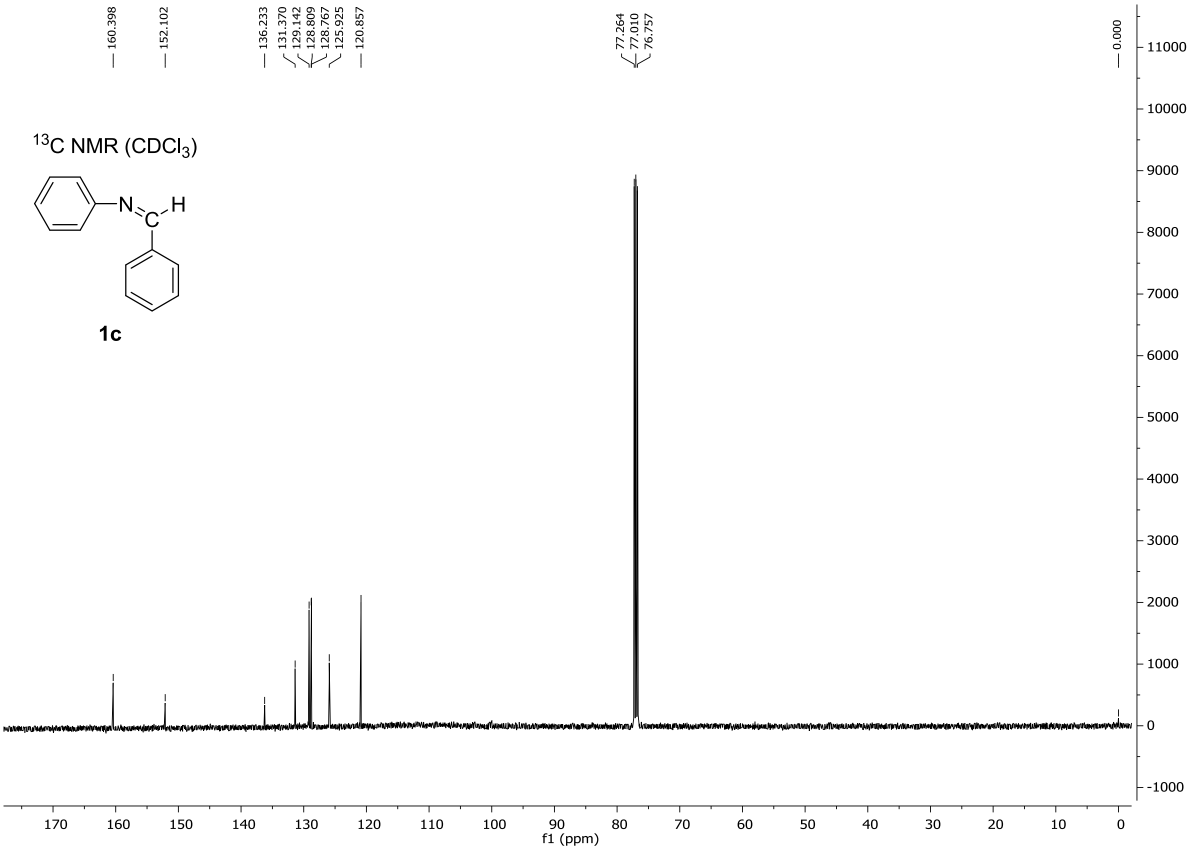
160.398
152.102
136.233
131.370
129.142
128.809
128.767
125.925
120.857
77.264
77.010
76.757
0.000
^{13}C NMR ($CDCl_3$)
N
C
H
1c
f1 (ppm)

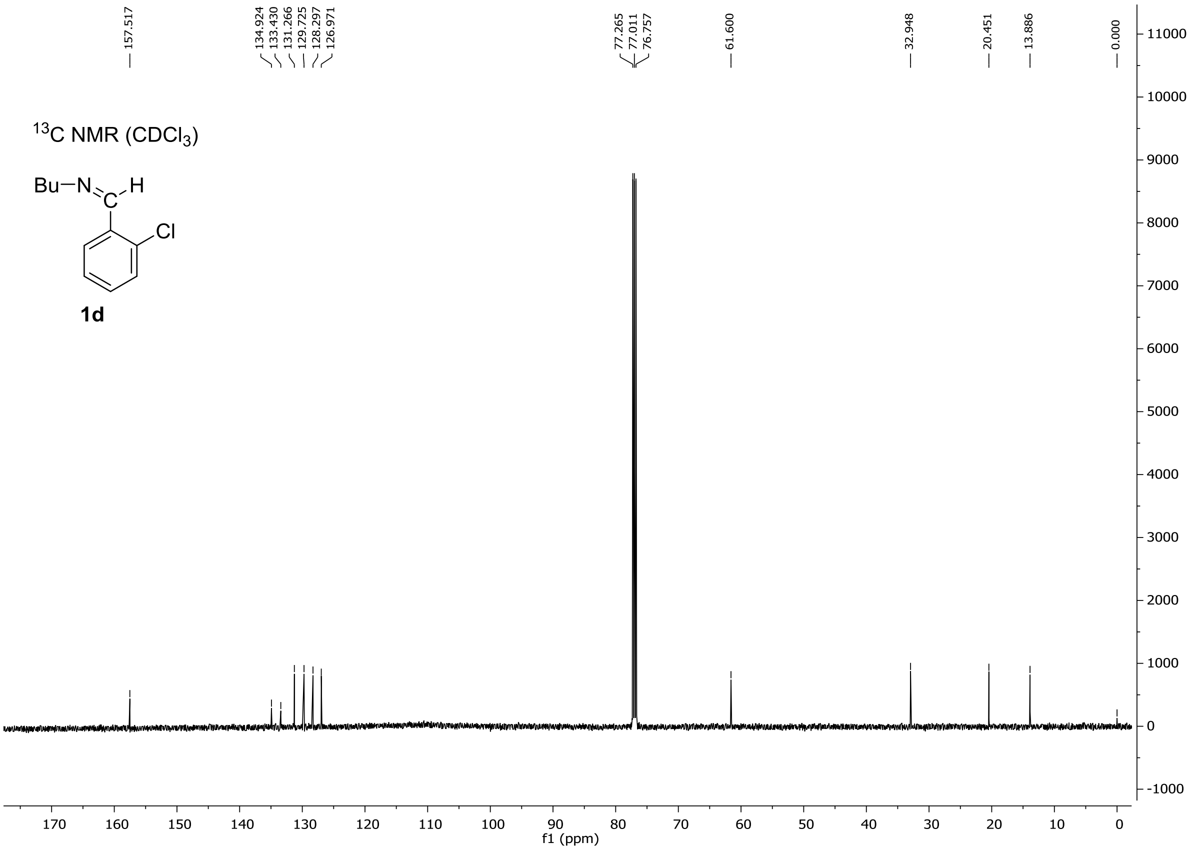
157.517
134.924
133.430
131.266
129.725
128.297
126.971
77.265
77.011
76.757
61.600
32.948
20.451
13.886
0.000
^{13}C NMR ($CDCl_3$)
Bu
N
C
H
Cl
1d
f1 (ppm)

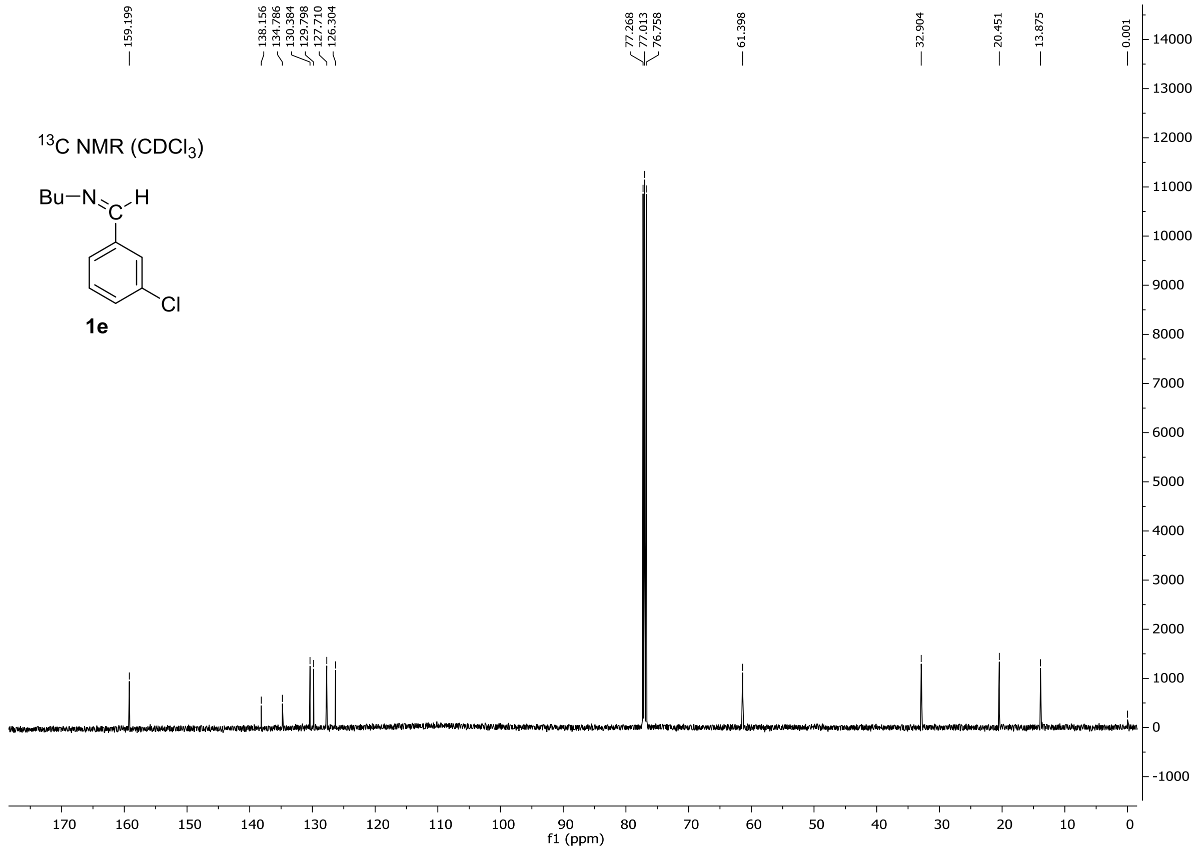

159.199
138.156
134.786
130.384
129.798
127.710
126.304
77.268
77.013
76.758
61.398
32.904
20.451
13.875
0.001
^{13}C NMR (CDCl$_3$)
Bu
N
C
H
Cl
1e
f1 (ppm)

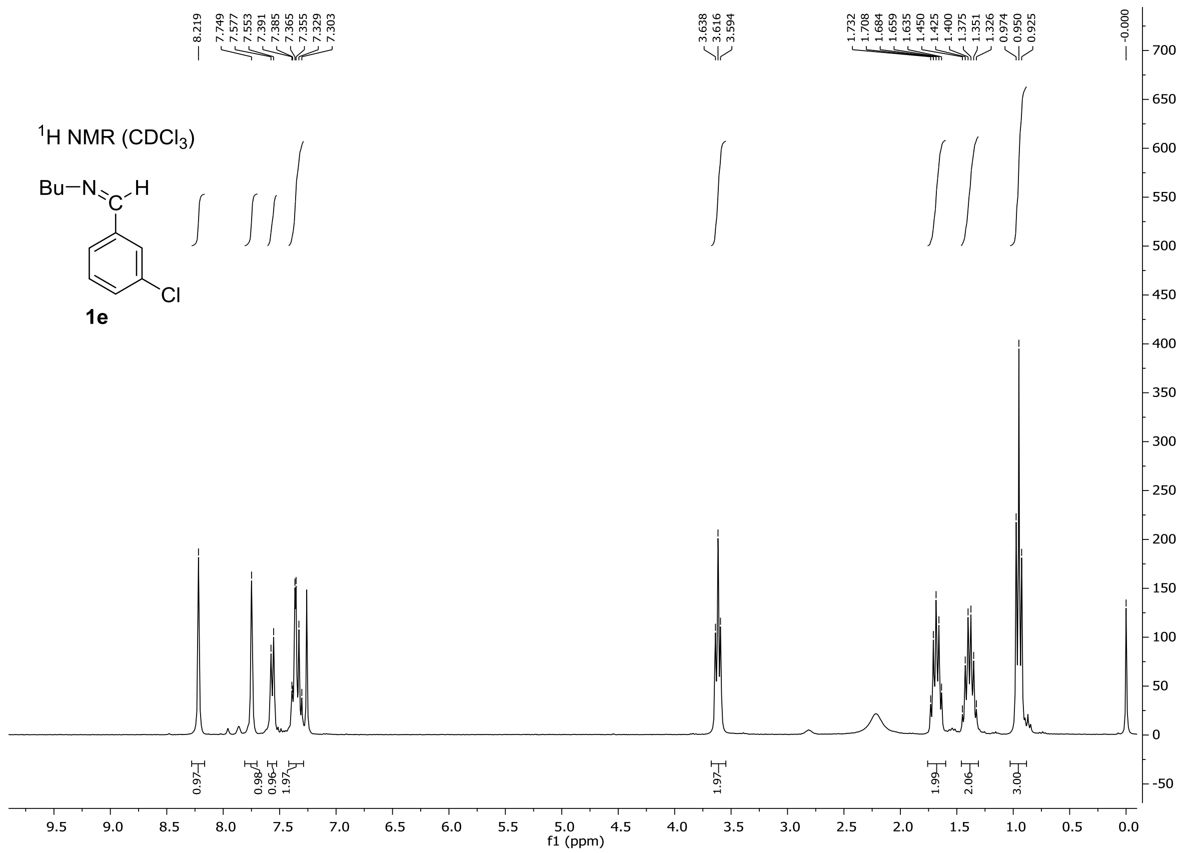

8.219
7.749
7.577
7.553
7.391
7.385
7.365
7.355
7.329
7.303
3.638
3.616
3.594
1.732
1.708
1.684
1.659
1.635
1.450
1.425
1.400
1.375
1.351
1.326
0.974
0.950
0.925
-0.000
^{1}H NMR (CDCl$_3$)
Bu
N
C
H
Cl
1e
0.97
0.98
0.96
1.97
1.97
1.99
2.06
3.00
f1 (ppm)

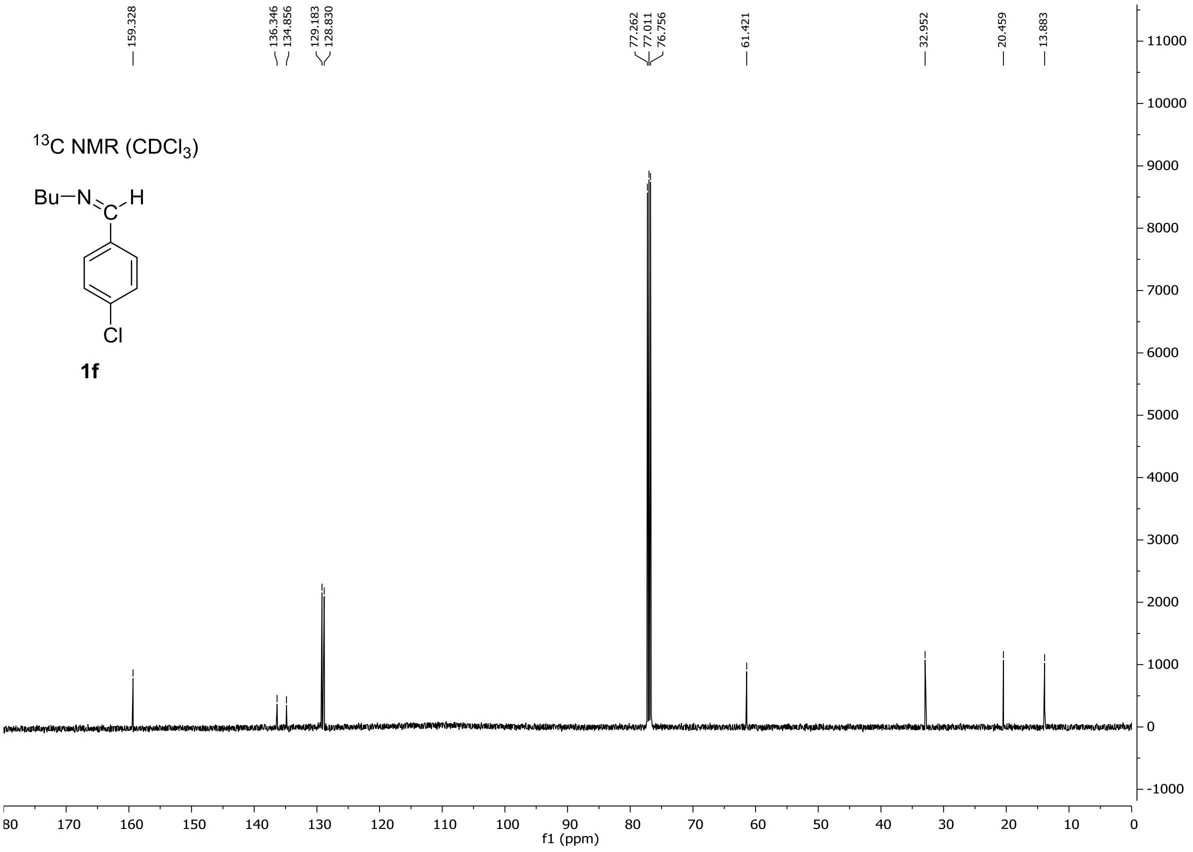

159.328
136.346
134.856
129.183
128.830
77.262
77.011
76.756
61.421
32.952
20.459
13.883
^{13}C NMR (CDCl$_3$)
Bu–N=C–H
Cl
f1 (ppm)

1f

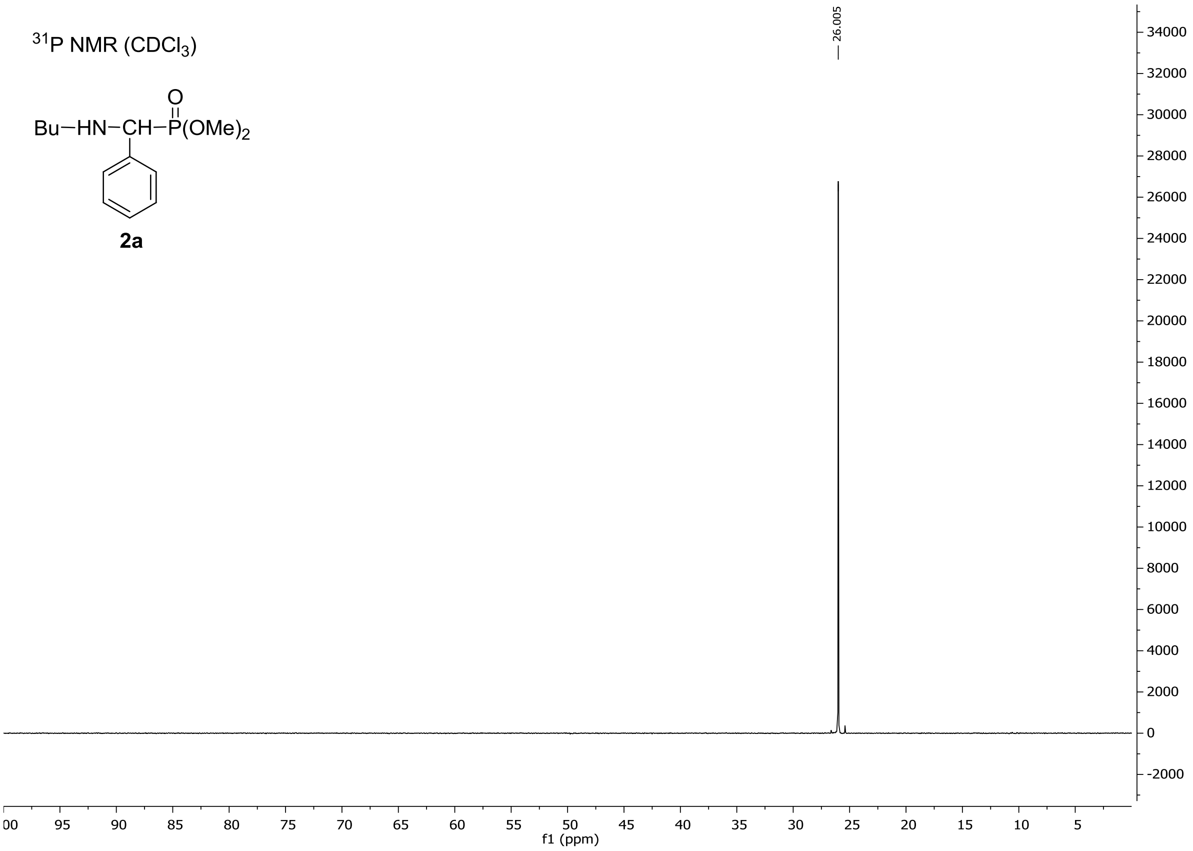

26.005
^{31}P NMR (CDCl$_3$)
O
Bu–HN–CH–P(OMe)$_2$
f1 (ppm)

2a

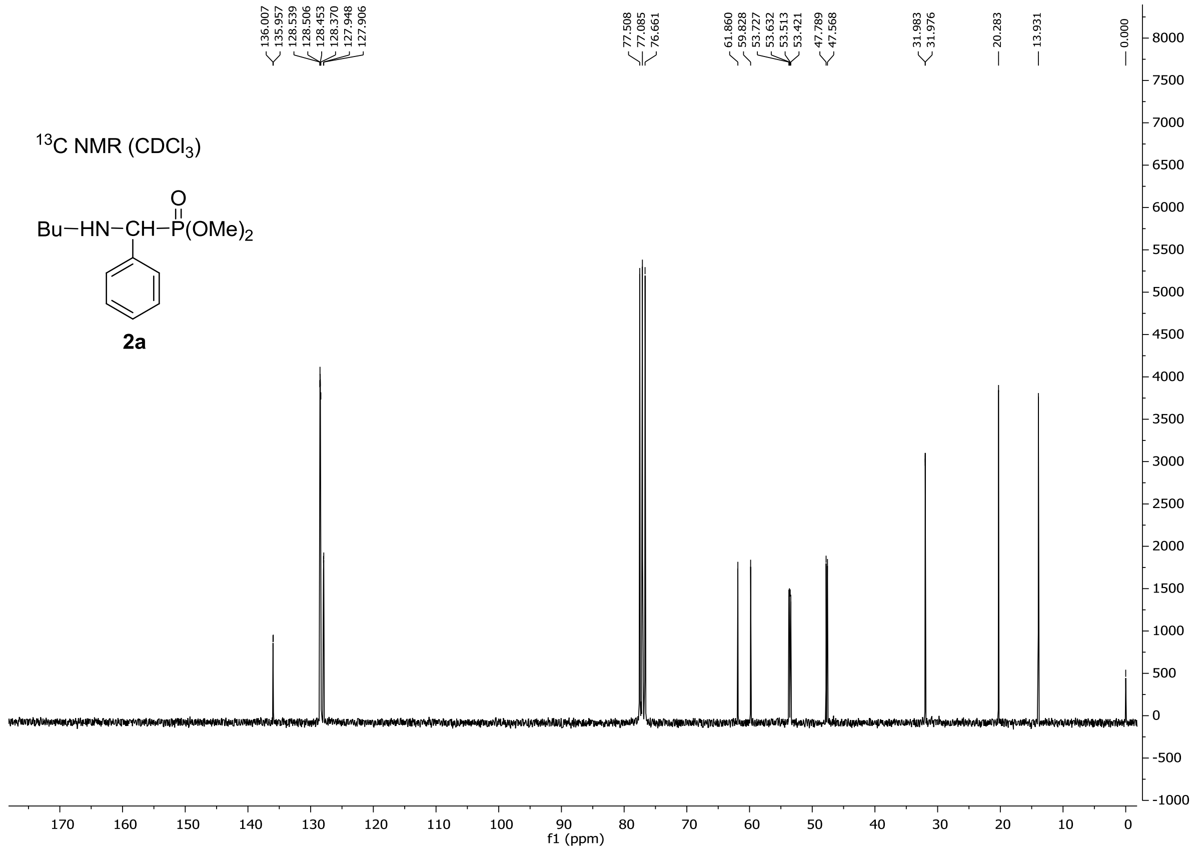
13C NMR (CDCl3)
Bu–HN–CH–P(OMe)2
O
2a
136.007
135.957
128.539
128.506
128.453
128.370
127.948
127.906
77.508
77.085
76.661
61.860
59.828
53.727
53.632
53.513
53.421
47.789
47.568
31.983
31.976
20.283
13.931
0.000
f1 (ppm)

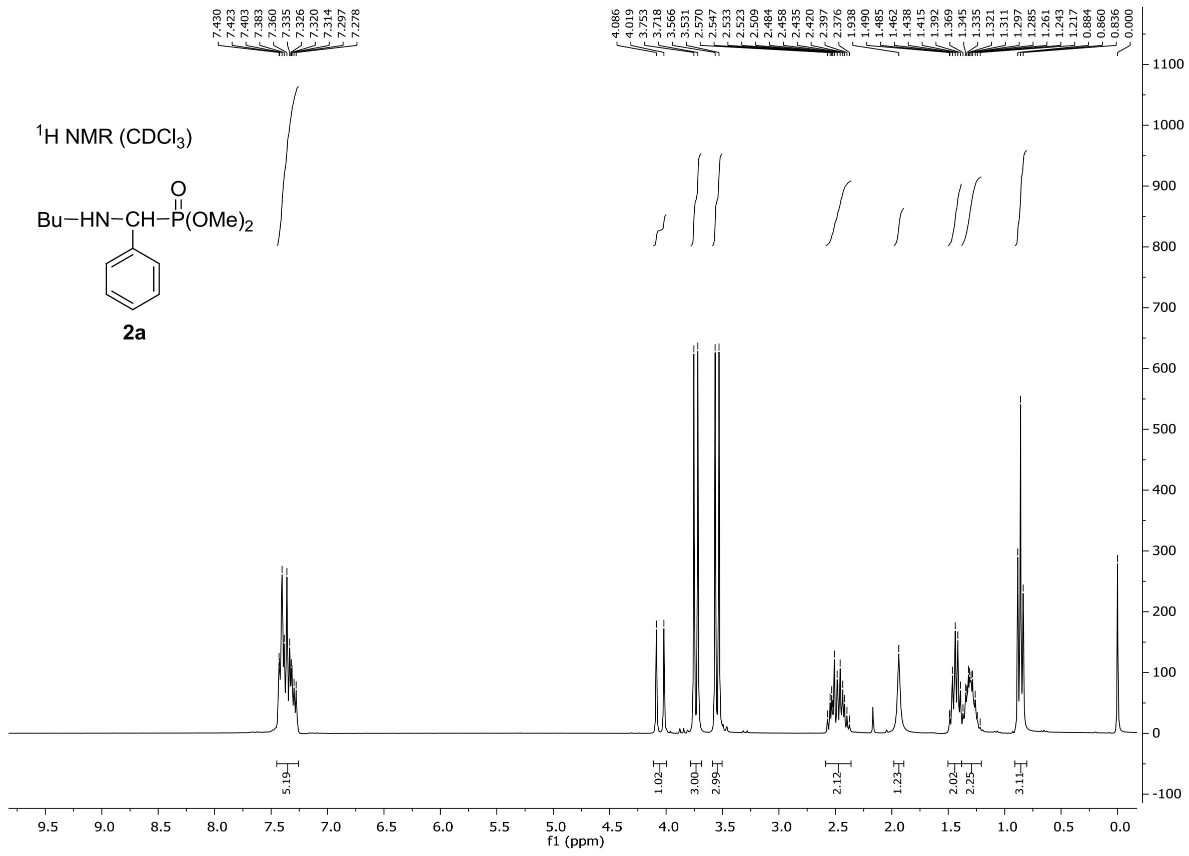
1H NMR (CDCl3)
Bu–HN–CH–P(OMe)2
O
2a
7.430
7.423
7.403
7.383
7.360
7.335
7.326
7.320
7.314
7.297
7.278
4.086
4.019
3.753
3.718
3.566
3.531
2.570
2.547
2.533
2.523
2.509
2.484
2.458
2.435
2.420
2.397
2.376
1.938
1.490
1.485
1.462
1.438
1.415
1.392
1.369
1.345
1.335
1.321
1.311
1.297
1.285
1.261
1.243
1.217
0.884
0.860
0.836
0.000
5.19
1.02
3.00
2.99
2.12
1.23
2.02
2.25
3.11
f1 (ppm)

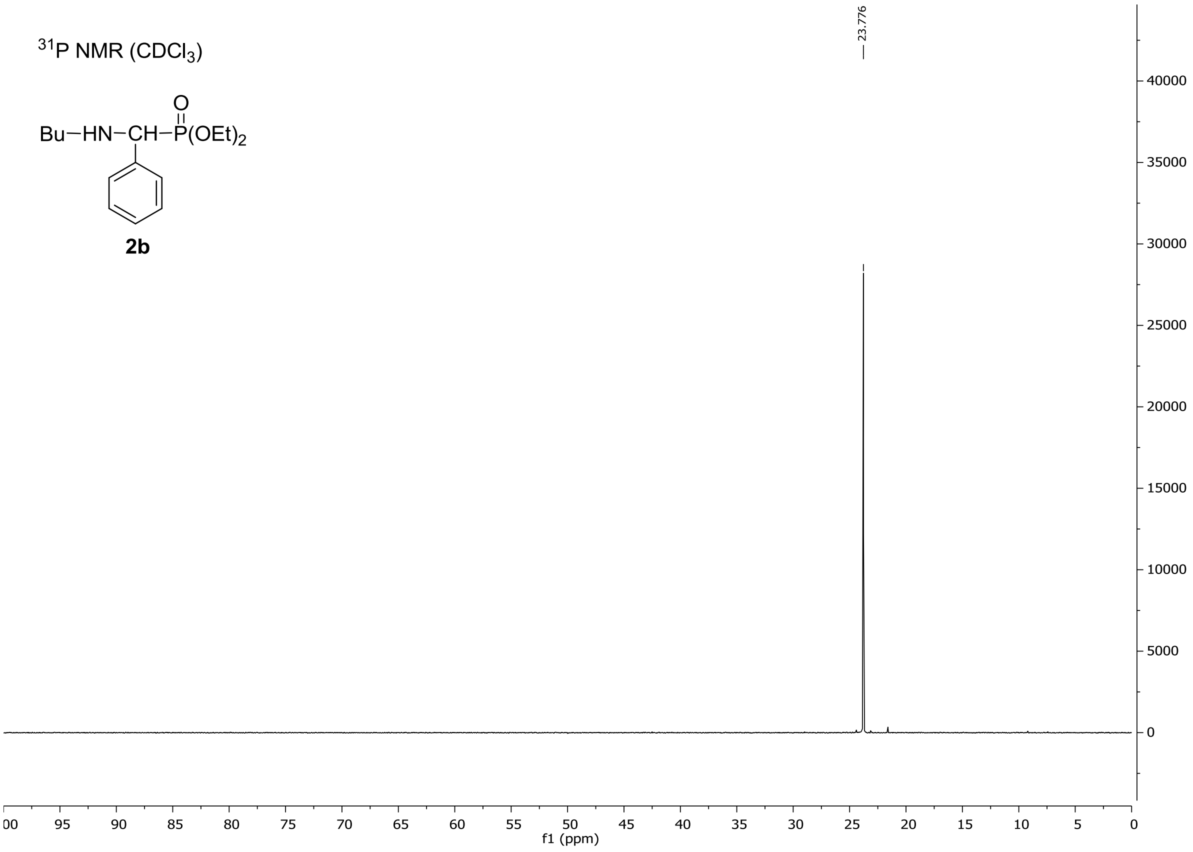
^{31}P NMR ($CDCl_3$)
Bu–HN–CH–P(OEt)$_2$
O
2b
23.776
f1 (ppm)

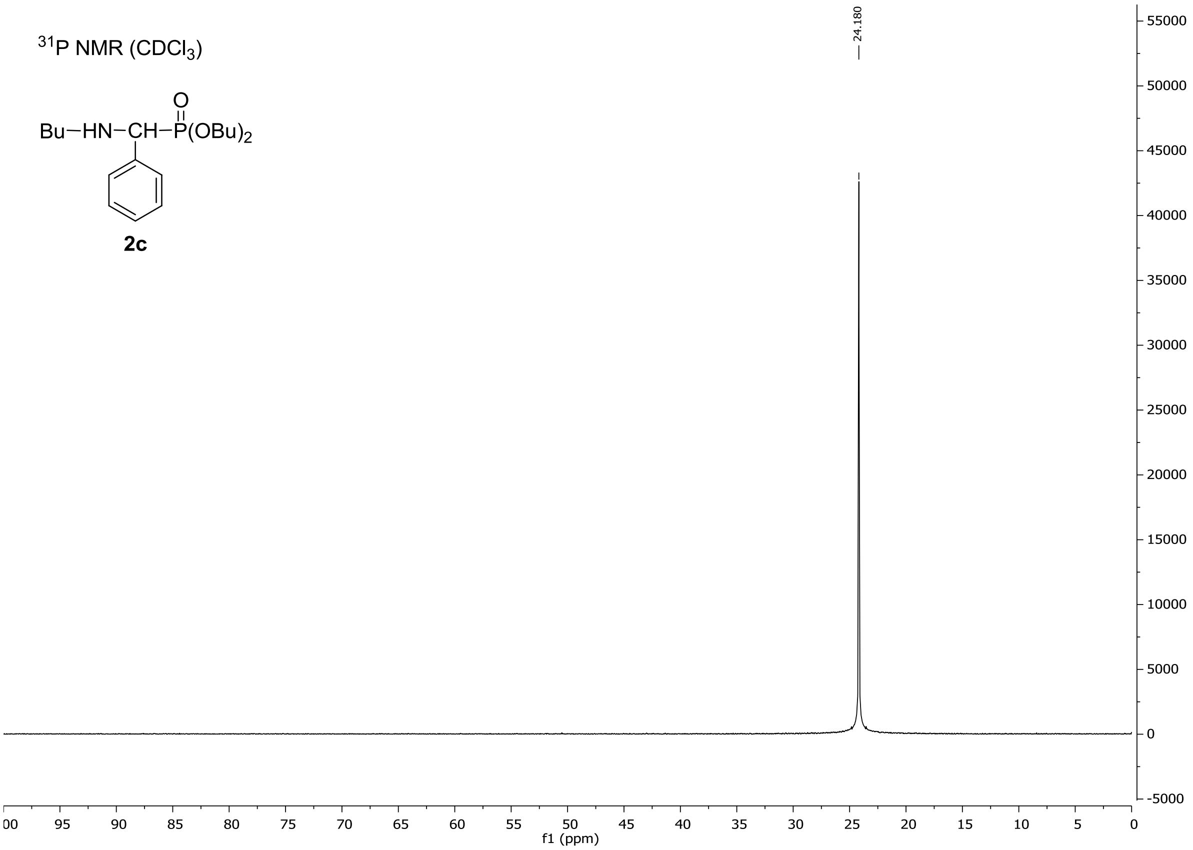
^{31}P NMR ($CDCl_3$)
Bu–HN–CH–P(OBu)$_2$
O
2c
24.180
f1 (ppm)

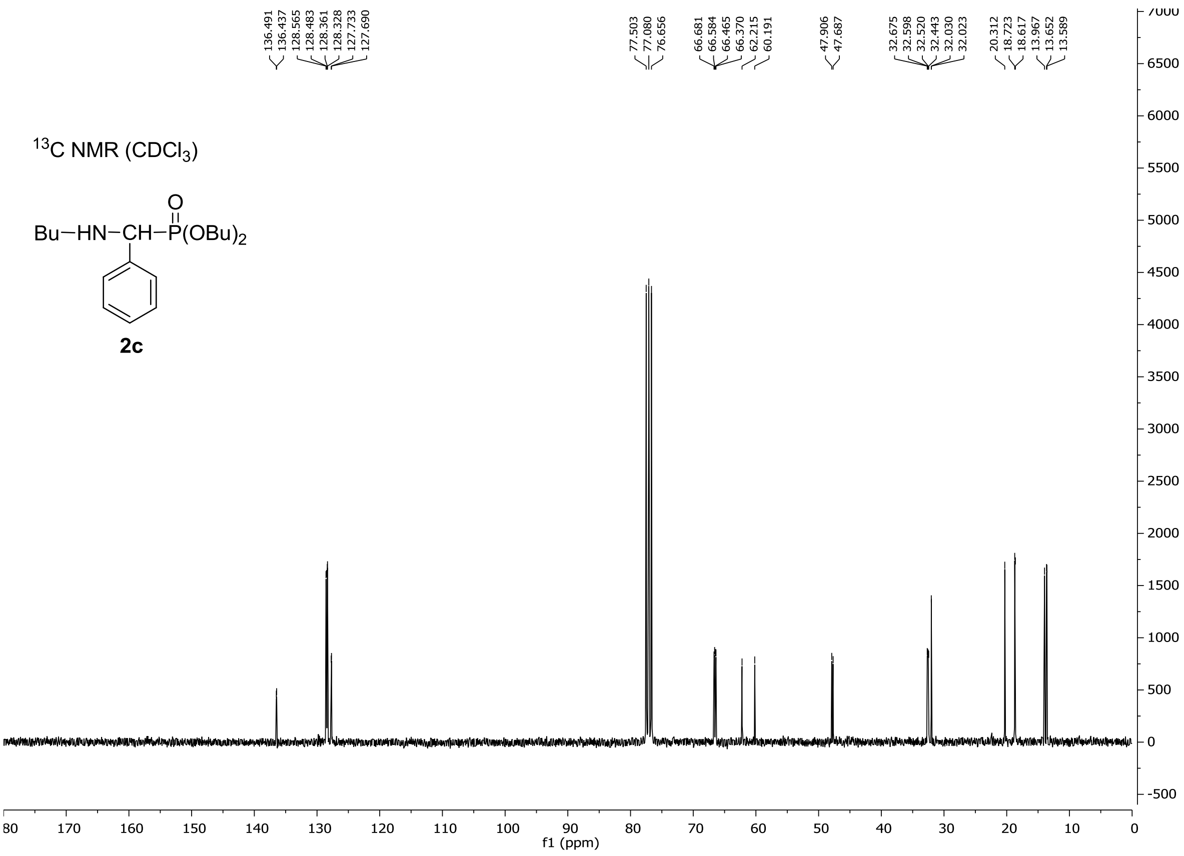

^{13}C NMR (CDCl$_3$)
Bu–HN–CH–P(OBu)$_2$
O
2c
f1 (ppm)

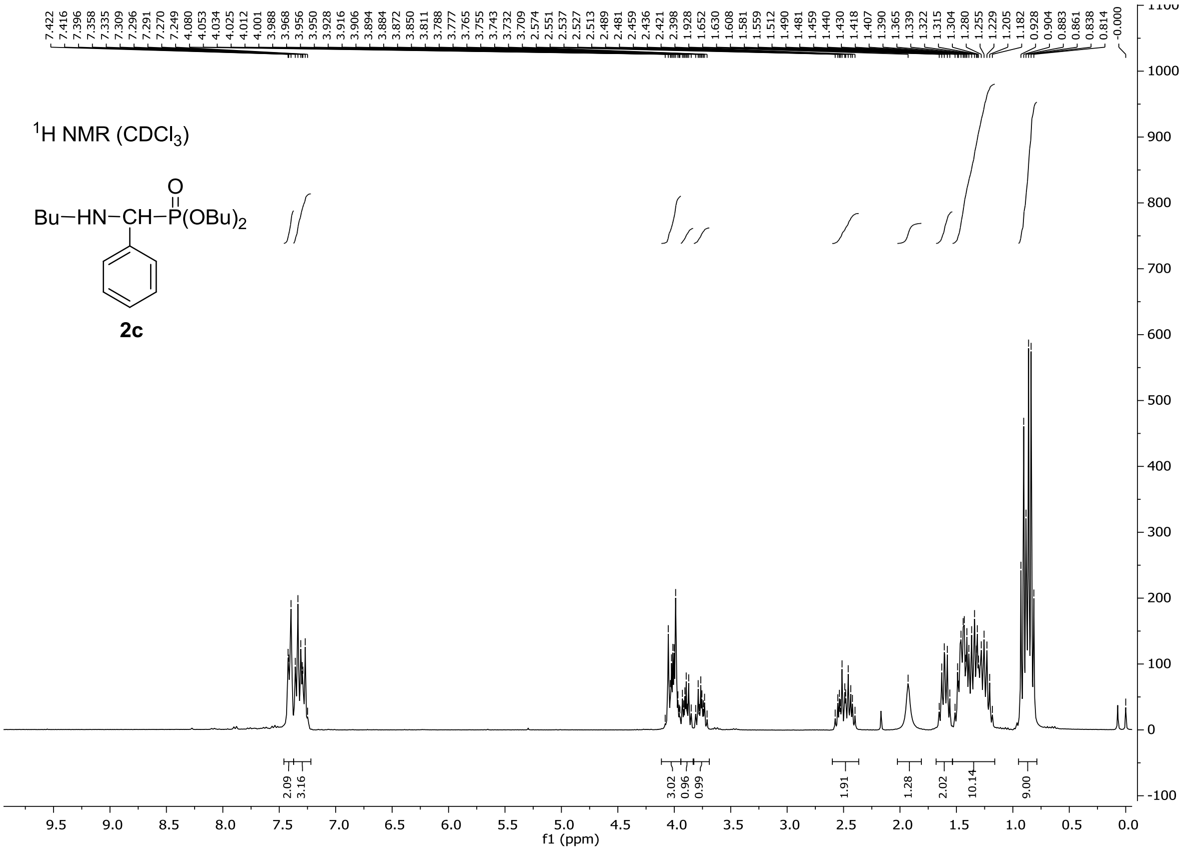

^{1}H NMR (CDCl$_3$)
Bu–HN–CH–P(OBu)$_2$
O
2c
f1 (ppm)

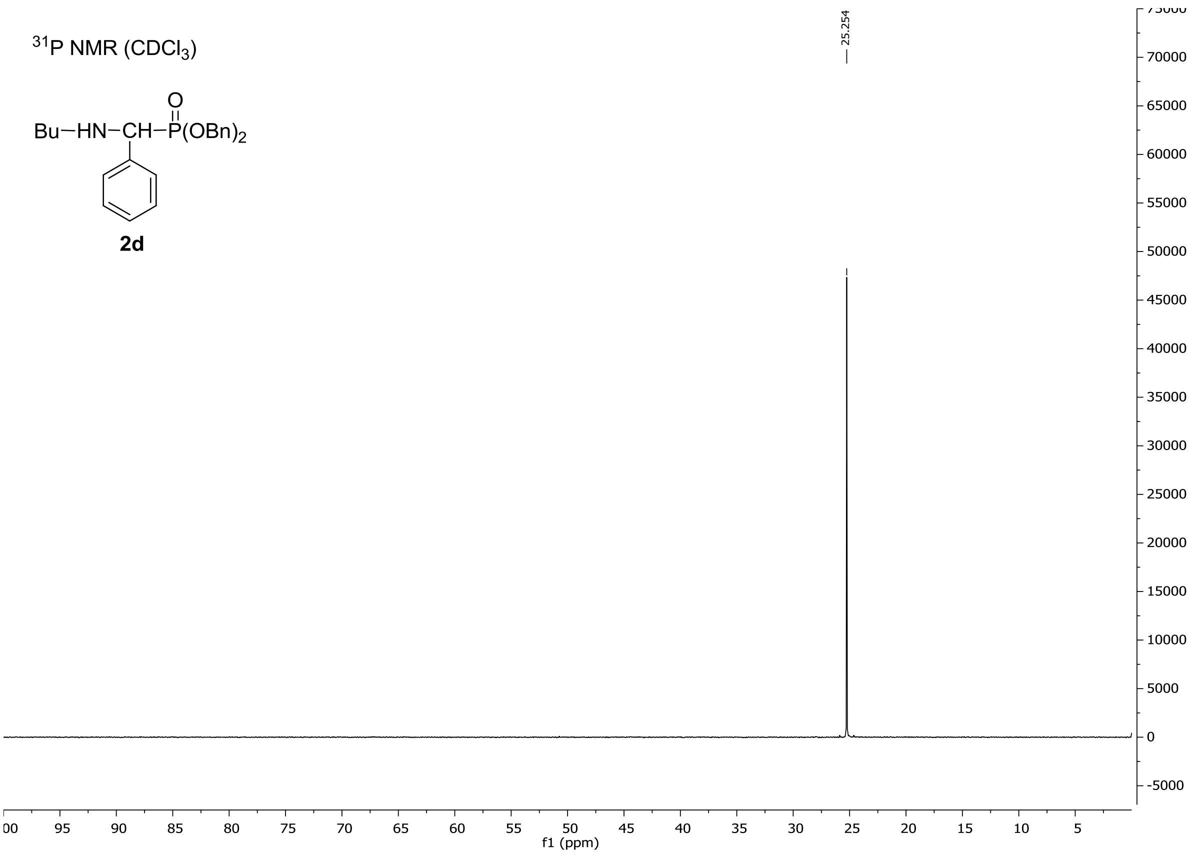

^{31}P NMR ($CDCl_3$)
Bu−HN−CH−P(OBn)$_2$
O
2d
25.254
f1 (ppm)

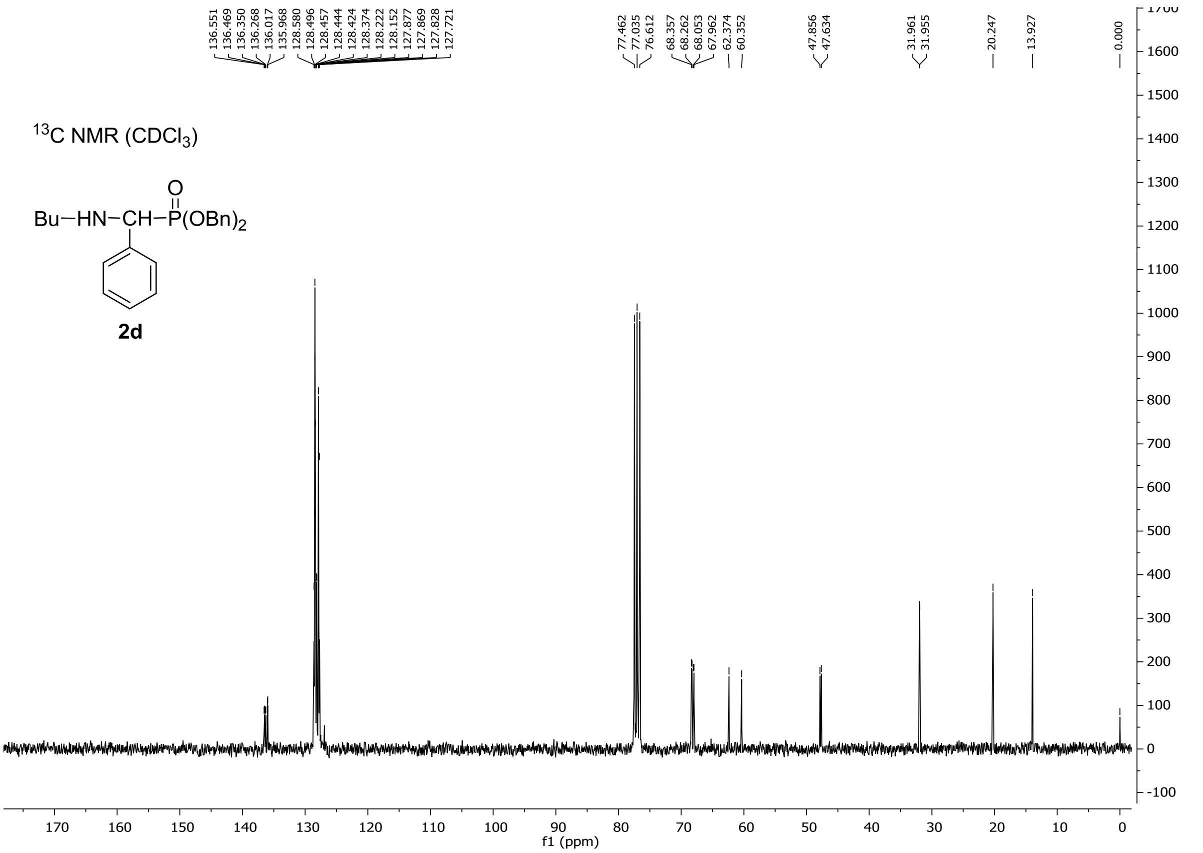

^{13}C NMR ($CDCl_3$)
Bu−HN−CH−P(OBn)$_2$
O
2d
136.551
136.469
136.350
136.268
136.017
135.968
128.580
128.496
128.457
128.444
128.424
128.374
128.222
128.152
127.877
127.869
127.828
127.721
77.462
77.035
76.612
68.357
68.262
68.053
67.962
62.374
60.352
47.856
47.634
31.961
31.955
20.247
13.927
0.000
f1 (ppm)

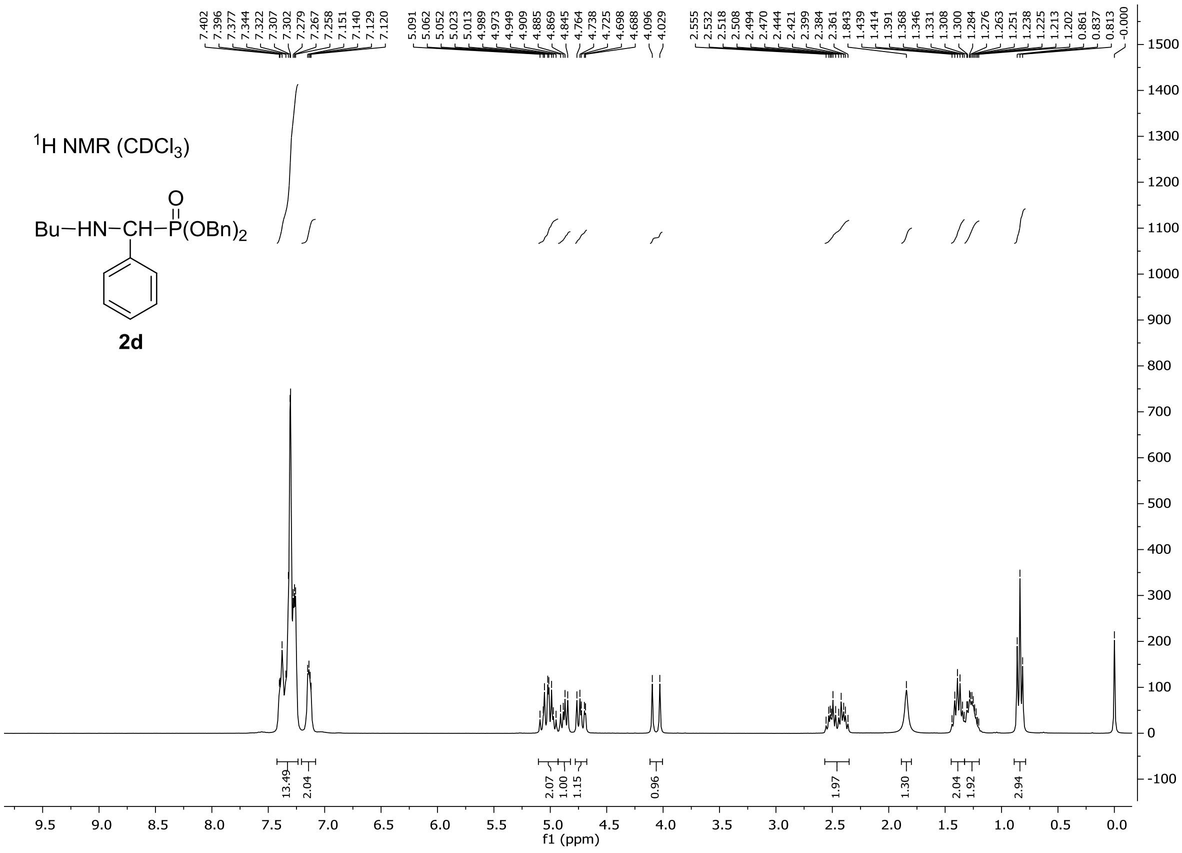
1H NMR (CDCl3)
Bu−HN−CH−P(OBn)2
O
2d
7.402 7.396 7.377 7.344 7.322 7.307 7.302 7.279 7.267 7.258 7.151 7.140 7.129 7.120
5.091 5.062 5.052 5.023 5.013 4.989 4.973 4.949 4.909 4.885 4.869 4.845 4.764 4.738 4.725 4.698 4.688 4.096 4.029
2.555 2.532 2.518 2.508 2.494 2.470 2.444 2.421 2.399 2.384 2.361 1.843 1.439 1.414 1.391 1.368 1.346 1.331 1.308 1.300 1.284 1.276 1.263 1.251 1.238 1.225 1.213 1.202 0.861 0.837 0.813 -0.000
13.49 2.04 2.07 1.00 1.15 0.96 1.97 1.30 2.04 1.92 2.94
f1 (ppm)

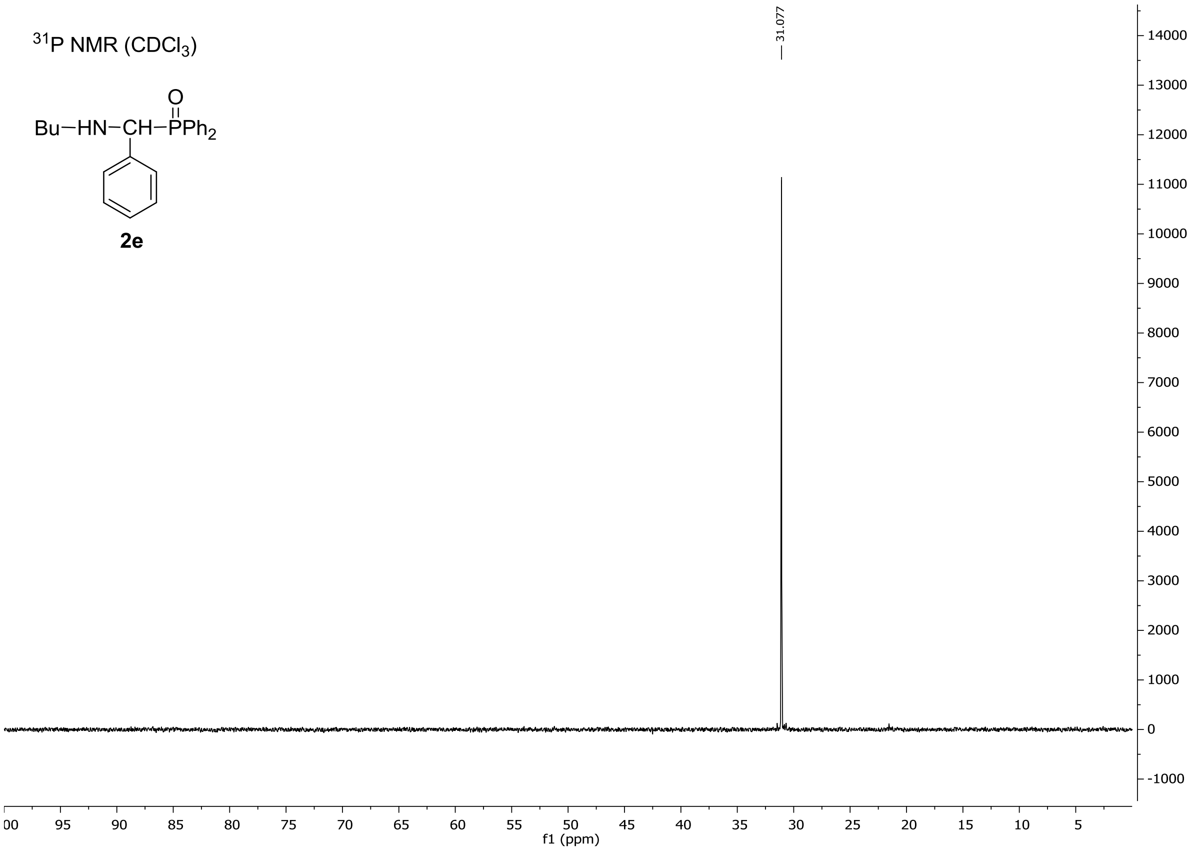
31P NMR (CDCl3)
Bu−HN−CH−PPh2
O
2e
31.077
f1 (ppm)

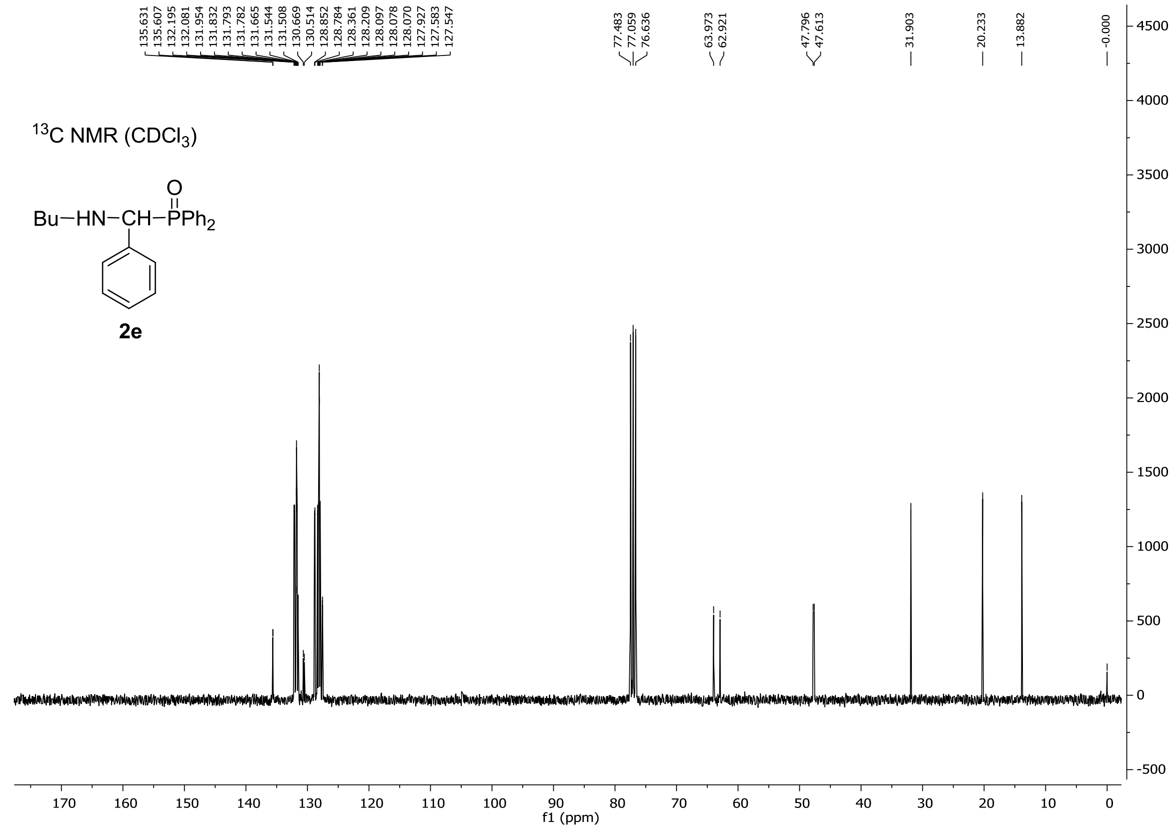
13C NMR (CDCl3)
Bu−HN−CH−P(=O)Ph2
2e
135.631 135.607 132.195 132.081 131.954 131.832 131.793 131.782 131.665 131.544 131.508 130.669 130.514 128.852 128.784 128.361 128.209 128.097 128.078 128.070 127.927 127.583 127.547
77.483 77.059 76.636
63.973 62.921
47.796 47.613
31.903
20.233
13.882
-0.000
f1 (ppm)
170 160 150 140 130 120 110 100 90 80 70 60 50 40 30 20 10 0
4500 4000 3500 3000 2500 2000 1500 1000 500 0 -500

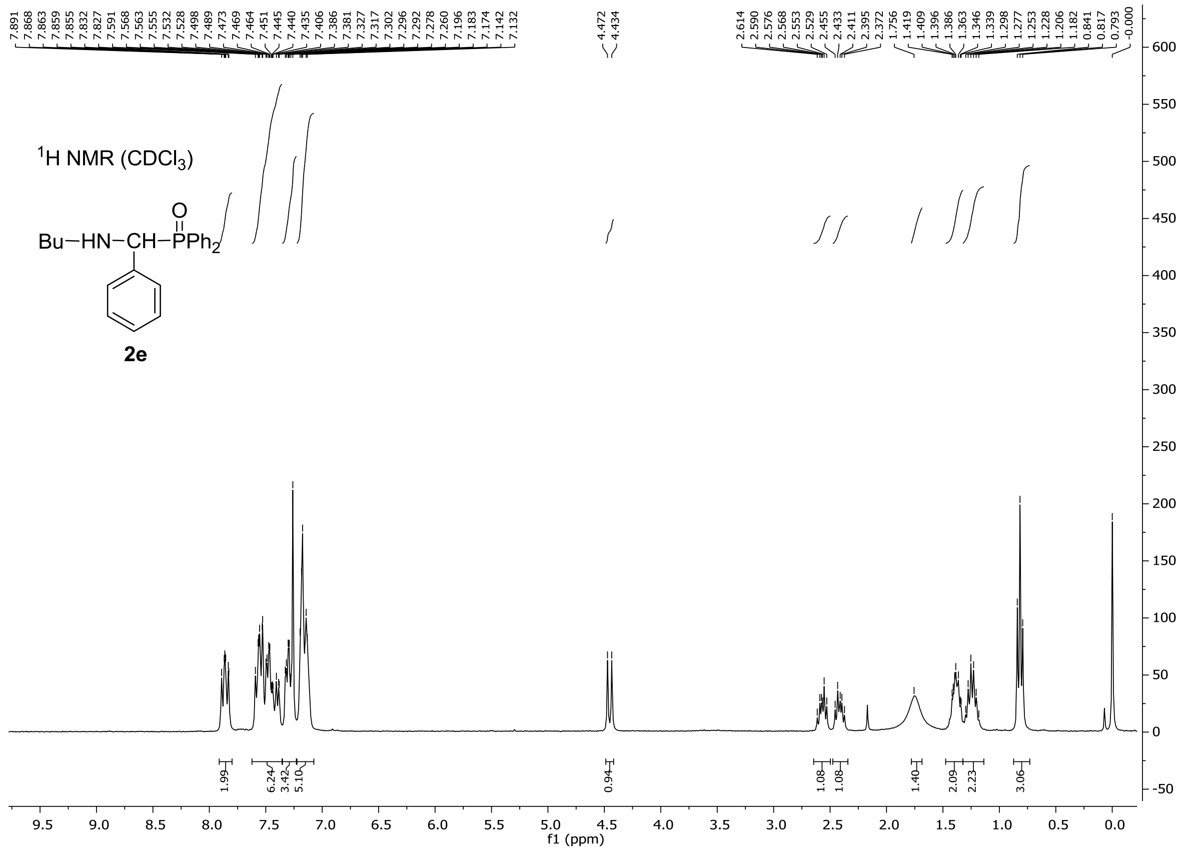
1H NMR (CDCl3)
Bu−HN−CH−P(=O)Ph2
2e
7.891 7.868 7.863 7.859 7.855 7.832 7.827 7.591 7.568 7.563 7.555 7.532 7.528 7.498 7.489 7.473 7.469 7.464 7.451 7.445 7.440 7.435 7.406 7.386 7.381 7.327 7.317 7.302 7.296 7.292 7.278 7.260 7.196 7.183 7.174 7.142 7.132
4.472 4.434
2.614 2.590 2.576 2.568 2.553 2.529 2.455 2.433 2.411 2.395 2.372 1.756 1.419 1.409 1.396 1.386 1.363 1.346 1.339 1.298 1.277 1.253 1.228 1.206 1.182 0.841 0.817 0.793 -0.000
1.99 6.24 3.42 5.10 0.94 1.08 1.08 1.40 2.09 2.23 3.06
f1 (ppm)
9.5 9.0 8.5 8.0 7.5 7.0 6.5 6.0 5.5 5.0 4.5 4.0 3.5 3.0 2.5 2.0 1.5 1.0 0.5 0.0
600 550 500 450 400 350 300 250 200 150 100 50 0 -50

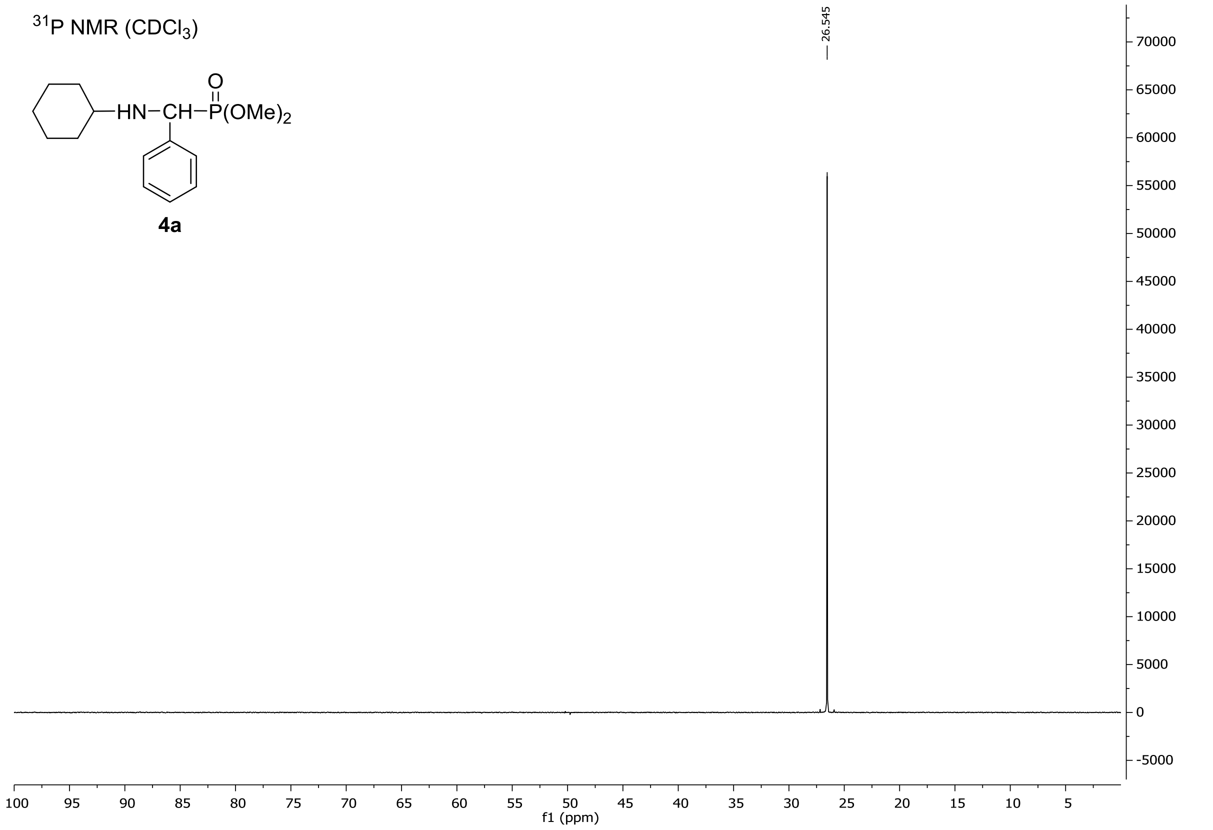
^{31}P NMR ($CDCl_3$)
HN−CH−P(OMe)$_2$
O
4a
26.545
f1 (ppm)

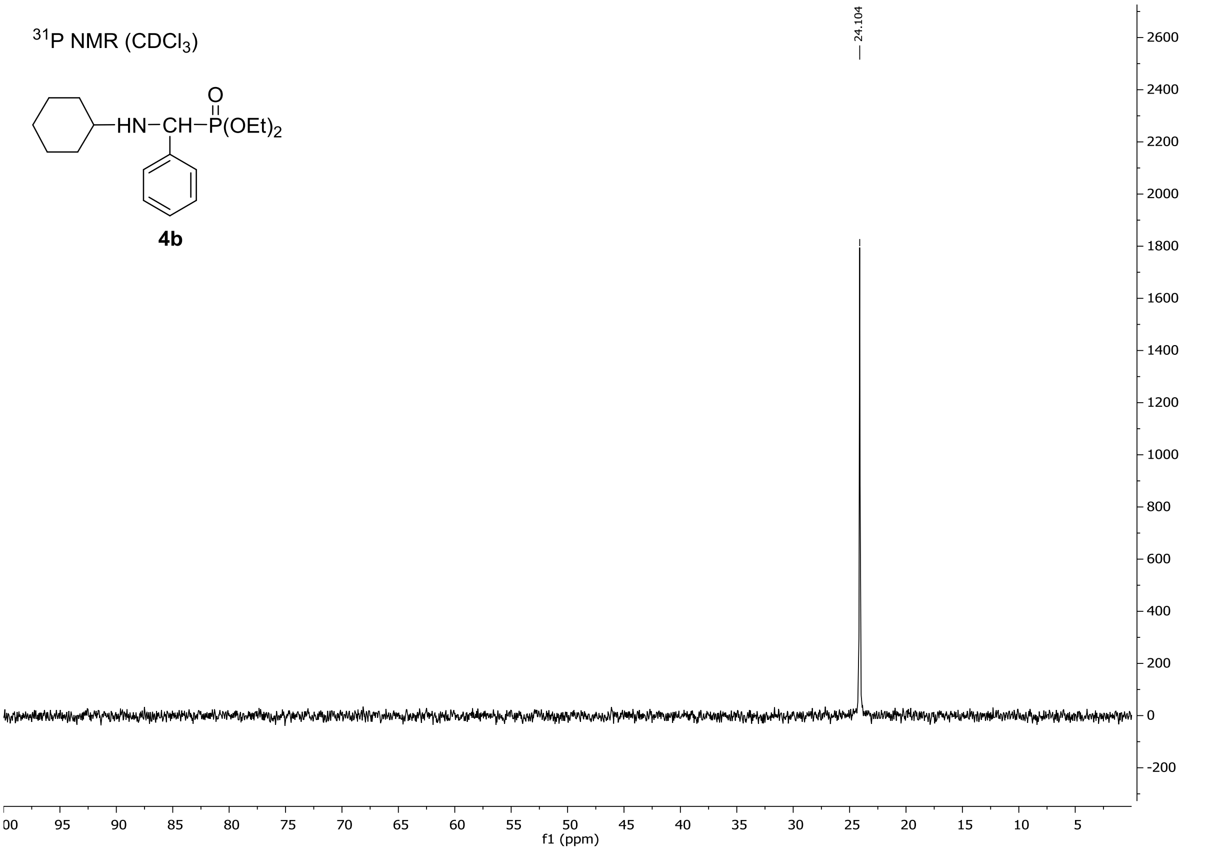
^{31}P NMR ($CDCl_3$)
HN−CH−P(OEt)$_2$
O
4b
24.104
f1 (ppm)

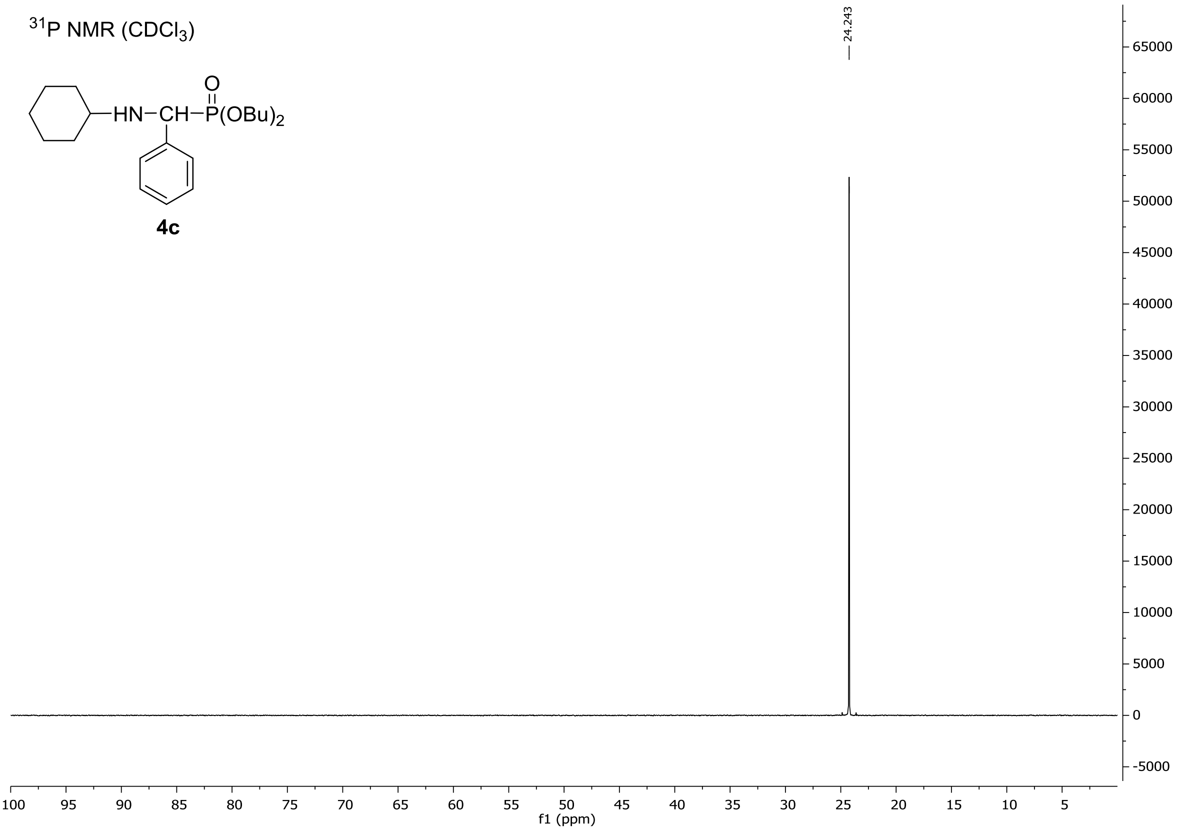

^{31}P NMR (CDCl$_3$)
HN−CH−P(OBu)$_2$
O
4c
24.243
f1 (ppm)

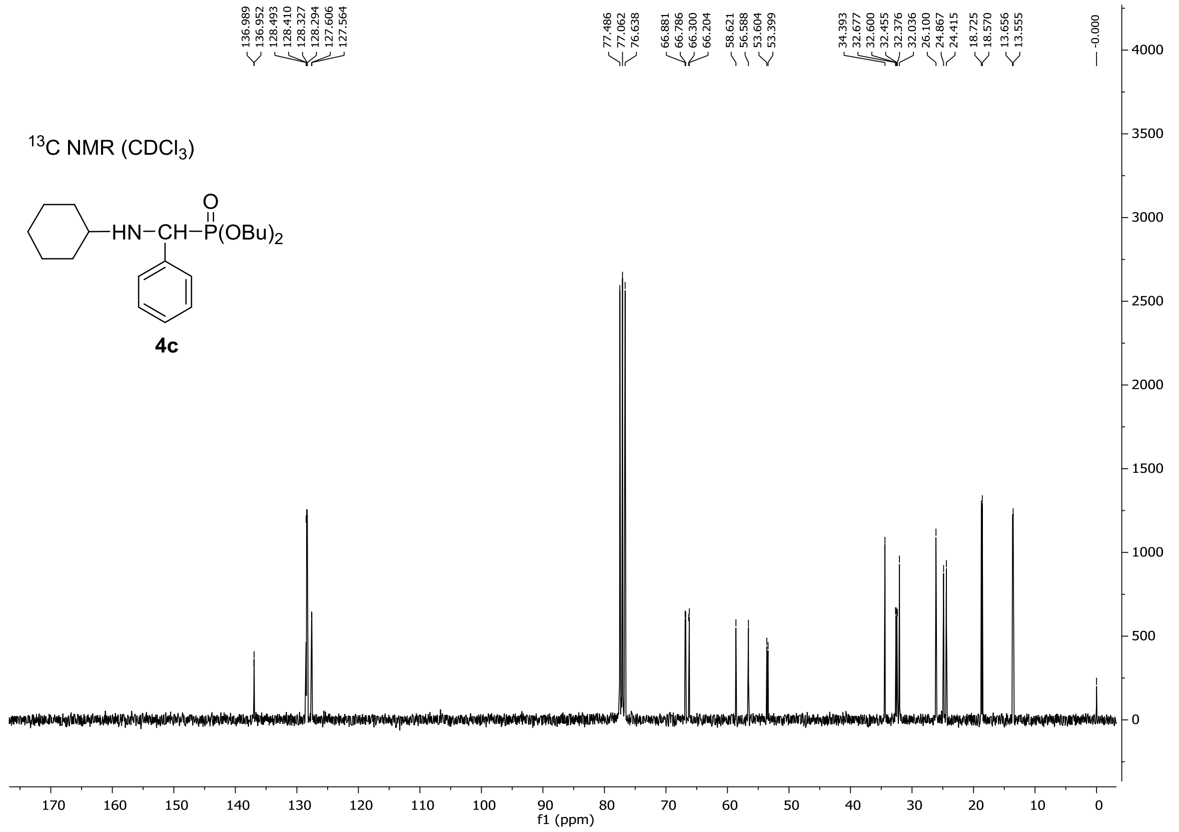

^{13}C NMR (CDCl$_3$)
HN−CH−P(OBu)$_2$
O
4c
136.989
136.952
128.493
128.410
128.327
128.294
127.606
127.564
77.486
77.062
76.638
66.881
66.786
66.300
66.204
58.621
56.588
53.604
53.399
34.393
32.677
32.600
32.455
32.376
32.036
26.100
24.867
24.415
18.725
18.570
13.656
13.555
-0.000
f1 (ppm)

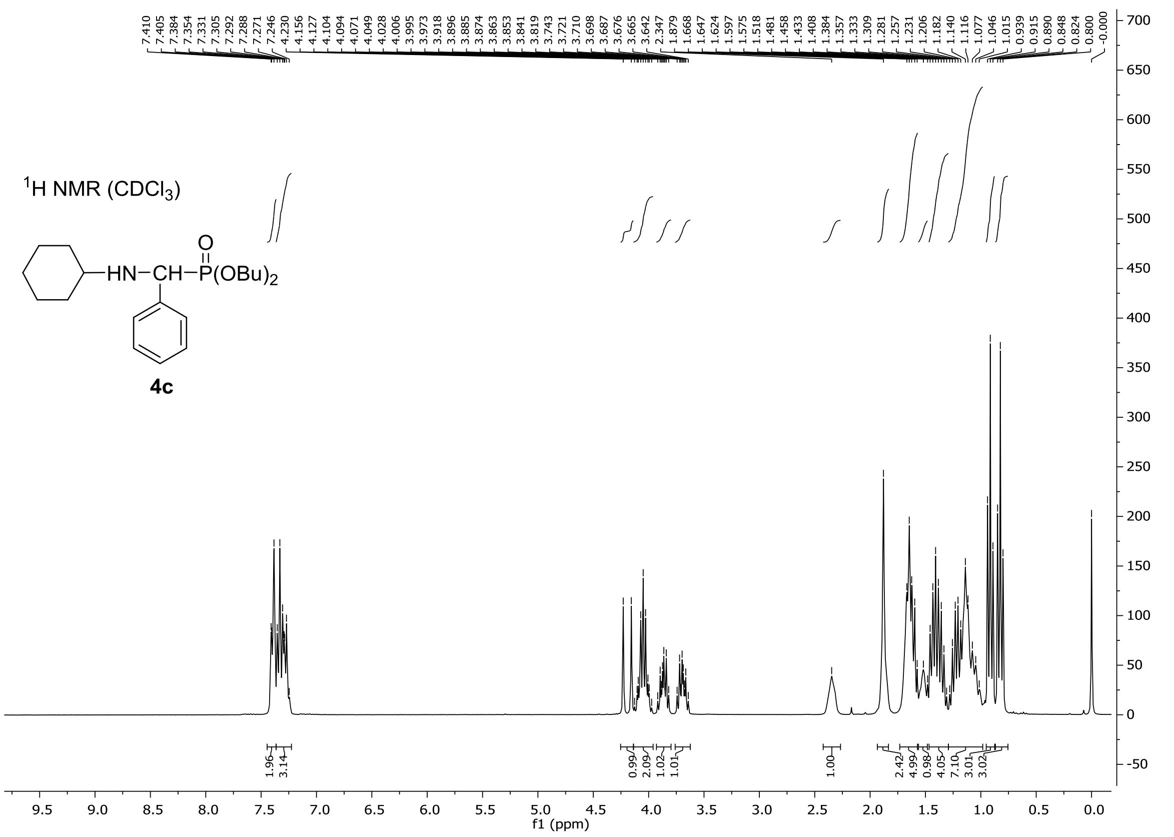
7.410 7.405 7.384 7.354 7.331 7.305 7.292 7.288 7.271 7.246 4.230 4.156 4.127 4.104 4.094 4.071 4.049 4.028 4.006 3.995 3.973 3.918 3.896 3.885 3.874 3.863 3.853 3.841 3.819 3.743 3.721 3.710 3.698 3.687 3.676 3.665 3.642 2.347 1.879 1.668 1.647 1.624 1.597 1.575 1.518 1.481 1.458 1.433 1.408 1.384 1.357 1.333 1.309 1.281 1.257 1.231 1.206 1.182 1.140 1.116 1.077 1.046 1.015 0.939 0.915 0.890 0.848 0.824 0.800 -0.000
1H NMR (CDCl3)
HN–CH–P(OBu)2
O
4c
1.96
3.14
0.99
2.09
1.02
1.01
1.00
2.42
4.99
0.98
4.05
7.10
3.01
3.02
f1 (ppm)

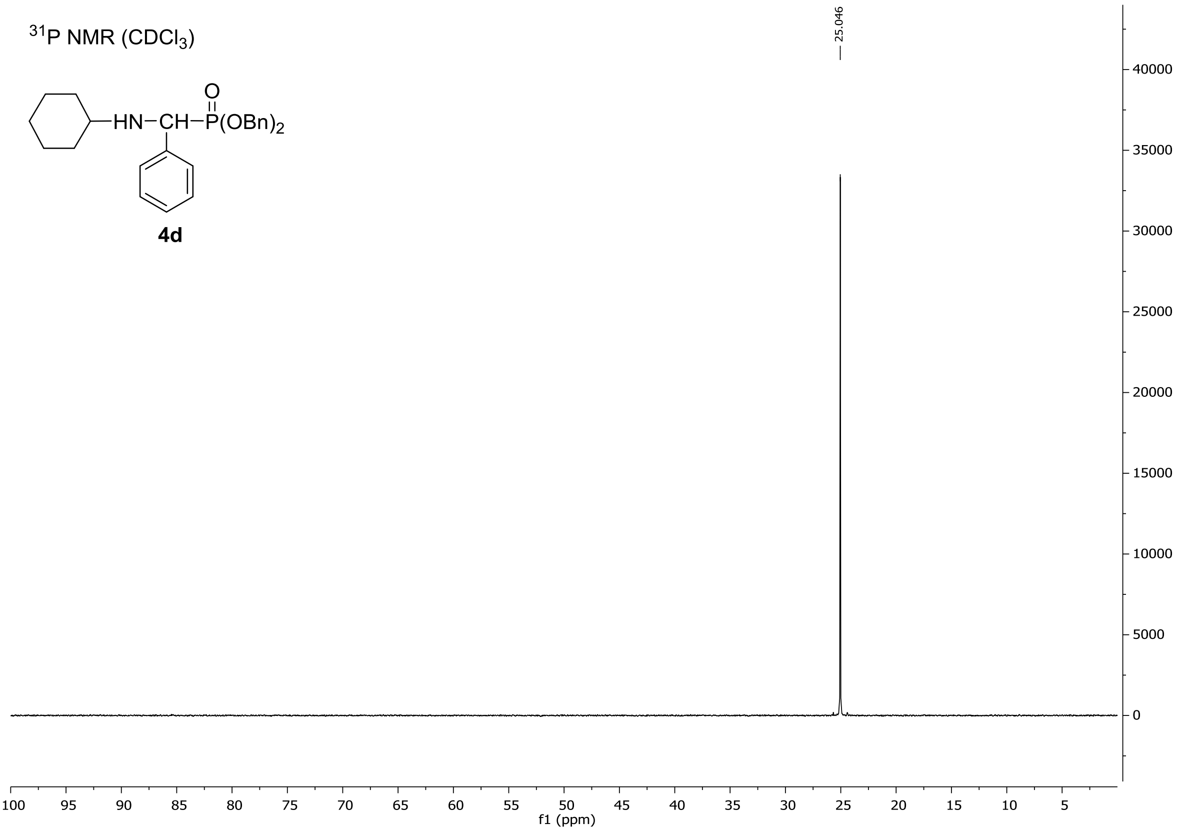
25.046
31P NMR (CDCl3)
HN–CH–P(OBn)2
O
4d
f1 (ppm)

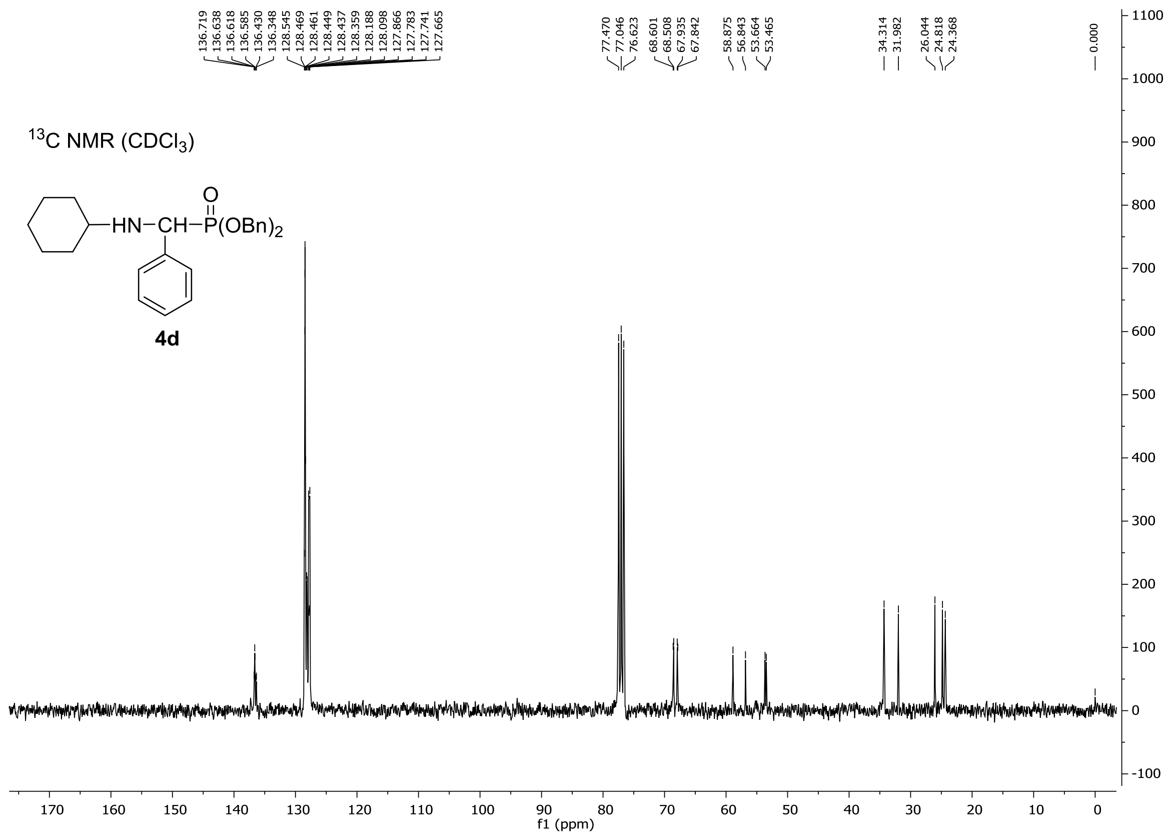

^{13}C NMR ($CDCl_3$)
HN–CH–P(OBn)$_2$
O
4d
f1 (ppm)

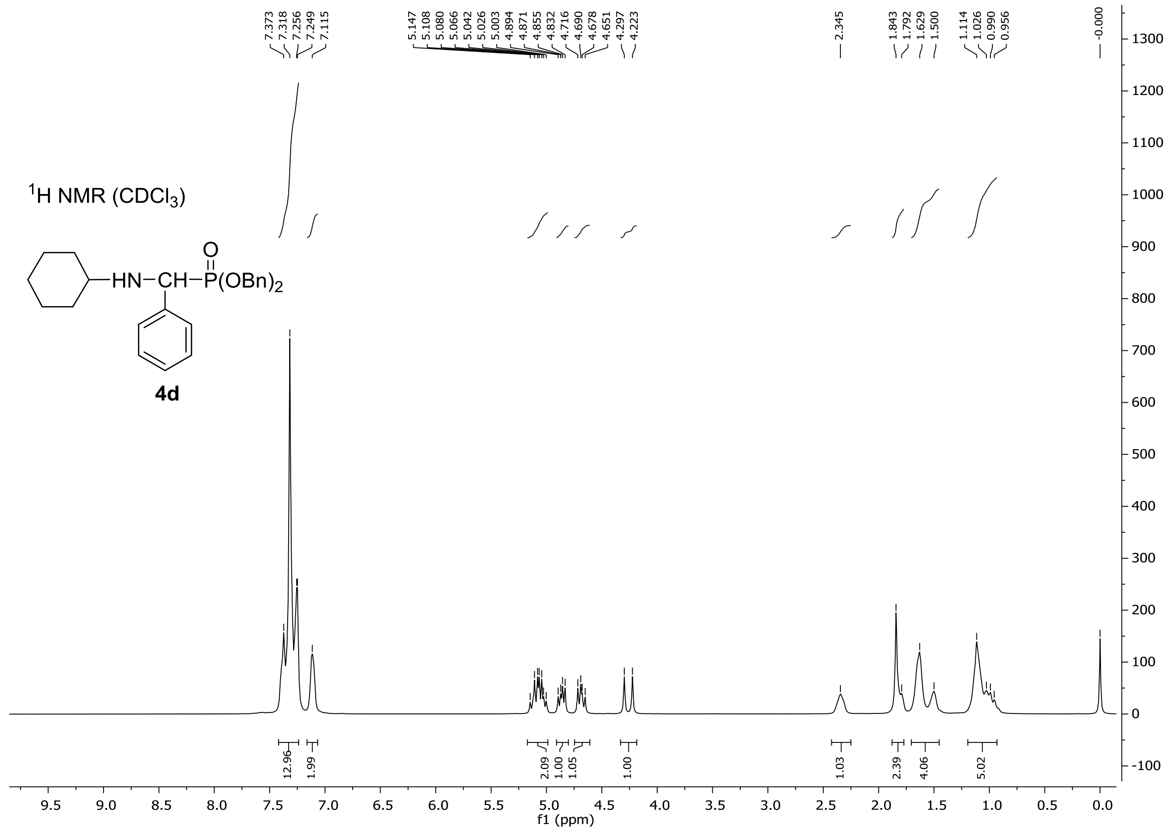

^{1}H NMR ($CDCl_3$)
HN–CH–P(OBn)$_2$
O
4d
f1 (ppm)

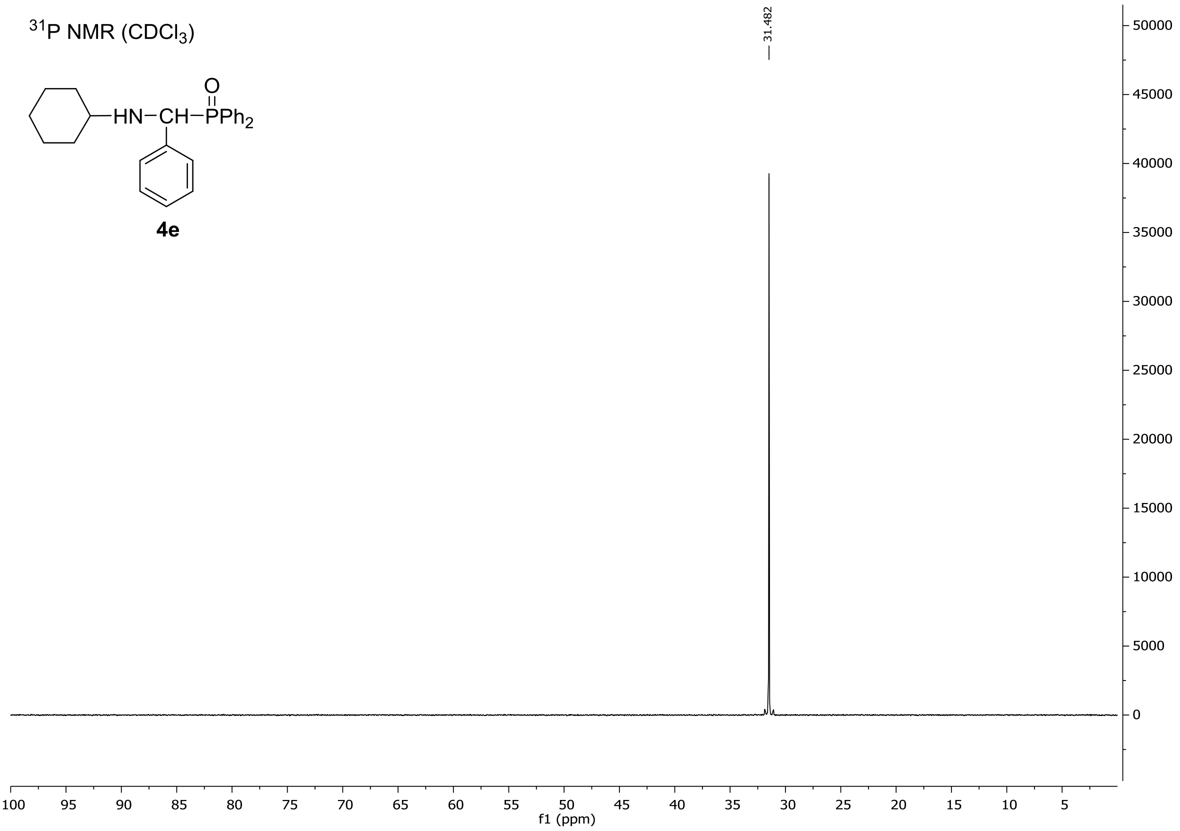

31P NMR (CDCl3)
HN–CH–PPh2
O
4e
31.482
f1 (ppm)

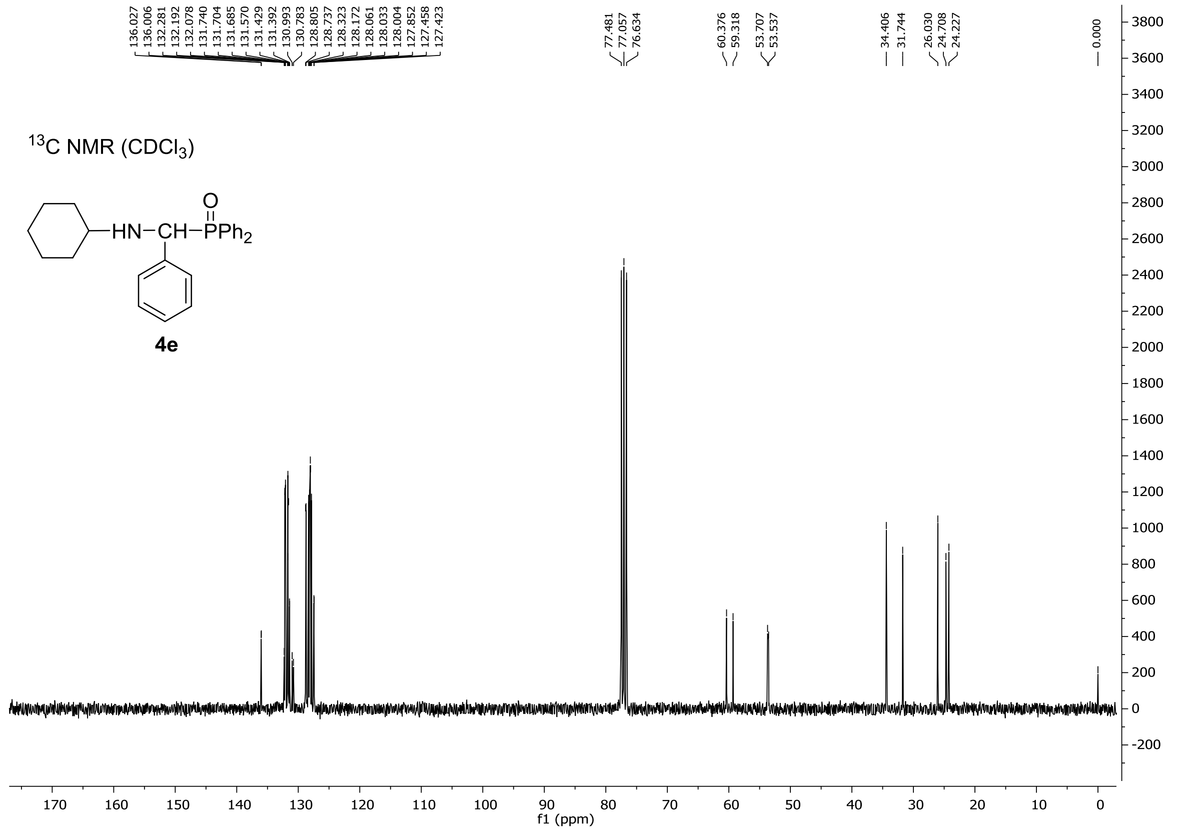

13C NMR (CDCl3)
HN–CH–PPh2
O
4e
136.027
136.006
132.281
132.192
132.078
131.740
131.704
131.685
131.570
131.429
131.392
130.993
130.783
128.805
128.737
128.323
128.172
128.061
128.033
128.004
127.852
127.458
127.423
77.481
77.057
76.634
60.376
59.318
53.707
53.537
34.406
31.744
26.030
24.708
24.227
0.000
f1 (ppm)

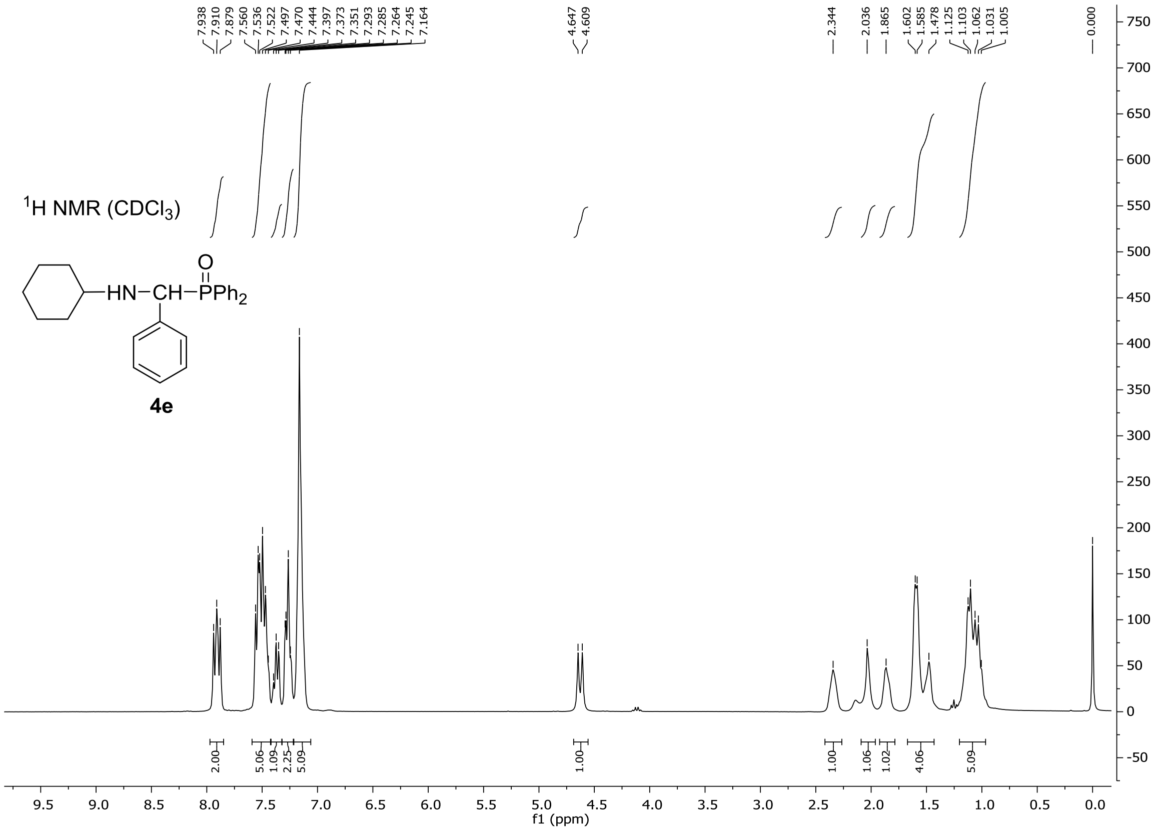

^1H NMR ($CDCl_3$)

4e

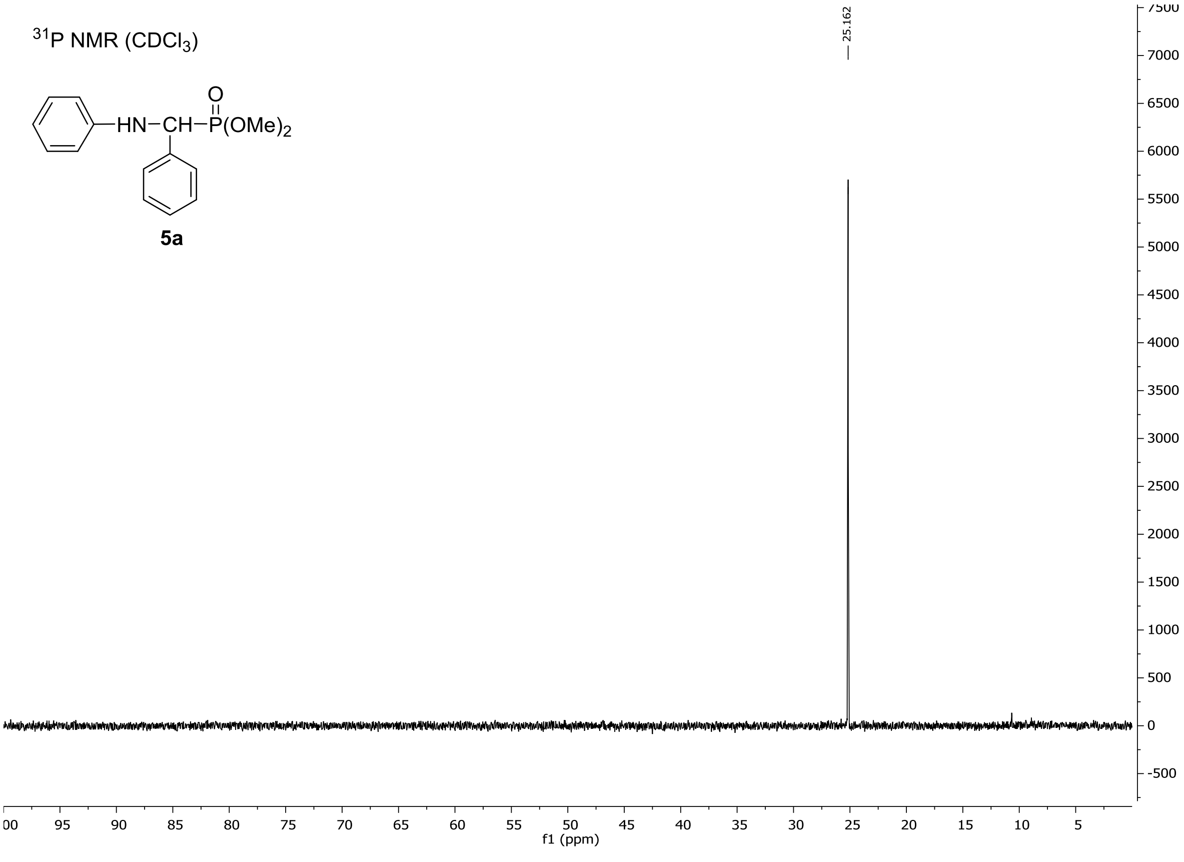

^{31}P NMR ($CDCl_3$)

5a

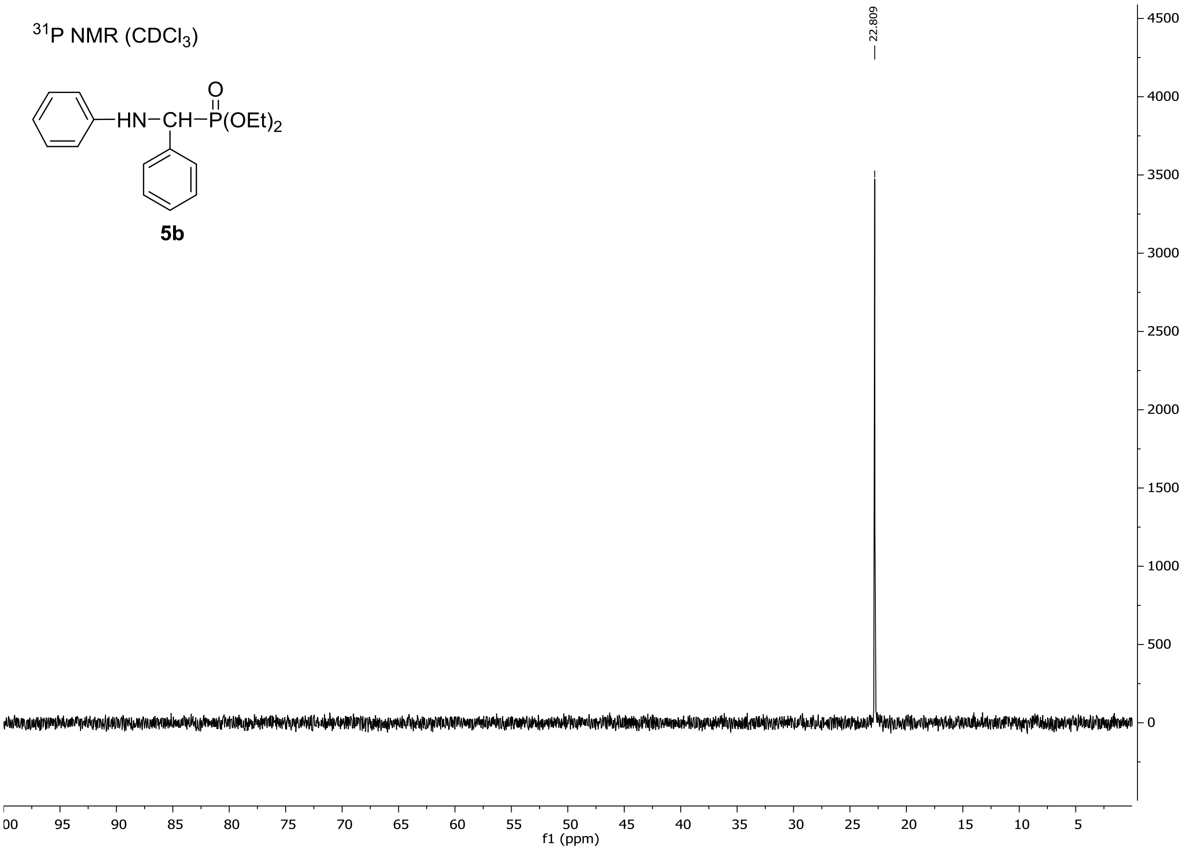

31P NMR (CDCl3)
HN–CH–P(OEt)2
O
5b
22.809
f1 (ppm)

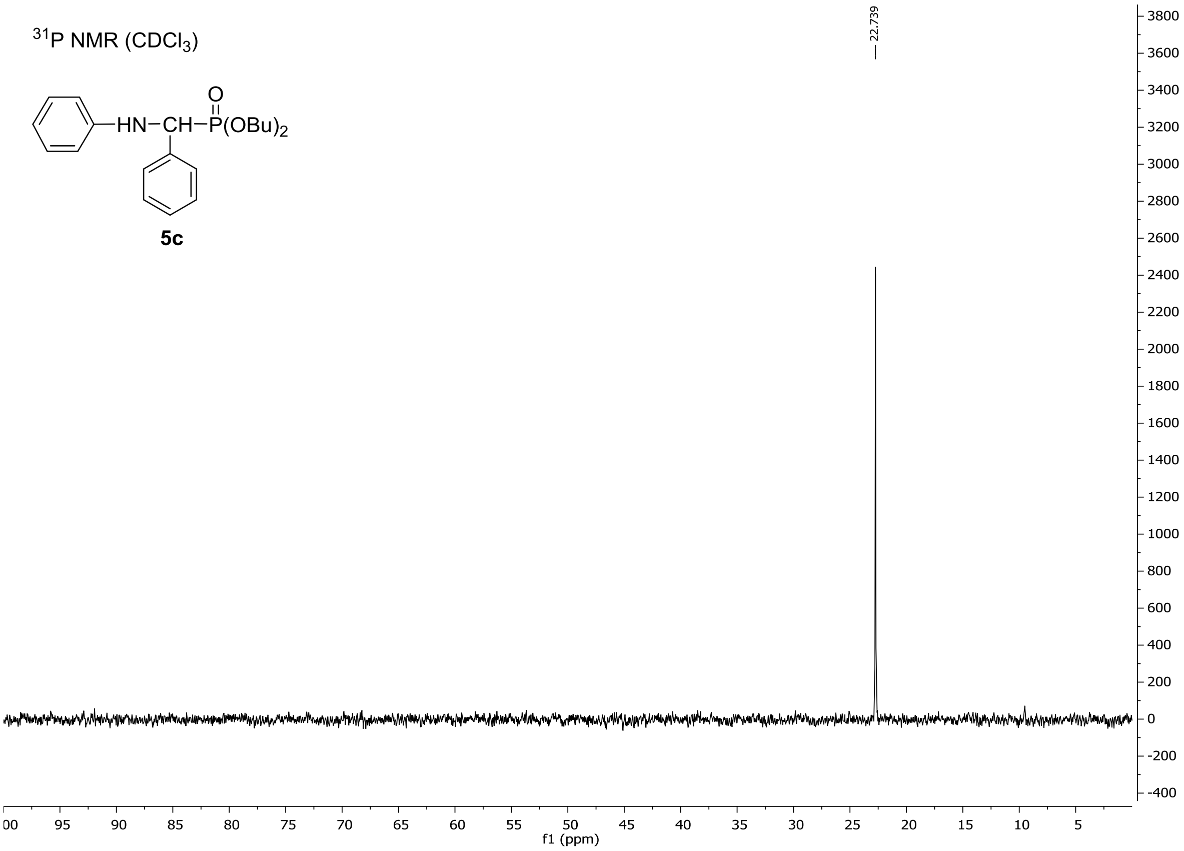

31P NMR (CDCl3)
HN–CH–P(OBu)2
O
5c
22.739
f1 (ppm)

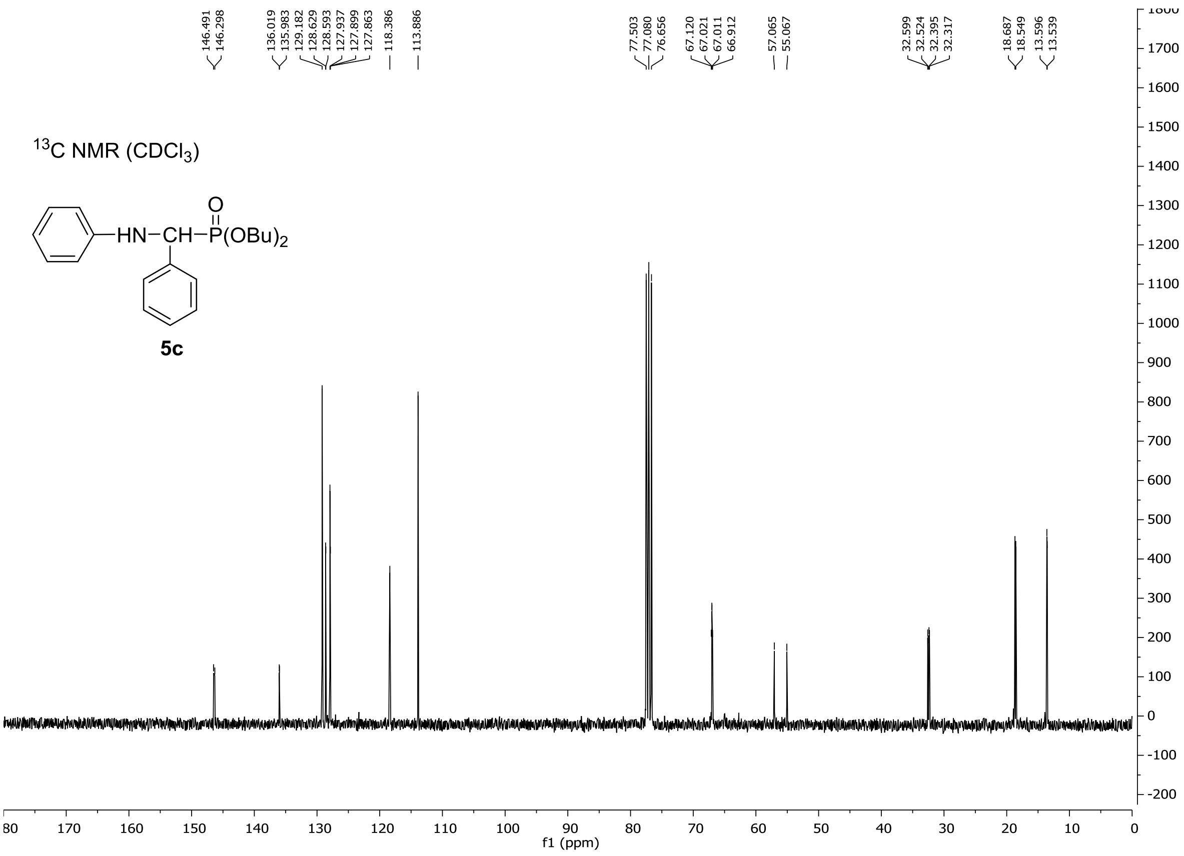

^{13}C NMR (CDCl$_3$)
HN−CH−P(OBu)$_2$
O
5c
f1 (ppm)

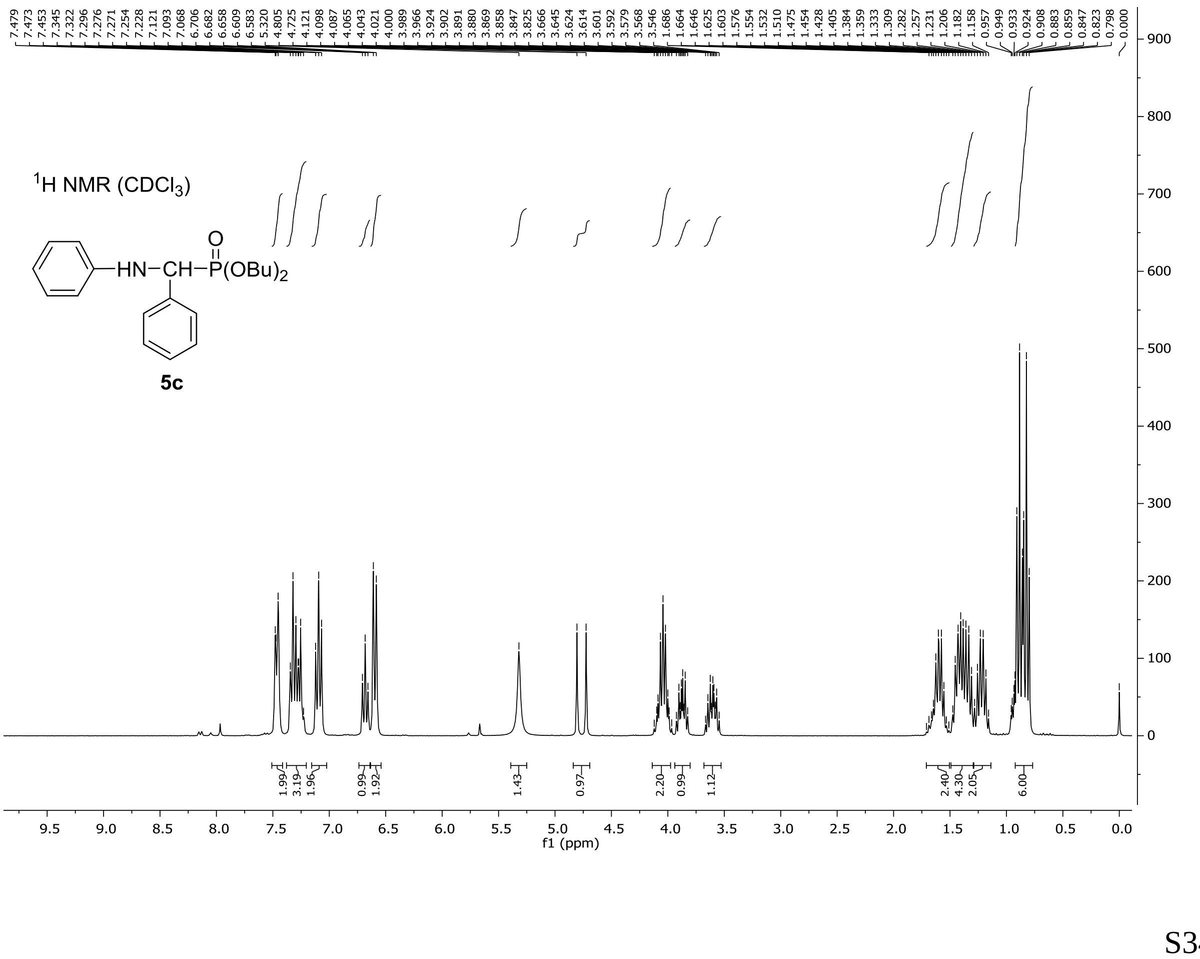

^{1}H NMR (CDCl$_3$)
HN−CH−P(OBu)$_2$
O
5c
f1 (ppm)

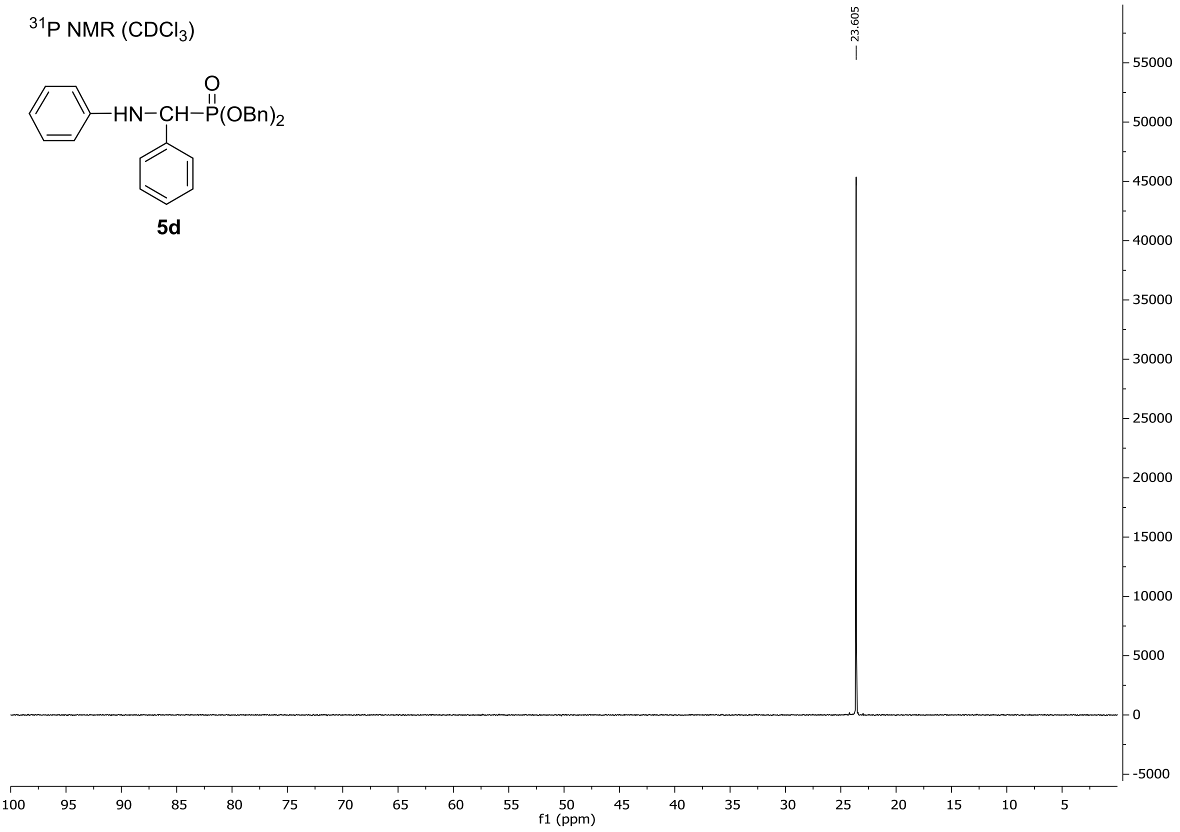

31P NMR (CDCl3)
HN−CH−P(OBn)2
O
5d
23.605
f1 (ppm)

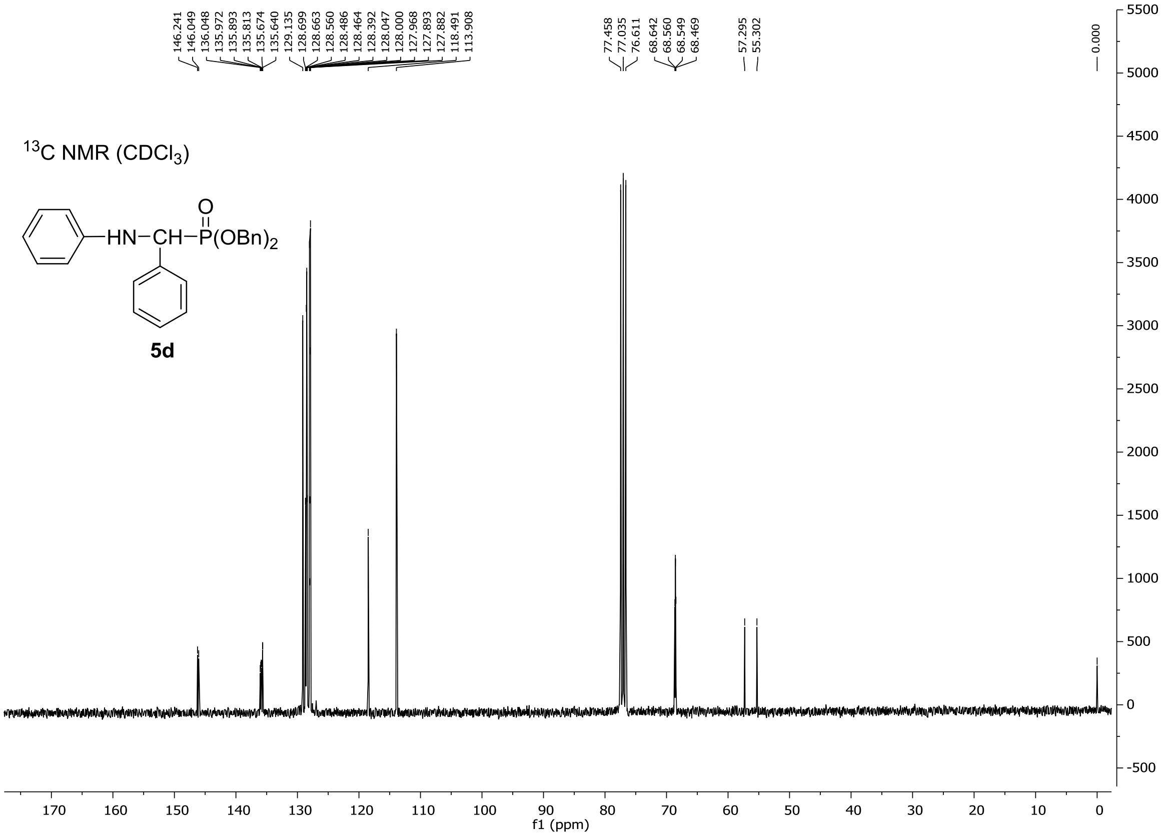

13C NMR (CDCl3)
HN−CH−P(OBn)2
O
5d
146.241
146.049
136.048
135.972
135.893
135.813
135.674
135.640
129.135
128.699
128.663
128.560
128.486
128.464
128.392
128.047
128.000
127.968
127.893
127.882
118.491
113.908
77.458
77.035
76.611
68.642
68.560
68.549
68.469
57.295
55.302
0.000
f1 (ppm)

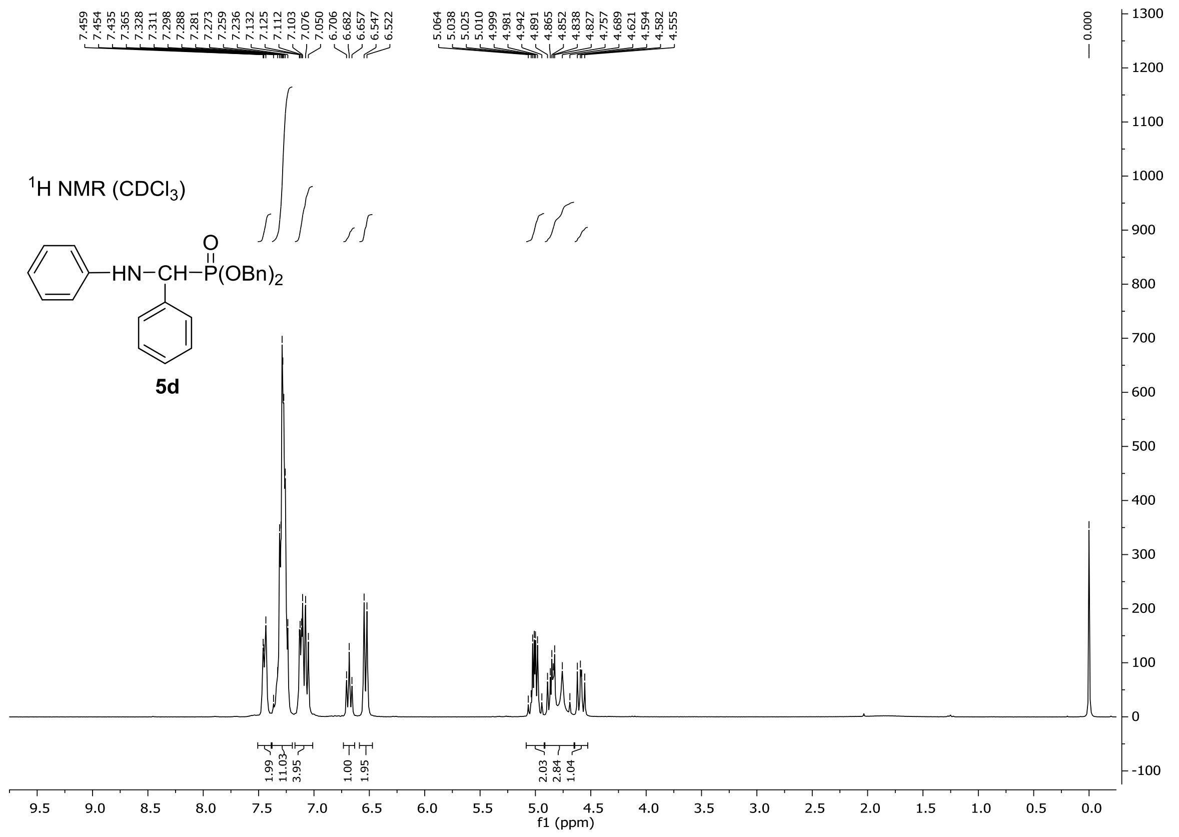

1H NMR (CDCl3)
HN−CH−P(OBn)2
O
5d
f1 (ppm)

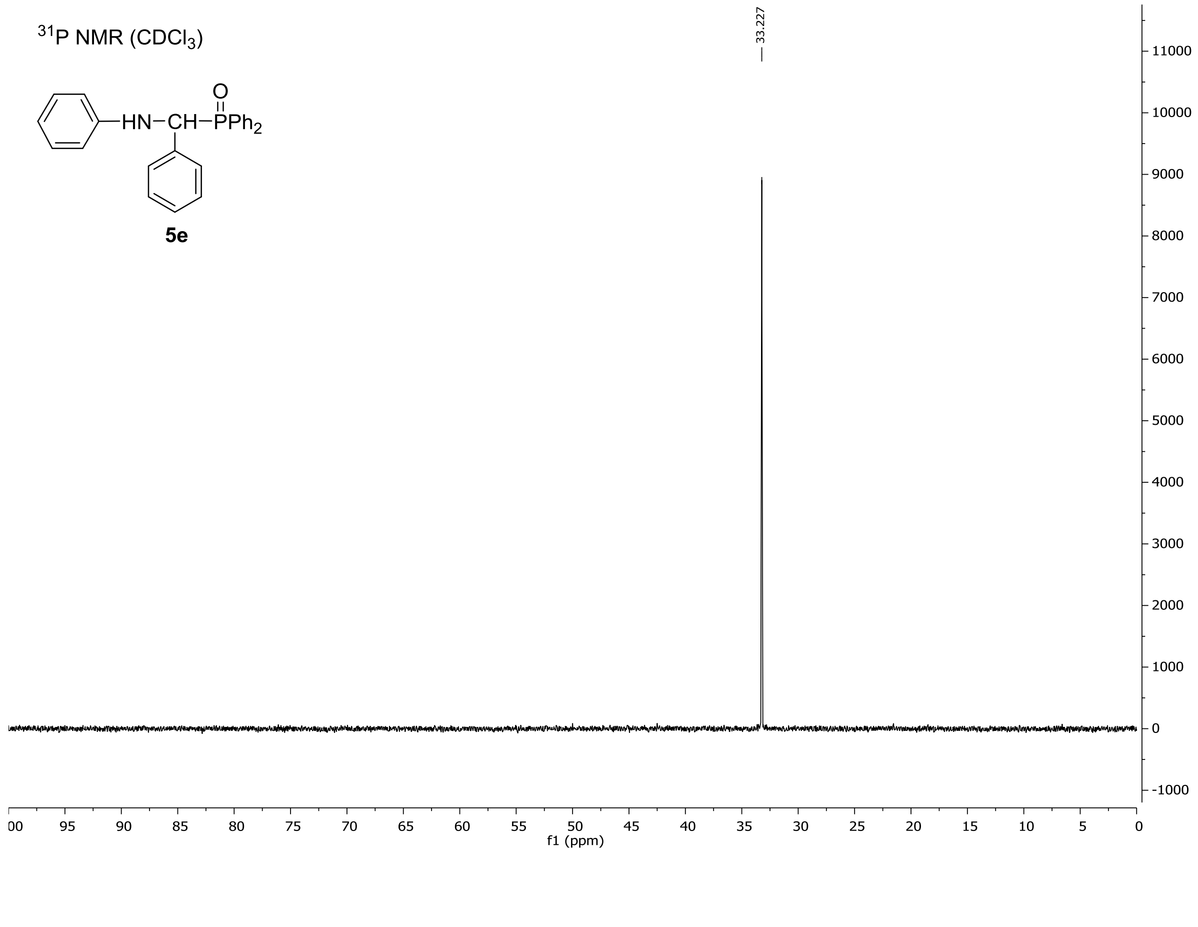

31P NMR (CDCl3)
HN−CH−PPh2
O
5e
33.227
f1 (ppm)

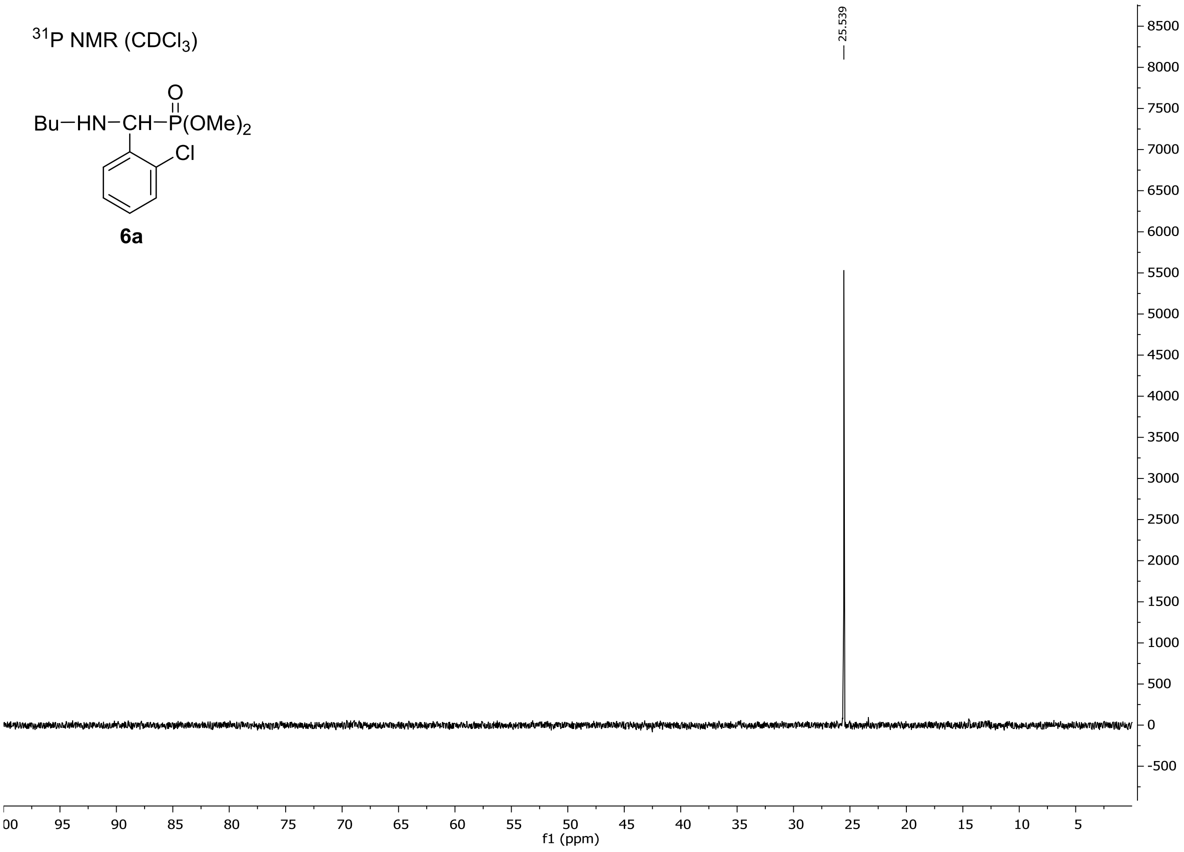
^{31}P NMR ($CDCl_3$)
Bu−HN−CH−P(OMe)2
O
Cl
6a
25.539
f1 (ppm)

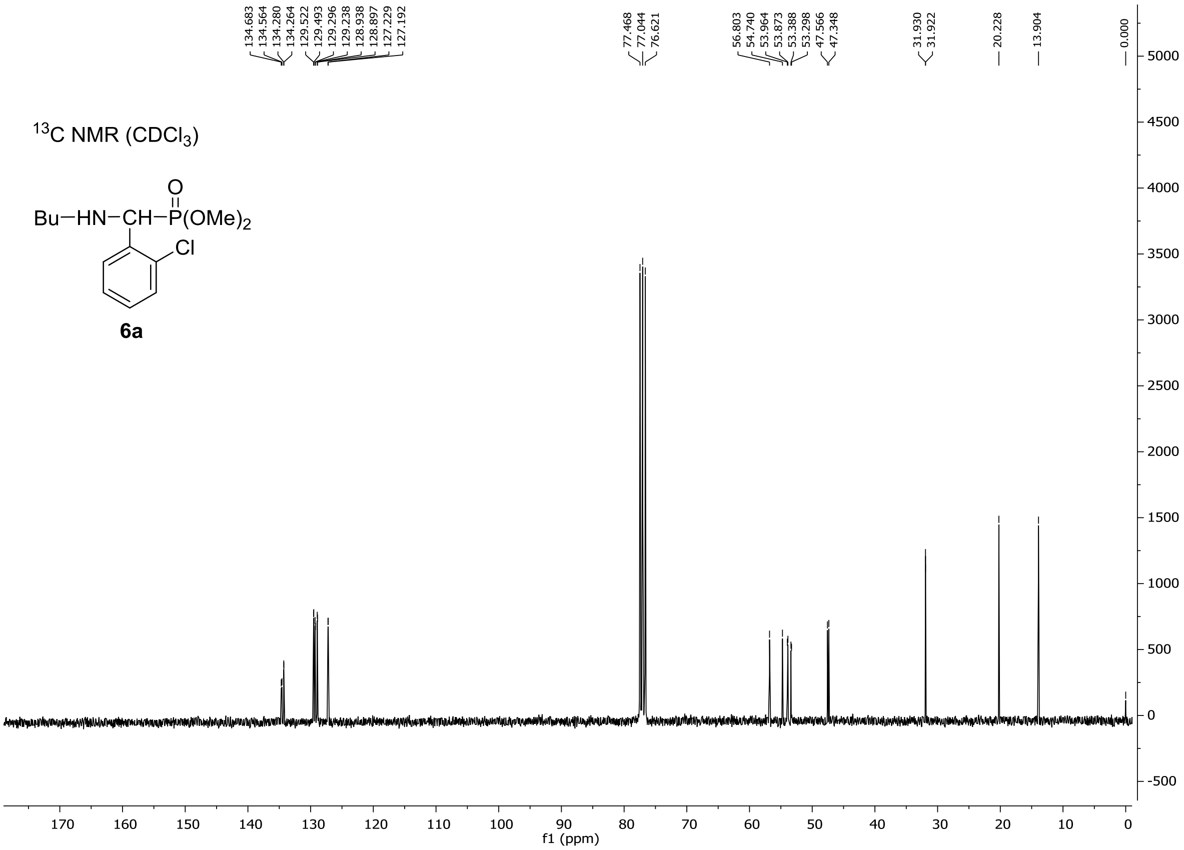
^{13}C NMR ($CDCl_3$)
Bu−HN−CH−P(OMe)2
O
Cl
6a
134.683
134.564
134.280
134.264
129.522
129.493
129.296
129.238
128.938
128.897
127.229
127.192
77.468
77.044
76.621
56.803
54.740
53.964
53.873
53.388
53.298
47.566
47.348
31.930
31.922
20.228
13.904
0.000
f1 (ppm)

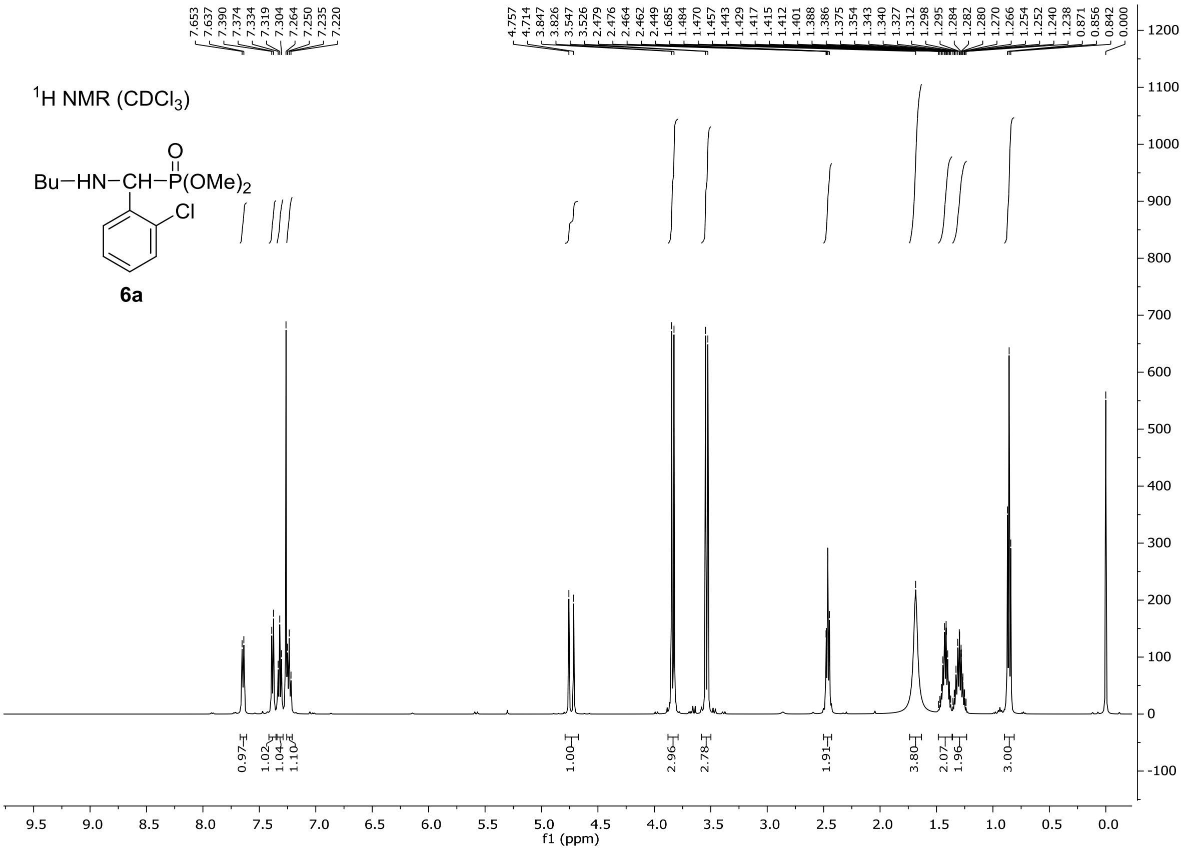
1H NMR (CDCl3)
Bu−HN−CH−P(OMe)2
O
Cl
6a
f1 (ppm)

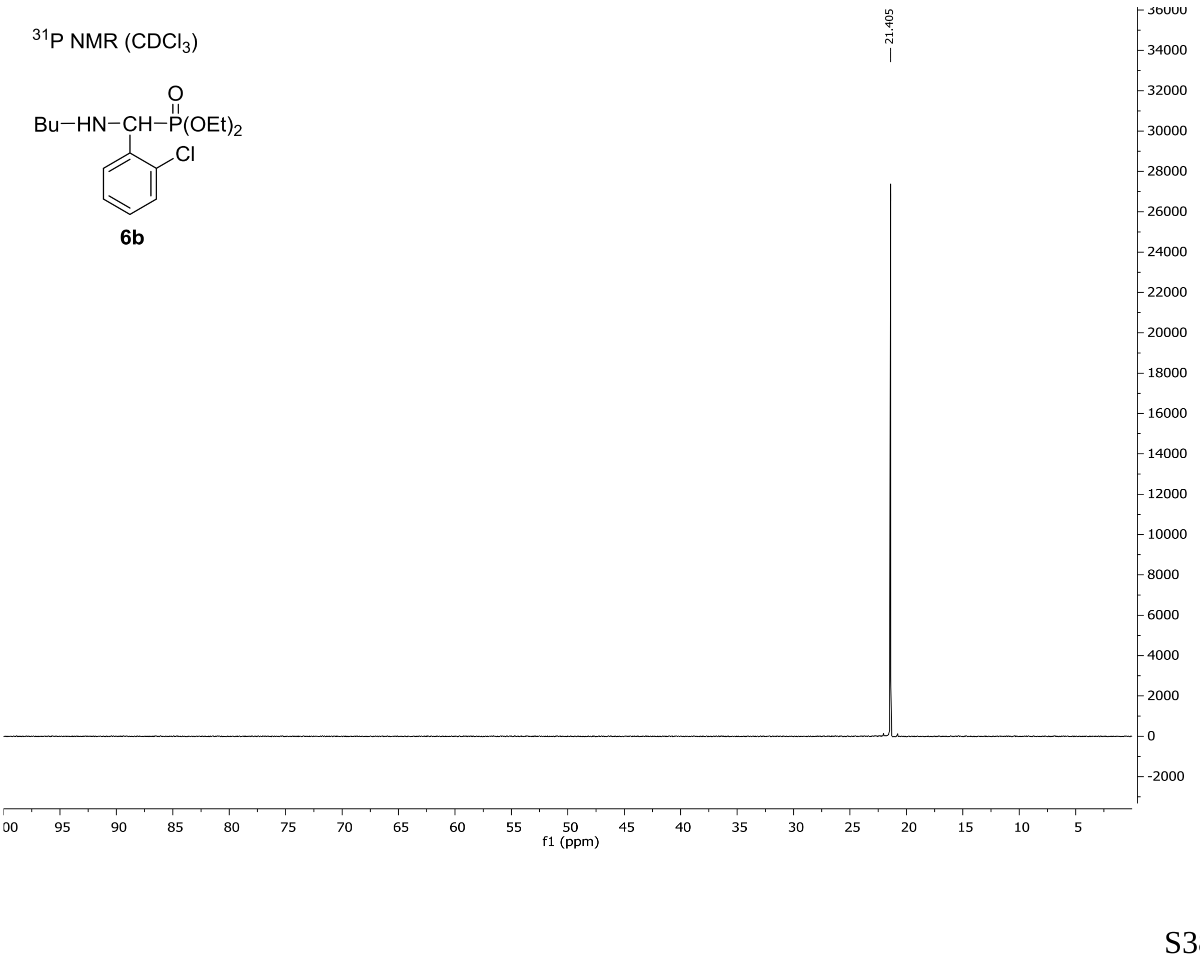
31P NMR (CDCl3)
Bu−HN−CH−P(OEt)2
O
Cl
6b
21.405
f1 (ppm)

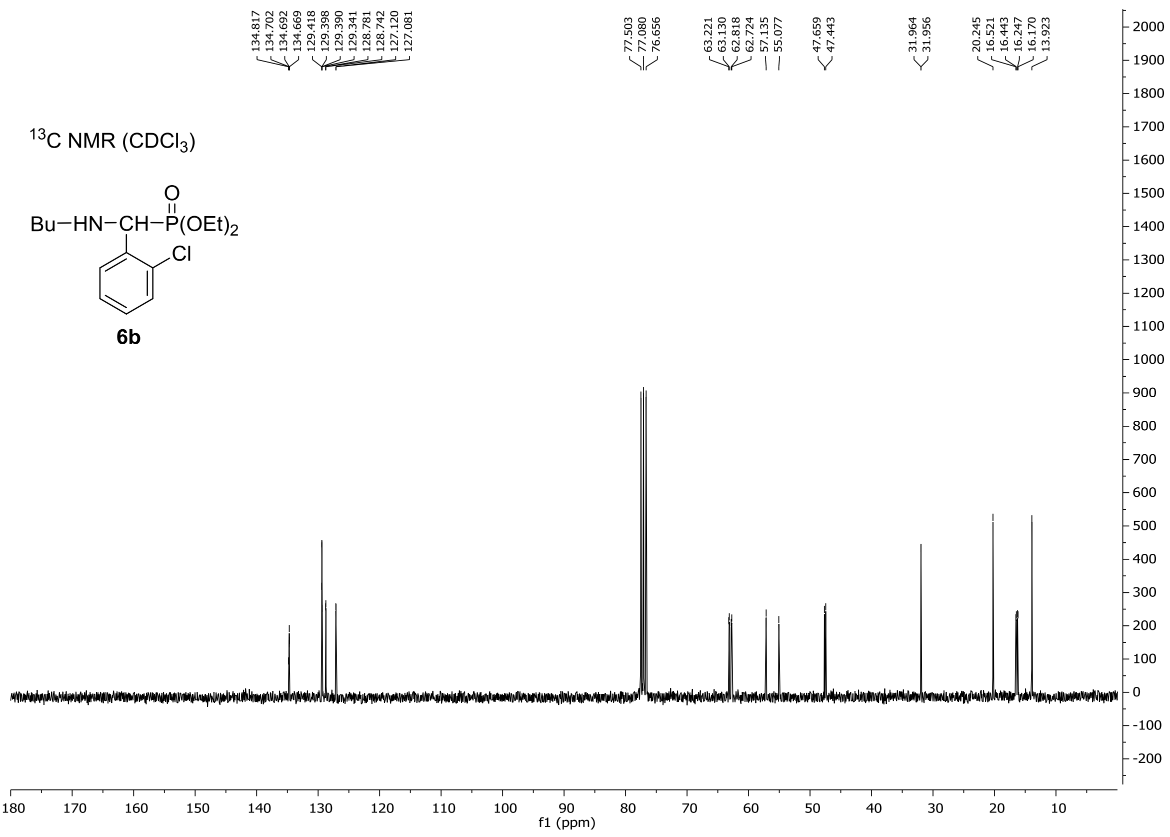

^{13}C NMR (CDCl$_3$)
Bu−HN−CH−P(OEt)$_2$
O
Cl
6b
f1 (ppm)

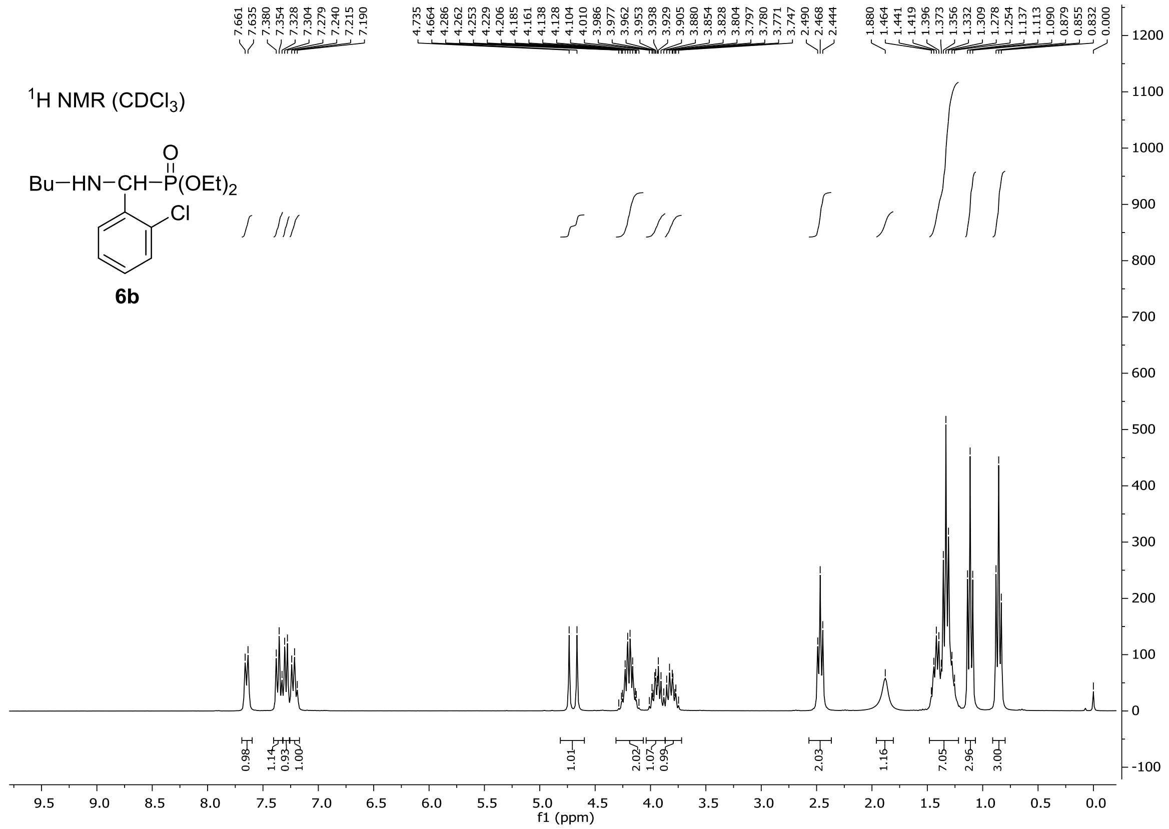

^{1}H NMR (CDCl$_3$)
Bu−HN−CH−P(OEt)$_2$
O
Cl
6b
f1 (ppm)

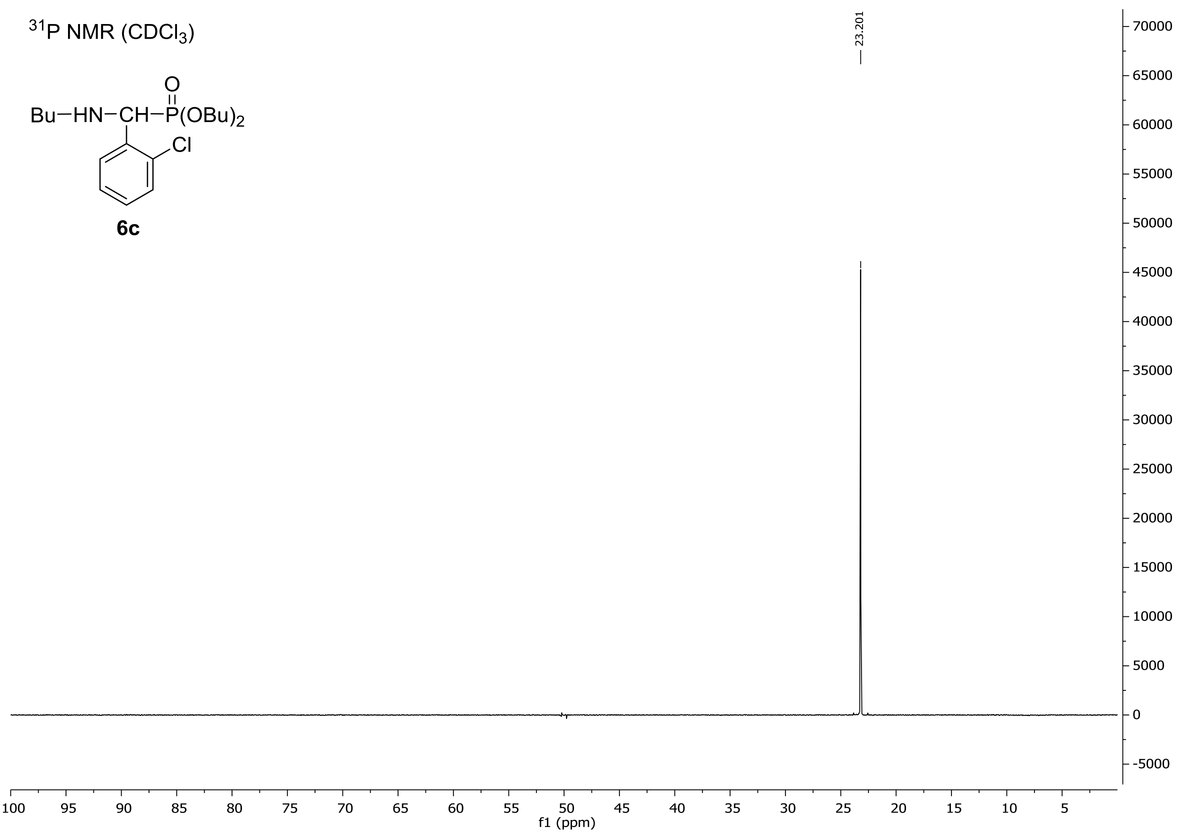
^{31}P NMR ($CDCl_3$)
Bu−HN−CH−P(OBu)2
O
Cl
6c
23.201
f1 (ppm)

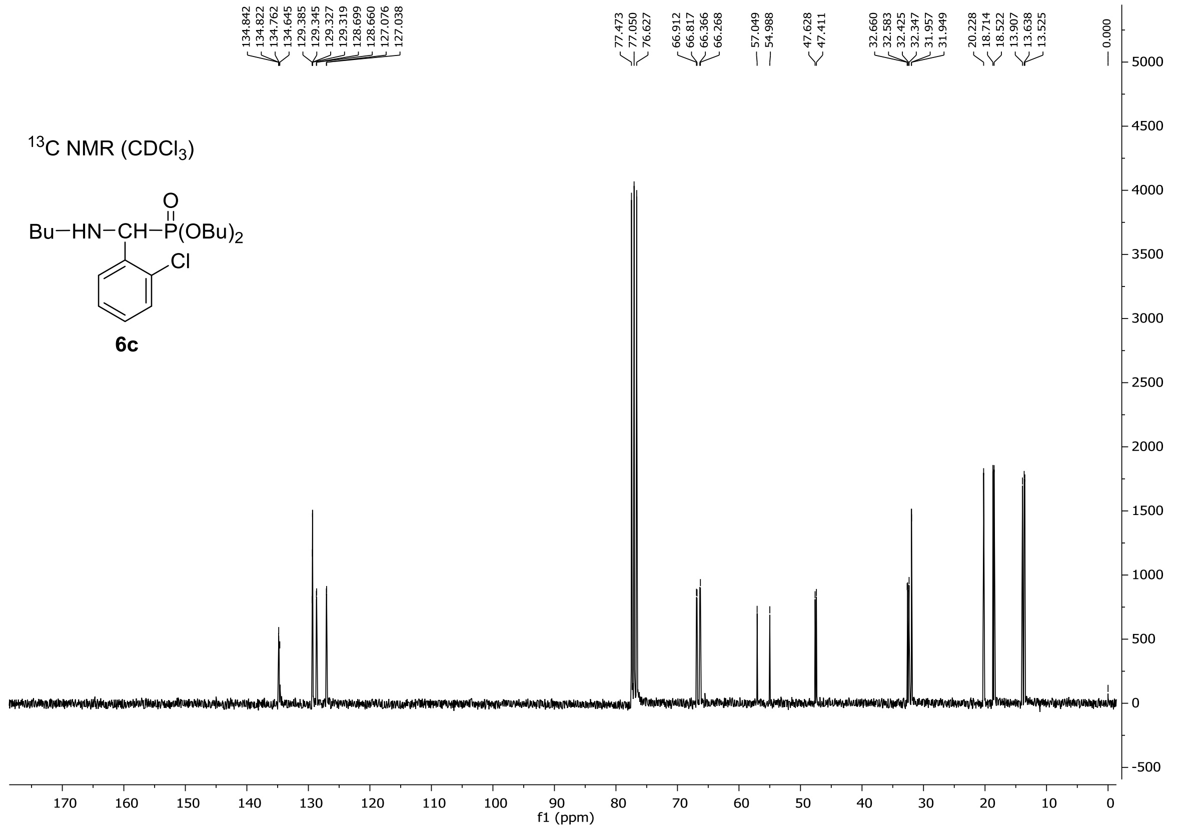
^{13}C NMR ($CDCl_3$)
Bu−HN−CH−P(OBu)2
O
Cl
6c
134.842
134.822
134.762
134.645
129.385
129.345
129.327
129.319
128.699
128.660
127.076
127.038
77.473
77.050
76.627
66.912
66.817
66.366
66.268
57.049
54.988
47.628
47.411
32.660
32.583
32.425
32.347
31.957
31.949
20.228
18.714
18.522
13.907
13.638
13.525
0.000
f1 (ppm)

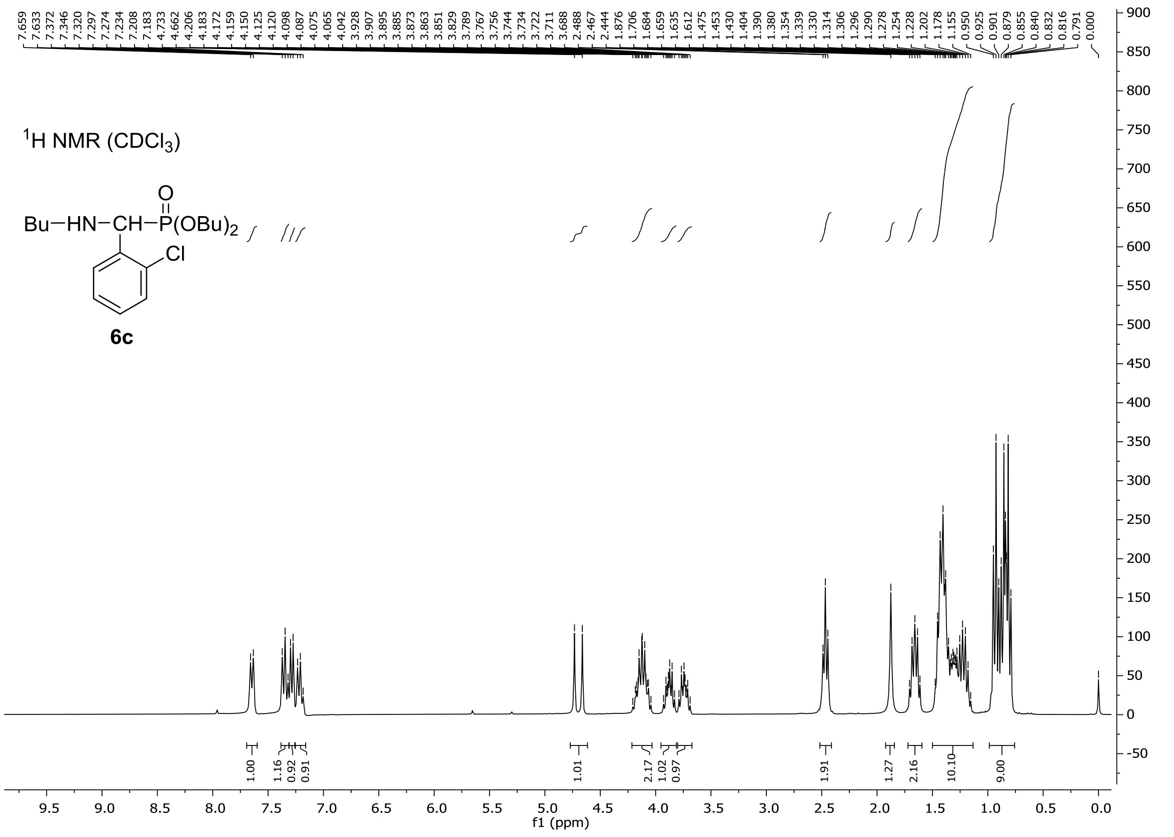
^{1}H NMR ($CDCl_3$)
Bu−HN−CH−P(OBu)$_2$
O
Cl
6c
1.00
1.16
0.92
0.91
1.01
2.17
1.02
0.97
1.91
1.27
2.16
10.10
9.00
f1 (ppm)

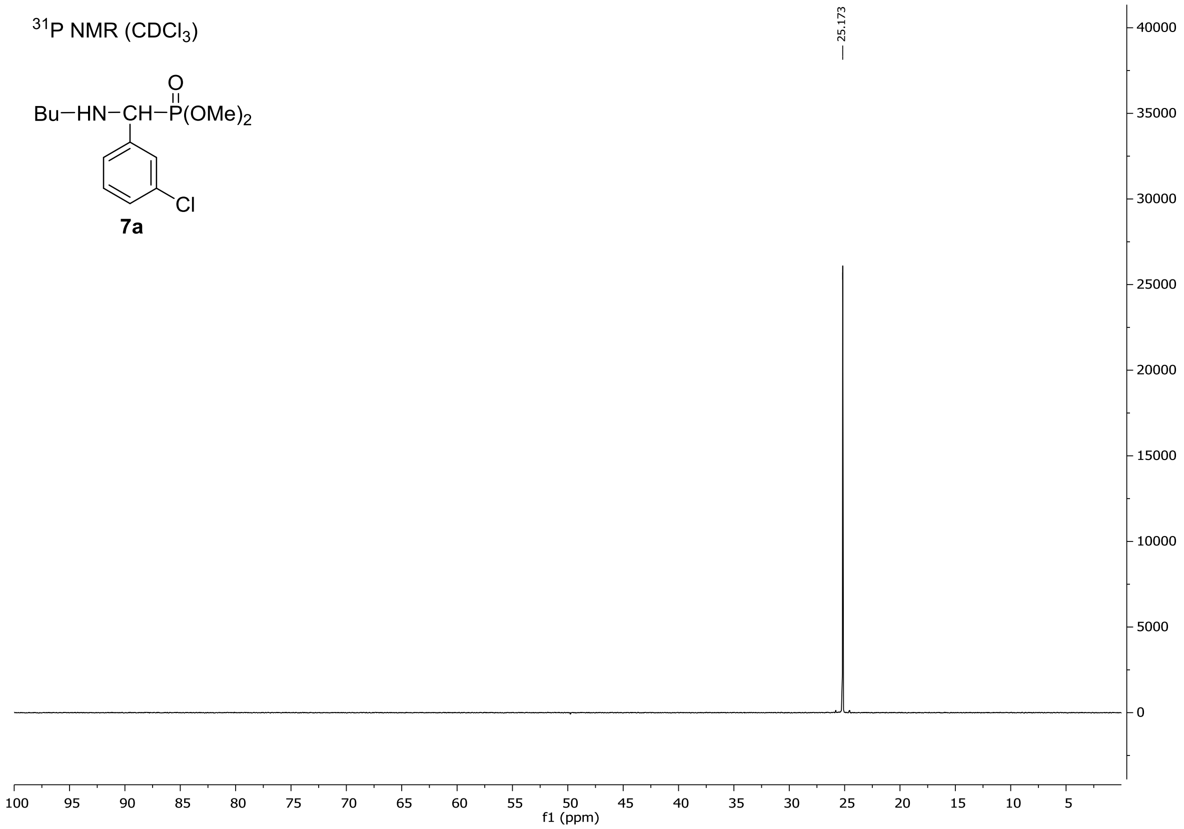
^{31}P NMR ($CDCl_3$)
Bu−HN−CH−P(OMe)$_2$
O
Cl
7a
25.173
f1 (ppm)

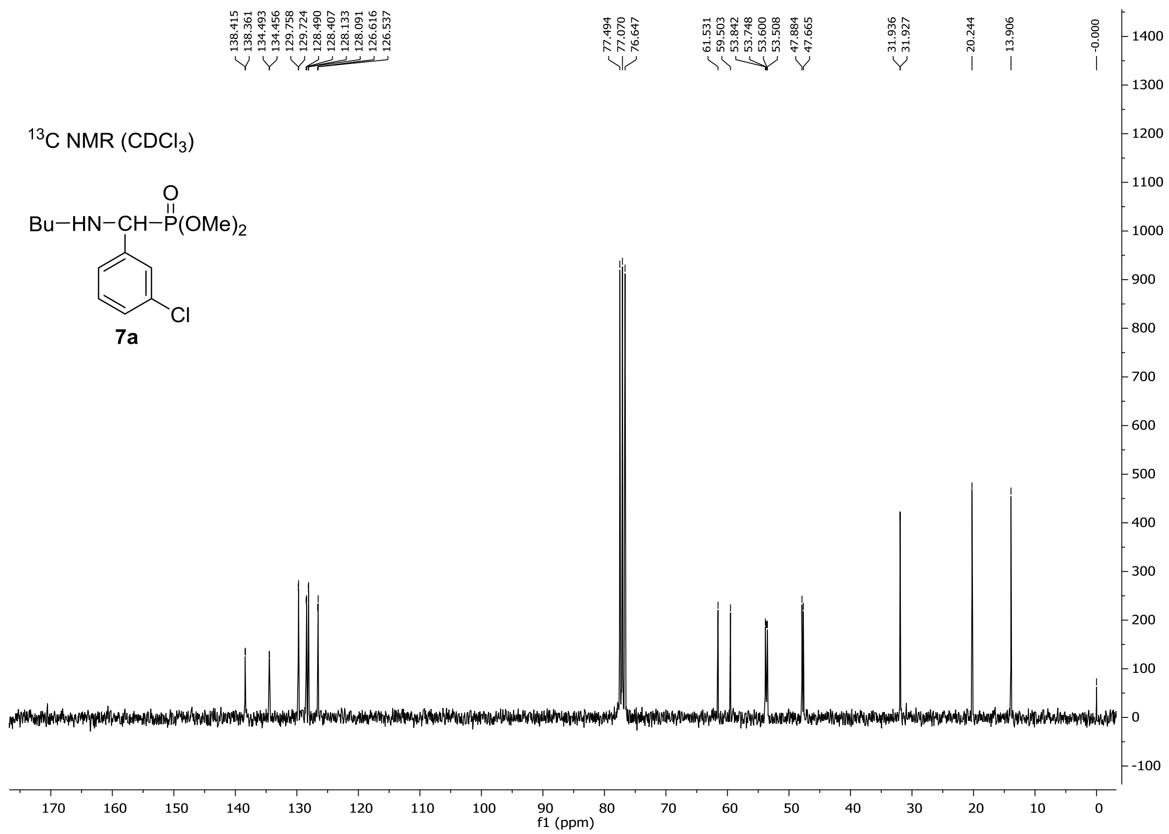
13C NMR (CDCl3)
Bu−HN−CH−P(OMe)2
O
Cl
7a
138.415 138.361 134.493 134.456 129.758 129.724 128.490 128.407 128.133 128.091 126.616 126.537
77.494 77.070 76.647
61.531 59.503 53.842 53.748 53.600 53.508 47.884 47.665
31.936 31.927
20.244
13.906
-0.000
f1 (ppm)

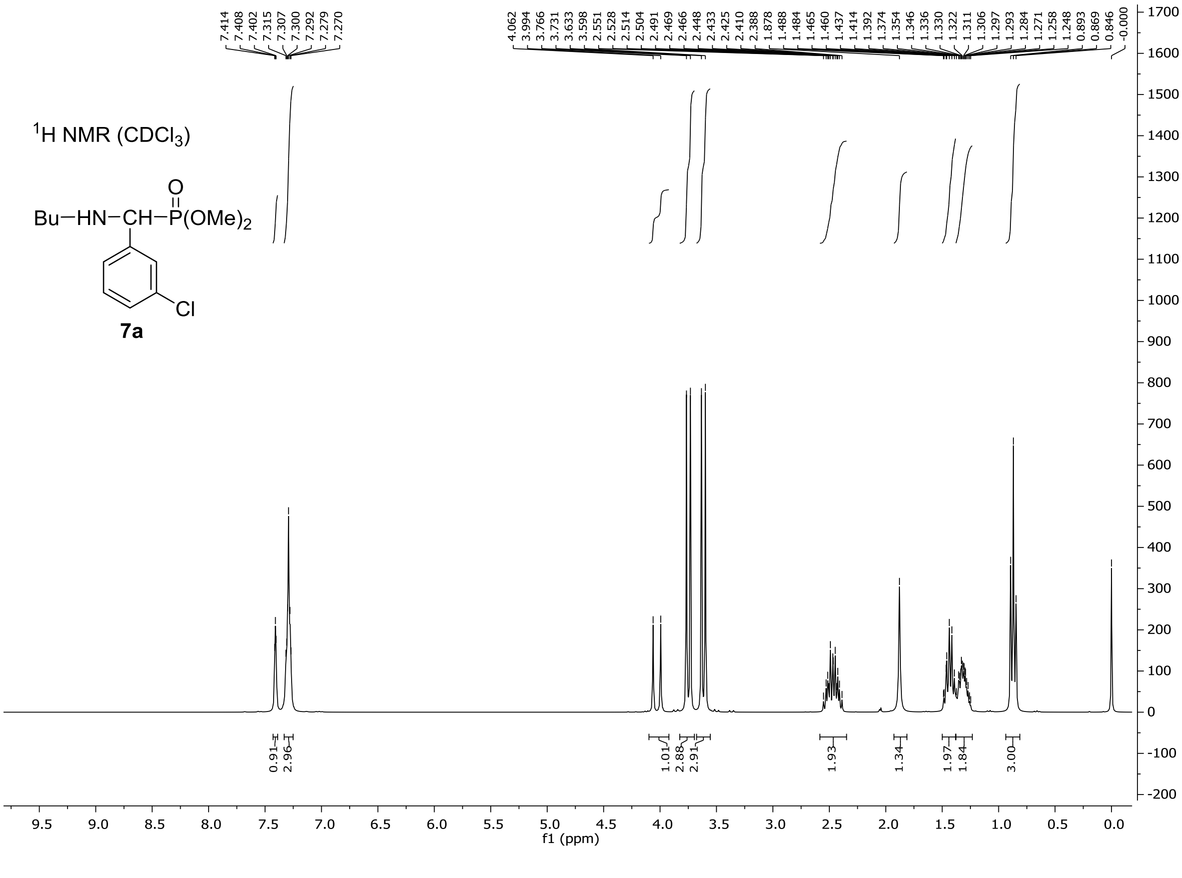
1H NMR (CDCl3)
Bu−HN−CH−P(OMe)2
O
Cl
7a
7.414 7.408 7.402 7.315 7.307 7.300 7.292 7.279 7.270
4.062 3.994 3.766 3.731 3.633 3.598 2.551 2.528 2.514 2.504 2.491 2.469 2.466 2.448 2.433 2.425 2.410 2.388 1.878 1.488 1.484 1.465 1.460 1.437 1.414 1.392 1.374 1.354 1.346 1.336 1.330 1.322 1.311 1.306 1.297 1.293 1.284 1.271 1.258 1.248 0.893 0.869 0.846 -0.000
0.91 2.96 1.01 2.88 2.91 1.93 1.34 1.97 1.84 3.00
f1 (ppm)

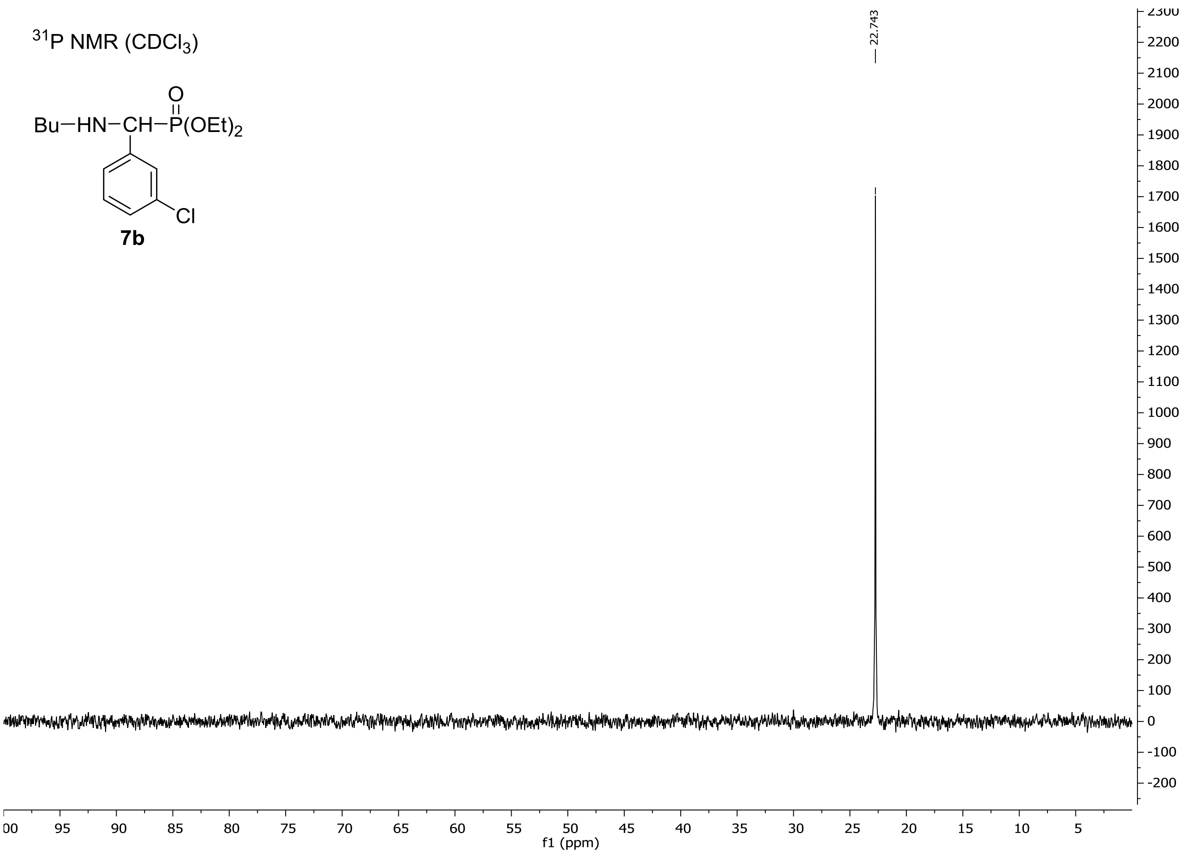
31P NMR (CDCl3)
Bu−HN−CH−P(OEt)2
O
Cl
7b
22.743
f1 (ppm)

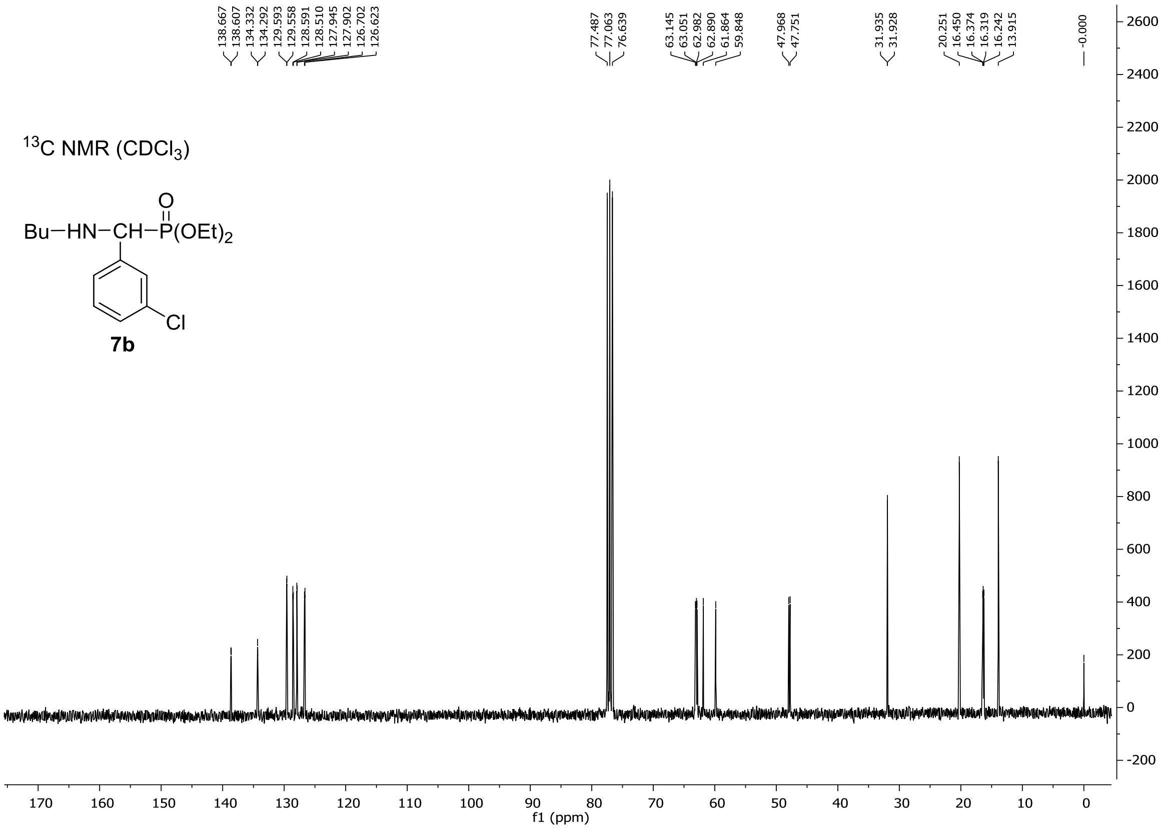
13C NMR (CDCl3)
Bu−HN−CH−P(OEt)2
O
Cl
7b
138.667
138.607
134.332
134.292
129.593
129.558
128.591
128.510
127.945
127.902
126.702
126.623
77.487
77.063
76.639
63.145
63.051
62.982
62.890
61.864
59.848
47.968
47.751
31.935
31.928
20.251
16.450
16.374
16.319
16.242
13.915
-0.000
f1 (ppm)

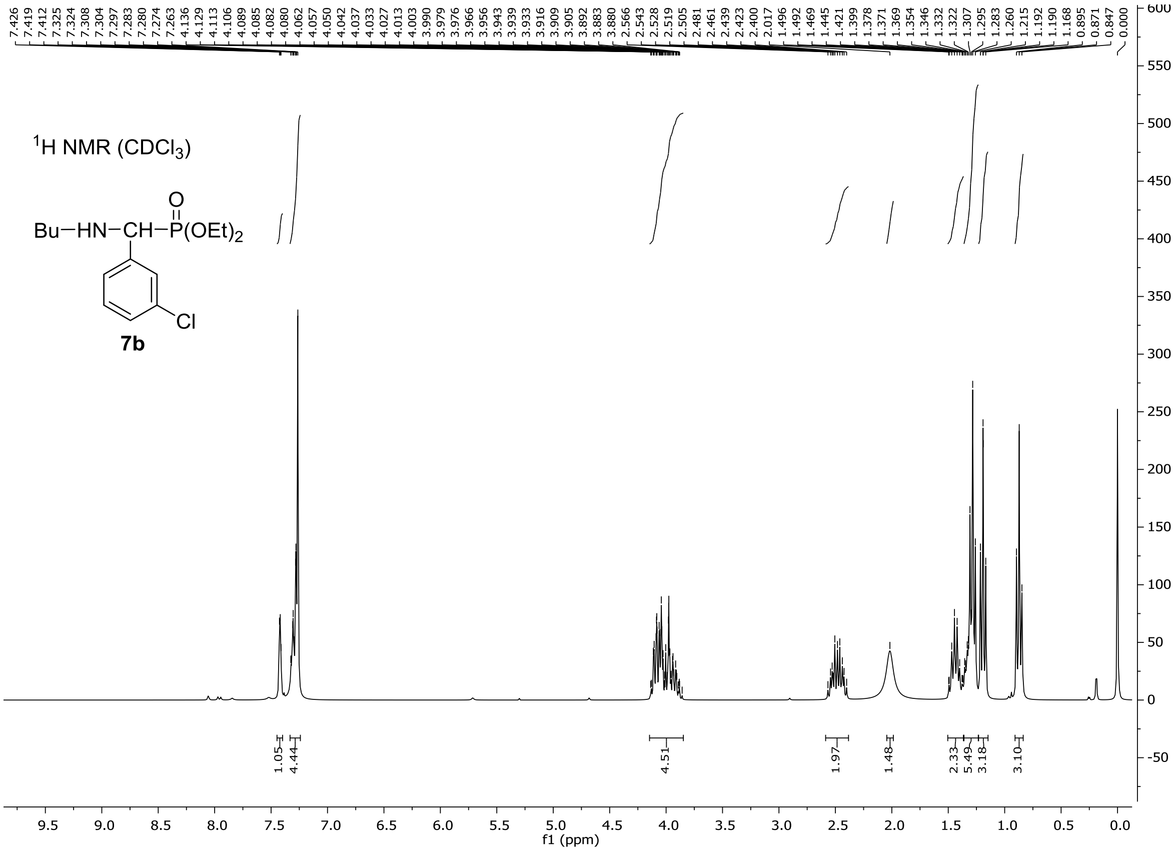

1H NMR (CDCl3)
Bu−HN−CH−P(OEt)2
O
Cl
7b
1.05
4.44
4.51
1.97
1.48
2.33
5.49
3.18
3.10
f1 (ppm)

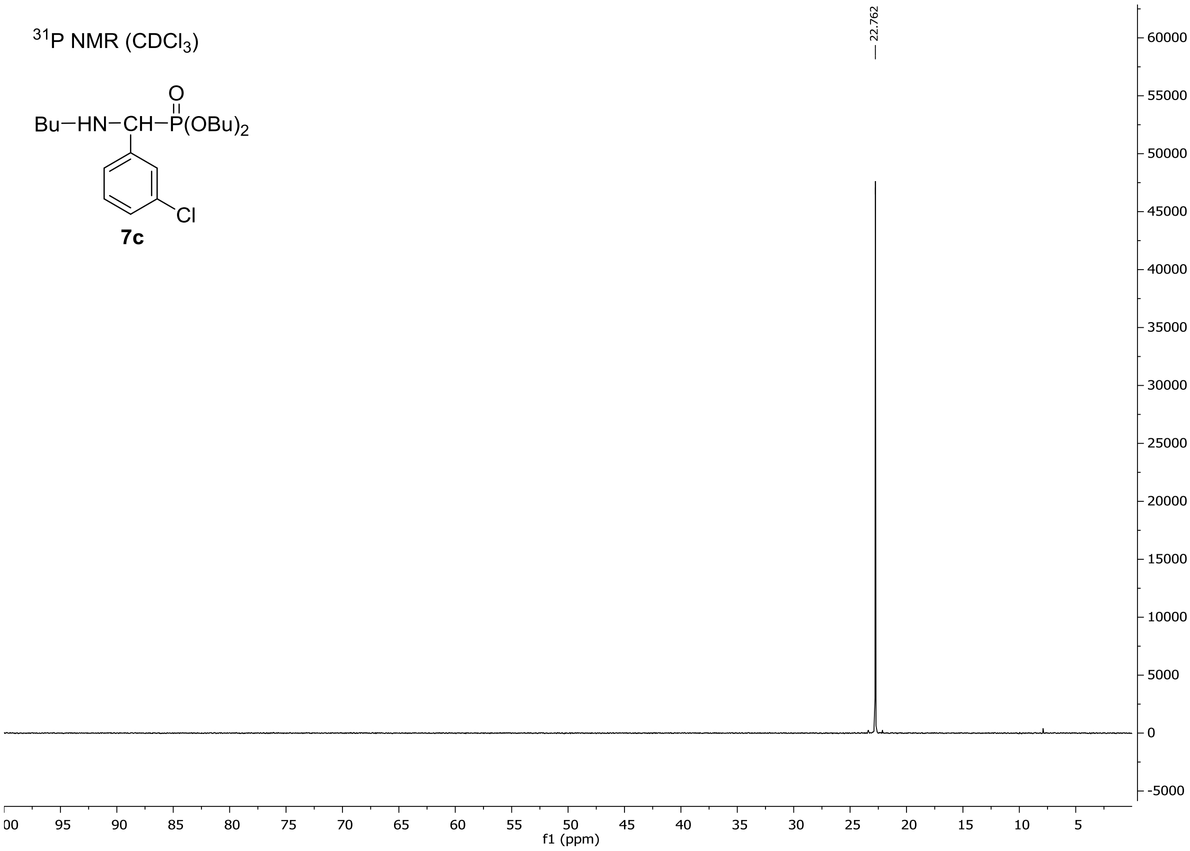

31P NMR (CDCl3)
Bu−HN−CH−P(OBu)2
O
Cl
7c
22.762
f1 (ppm)

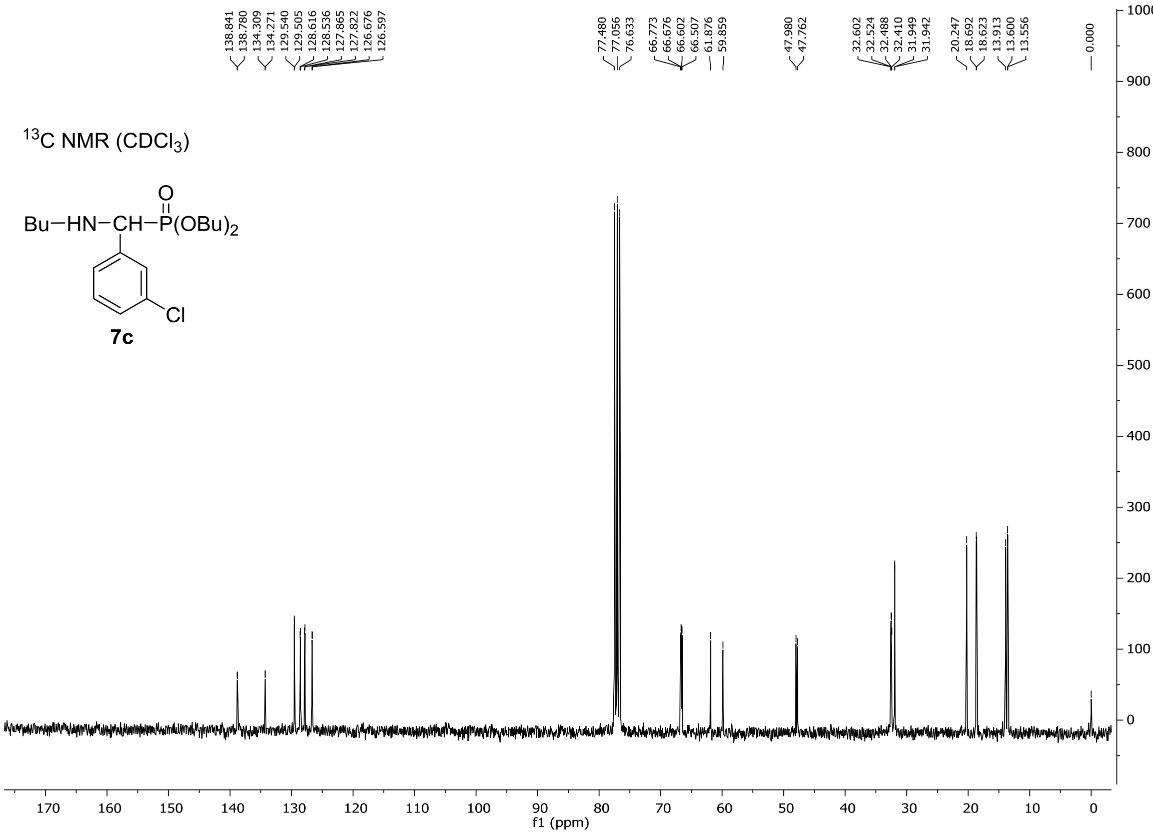
13C NMR (CDCl3)
Bu−HN−CH−P(OBu)2
O
Cl
7c
138.841 138.780 134.309 134.271 129.540 129.505 128.616 128.536 127.865 127.822 126.676 126.597
77.480 77.056 76.633 66.773 66.676 66.602 66.507 61.876 59.859
47.980 47.762
32.602 32.524 32.488 32.410 31.949 31.942 20.247 18.692 18.623 13.913 13.600 13.556
0.000
f1 (ppm)

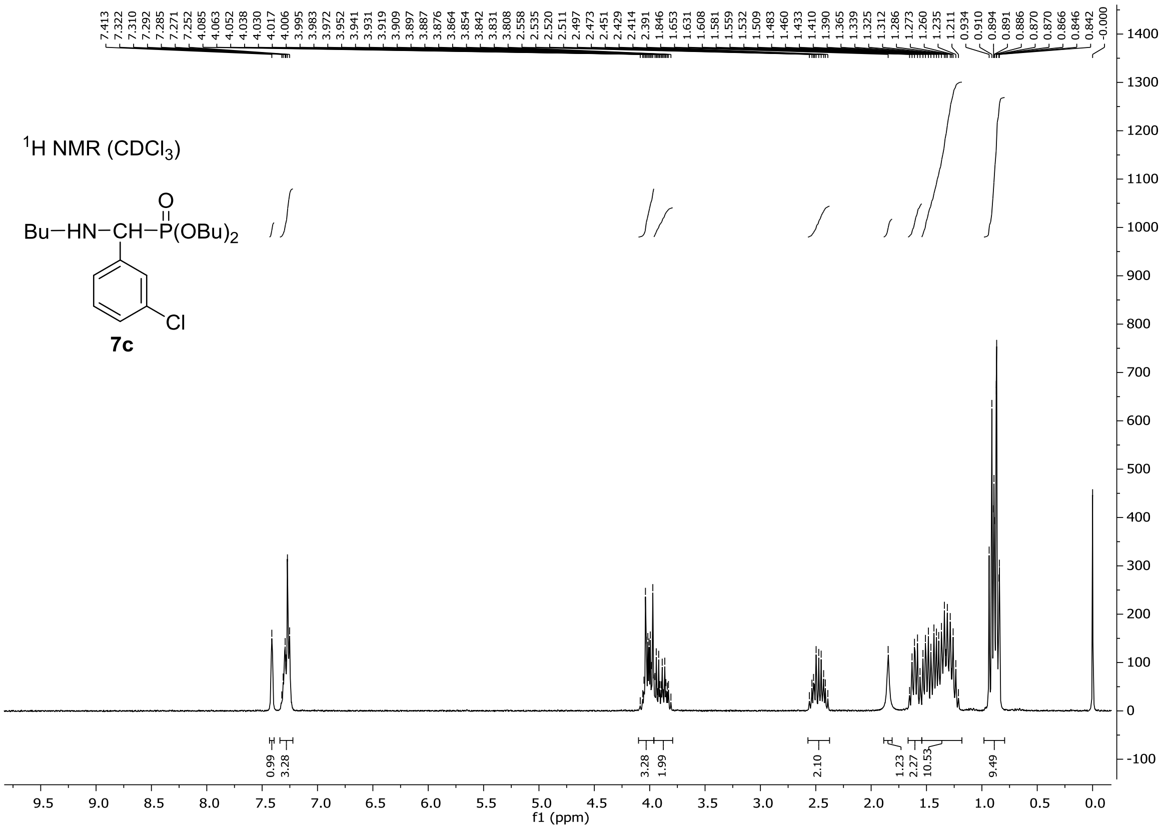
1H NMR (CDCl3)
Bu−HN−CH−P(OBu)2
O
Cl
7c
7.413 7.322 7.310 7.292 7.285 7.271 7.252 4.085 4.063 4.052 4.038 4.030 4.017 4.006 3.995 3.983 3.972 3.952 3.941 3.931 3.919 3.909 3.897 3.887 3.876 3.864 3.854 3.842 3.831 3.808 2.558 2.535 2.520 2.511 2.497 2.473 2.451 2.429 2.414 2.391 1.846 1.653 1.631 1.608 1.581 1.559 1.532 1.509 1.483 1.460 1.433 1.410 1.390 1.365 1.339 1.325 1.312 1.286 1.273 1.260 1.235 1.211 0.934 0.910 0.894 0.891 0.886 0.870 0.866 0.846 0.842 -0.000
0.99 3.28 3.28 1.99 2.10 1.23 2.27 10.53 9.49
f1 (ppm)

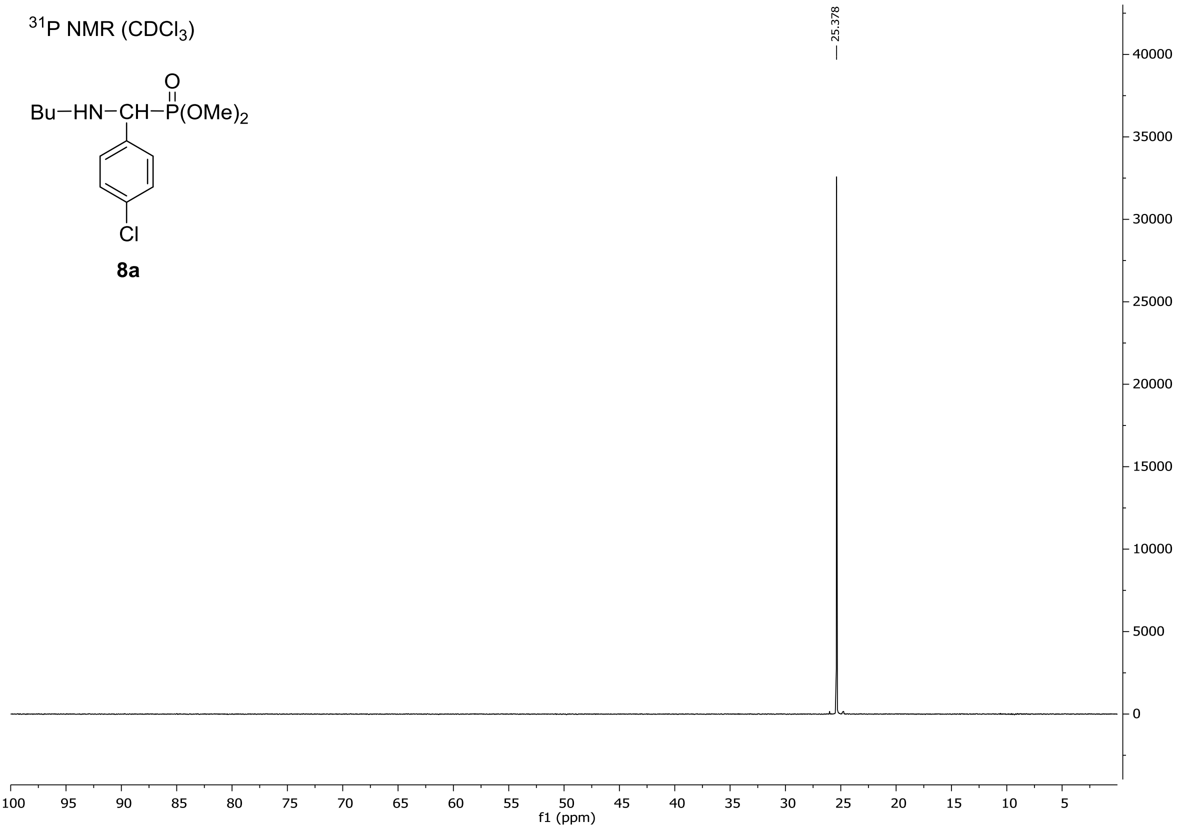
31P NMR (CDCl3)
Bu−HN−CH−P(OMe)2
O
Cl
8a
25.378
40000
35000
30000
25000
20000
15000
10000
5000
0
100 95 90 85 80 75 70 65 60 55 50 45 40 35 30 25 20 15 10 5
f1 (ppm)

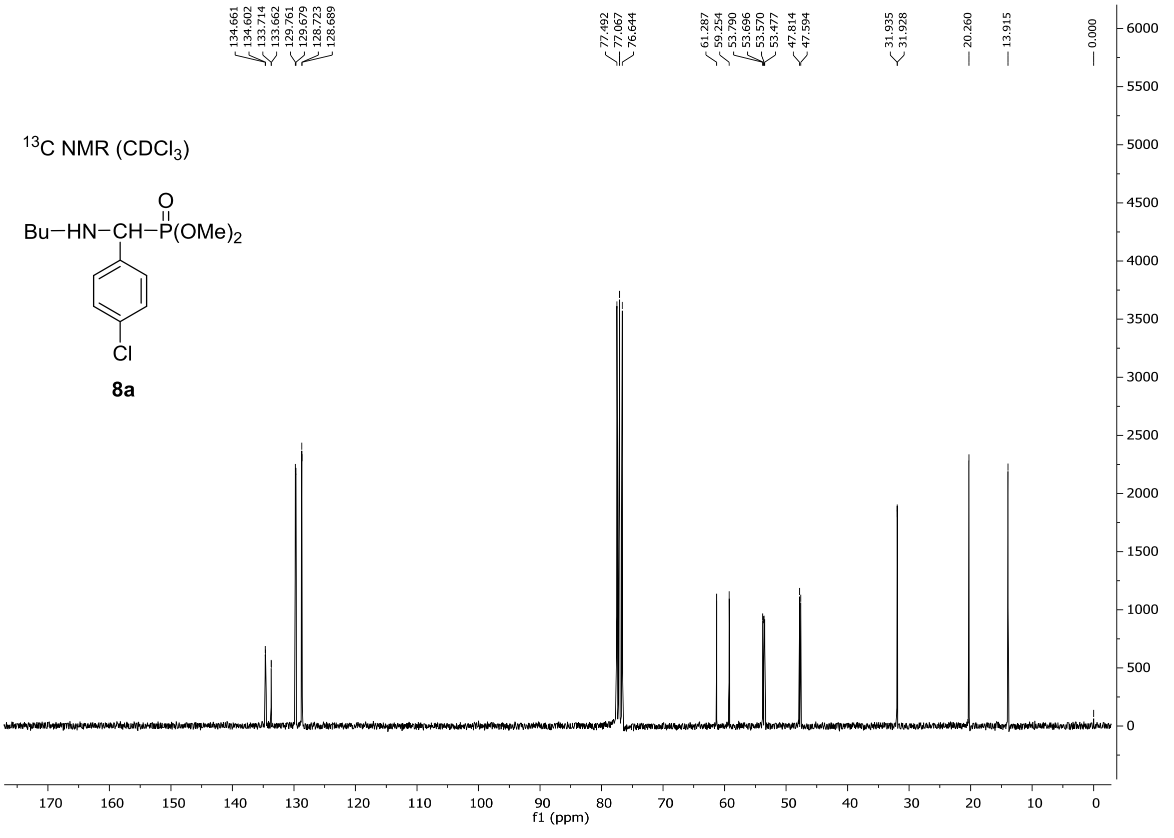
13C NMR (CDCl3)
Bu−HN−CH−P(OMe)2
O
Cl
8a
134.661
134.602
133.714
133.662
129.761
129.679
128.723
128.689
77.492
77.067
76.644
61.287
59.254
53.790
53.696
53.570
53.477
47.814
47.594
31.935
31.928
20.260
13.915
0.000
6000
5500
5000
4500
4000
3500
3000
2500
2000
1500
1000
500
0
170 160 150 140 130 120 110 100 90 80 70 60 50 40 30 20 10 0
f1 (ppm)

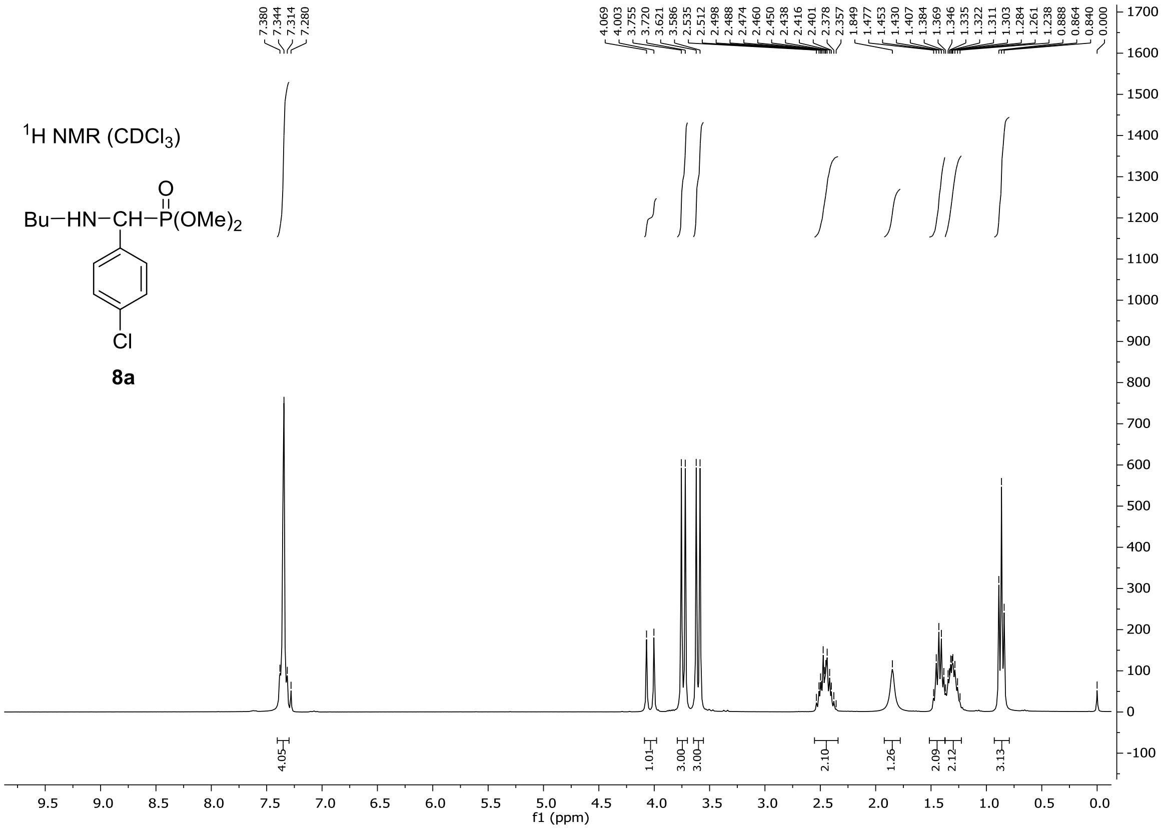

¹H NMR (CDCl₃)
Bu−HN−CH−P(OMe)₂
O
Cl
8a
7.380
7.344
7.314
7.280
4.069
4.003
3.755
3.720
3.621
3.586
2.535
2.512
2.498
2.488
2.474
2.460
2.450
2.438
2.416
2.401
2.378
2.357
1.849
1.477
1.453
1.430
1.407
1.384
1.369
1.346
1.335
1.322
1.311
1.303
1.284
1.261
1.238
0.888
0.864
0.840
0.000
4.05
1.01
3.00
3.00
2.10
1.26
2.09
2.12
3.13
f1 (ppm)

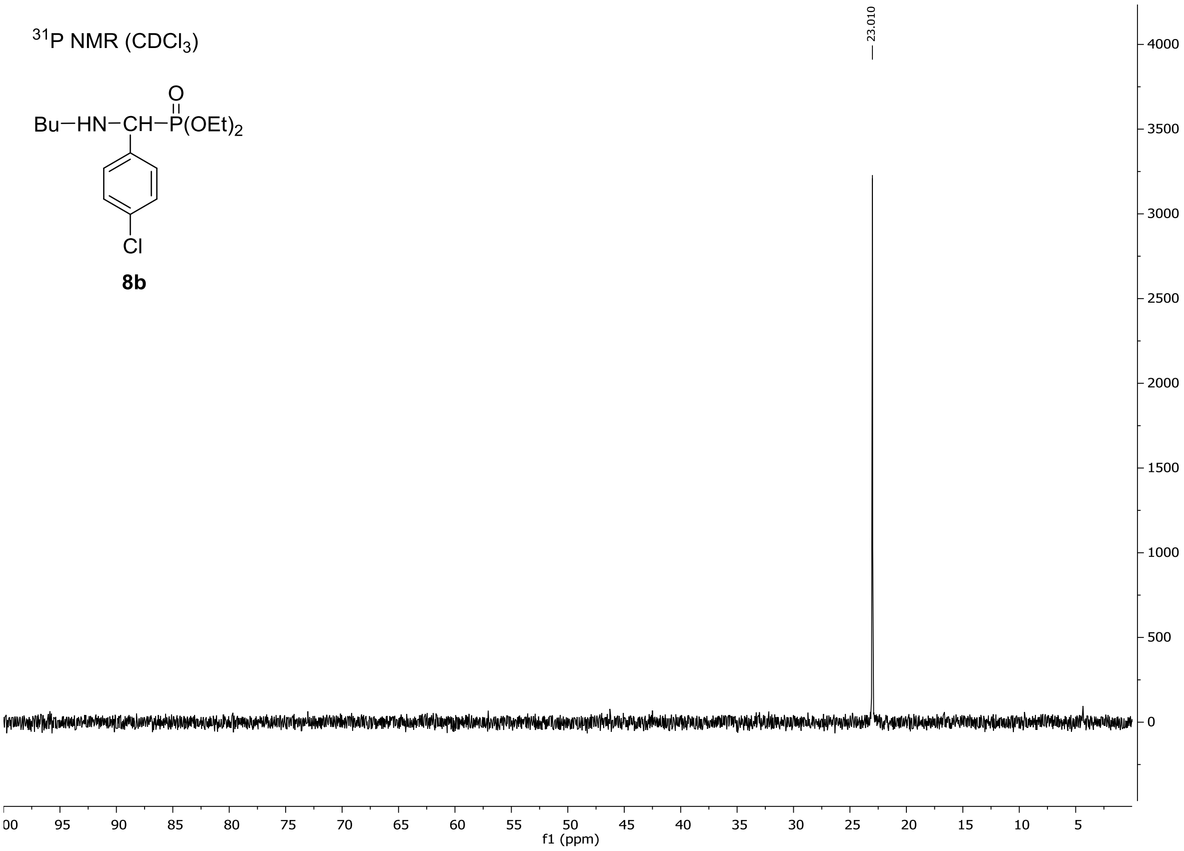

³¹P NMR (CDCl₃)
Bu−HN−CH−P(OEt)₂
O
Cl
8b
23.010
f1 (ppm)

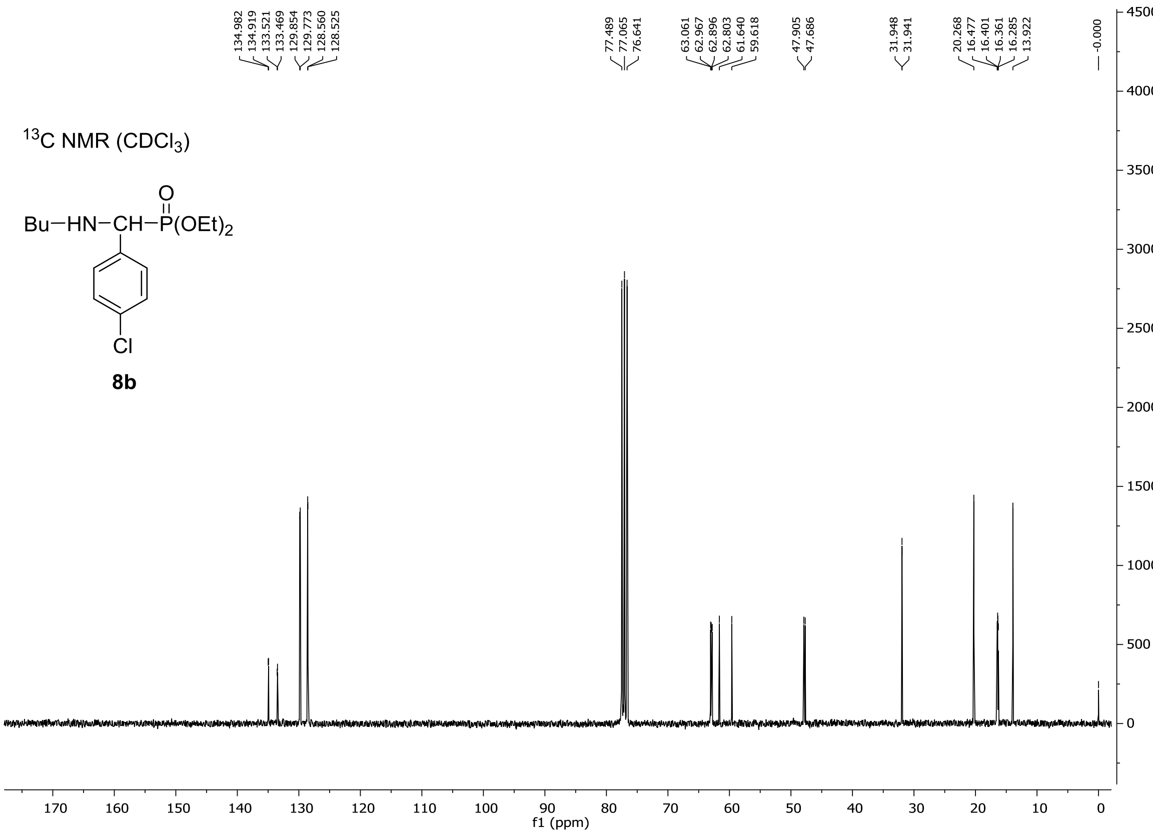
13C NMR (CDCl3)
Bu−HN−CH−P(OEt)2
O
Cl
8b
134.982 134.919 133.521 133.469 129.854 129.773 128.560 128.525
77.489 77.065 76.641
63.061 62.967 62.896 62.803 61.640 59.618
47.905 47.686
31.948 31.941
20.268 16.477 16.401 16.361 16.285 13.922
-0.000
f1 (ppm)

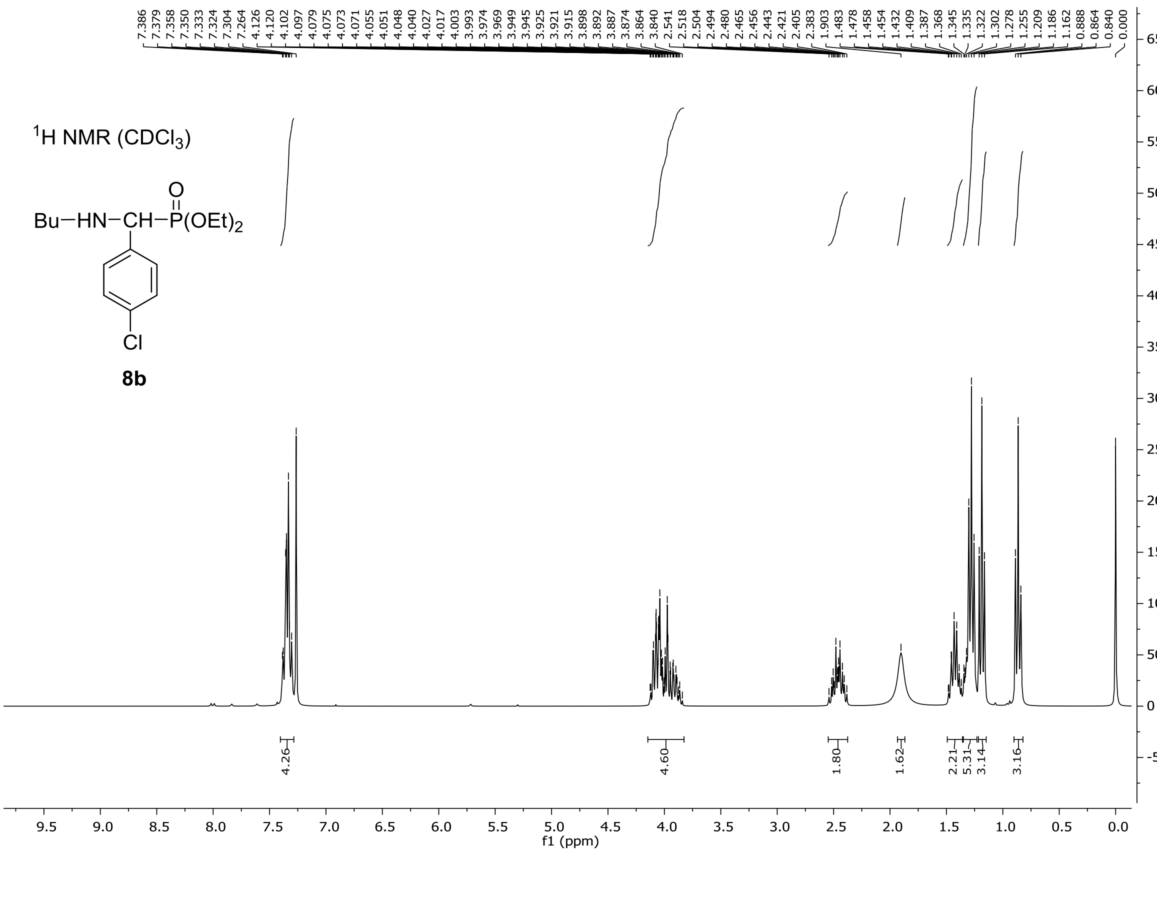
1H NMR (CDCl3)
Bu−HN−CH−P(OEt)2
O
Cl
8b
7.386 7.379 7.358 7.350 7.333 7.324 7.304 7.264 4.126 4.120 4.102 4.097 4.079 4.075 4.073 4.071 4.055 4.051 4.048 4.040 4.027 4.017 4.003 3.993 3.974 3.969 3.949 3.945 3.925 3.921 3.915 3.898 3.892 3.887 3.874 3.864 3.840 2.541 2.518 2.504 2.494 2.480 2.465 2.456 2.443 2.421 2.405 2.383 1.903 1.483 1.478 1.458 1.454 1.432 1.409 1.387 1.368 1.345 1.335 1.322 1.302 1.278 1.255 1.209 1.186 1.162 0.888 0.864 0.840 0.000
4.26
4.60
1.80
1.62
2.21
5.31
3.14
3.16
f1 (ppm)

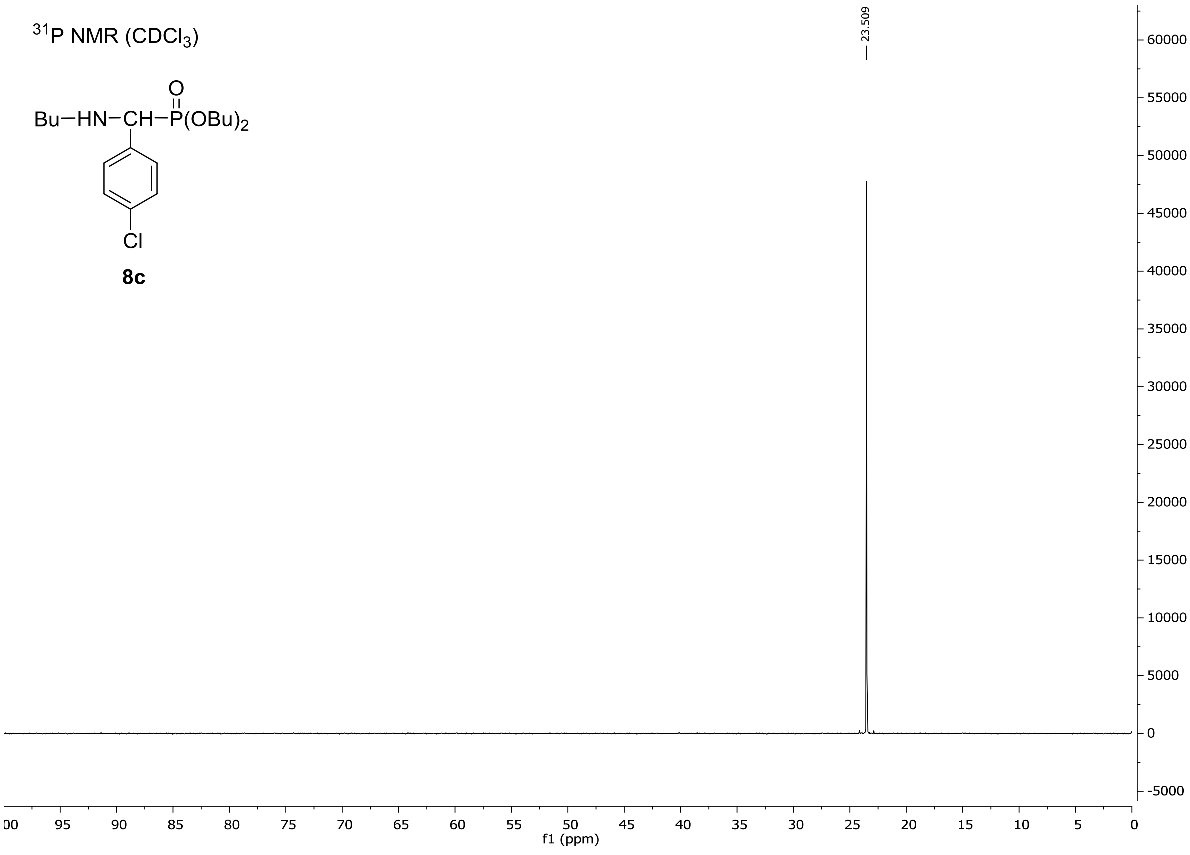
31P NMR (CDCl3)
Bu−HN−CH−P(OBu)2
O
Cl
8c
23.509
f1 (ppm)

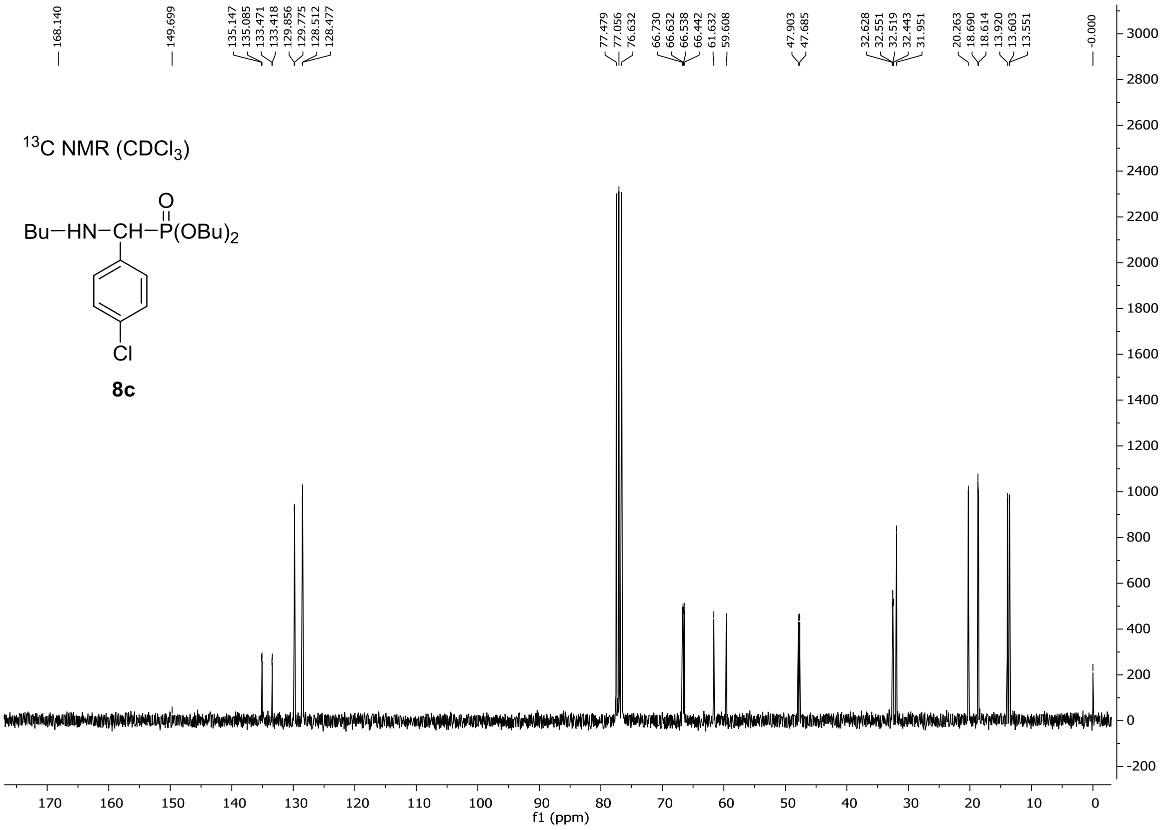
13C NMR (CDCl3)
Bu−HN−CH−P(OBu)2
O
Cl
8c
168.140
149.699
135.147
135.085
133.471
133.418
129.856
129.775
128.512
128.477
77.479
77.056
76.632
66.730
66.632
66.538
66.442
61.632
59.608
47.903
47.685
32.628
32.551
32.519
32.443
31.951
20.263
18.690
18.614
13.920
13.603
13.551
-0.000
f1 (ppm)

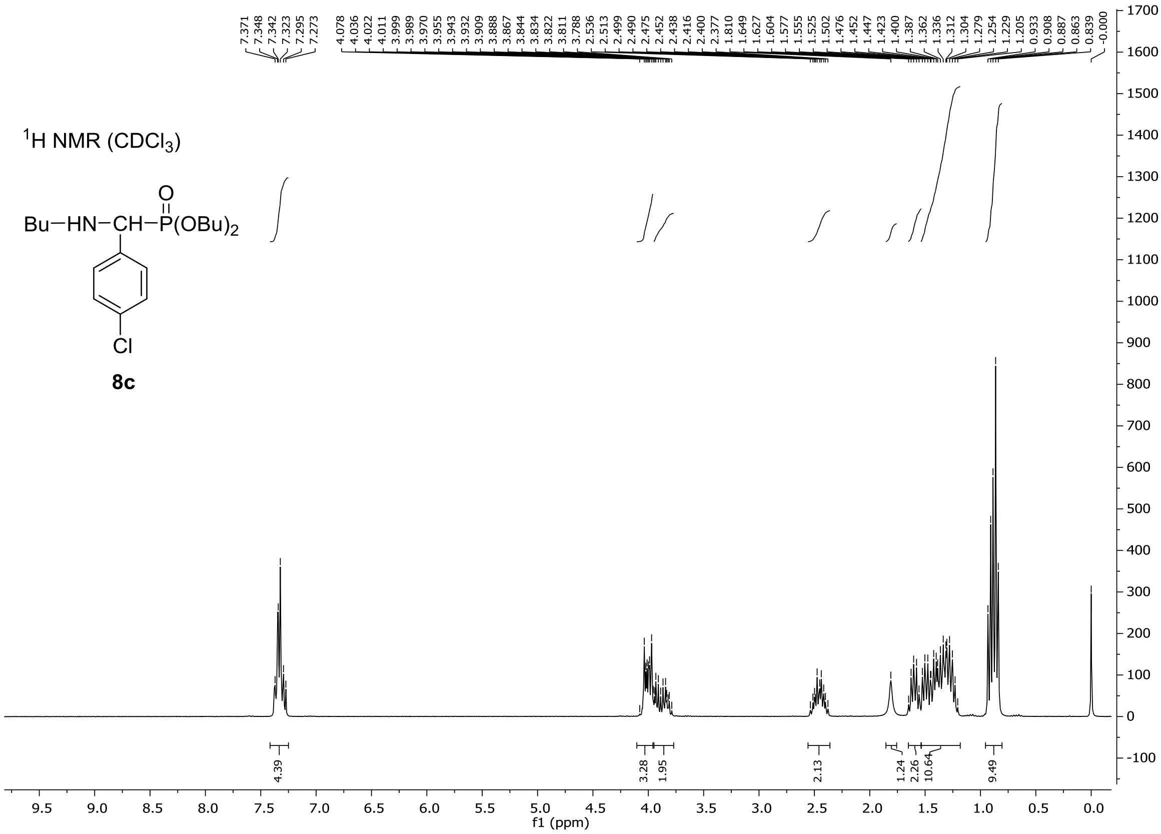
^{1}H NMR ($CDCl_3$)
Bu−HN−CH−P(OBu)$_2$
O
Cl
8c
f1 (ppm)

References

S1 Zhang, E.; Tian, H.; Xu, S.; Yu, X.; Xu, Q. *Org. Lett.* **2013**, *15*, 2704–2707.

S2 Song, Q.-W.; Yu, B.; Liu, A.-H.; He, Y.; Yang, Z.-Z.; Diao, Z.-F.; Song, Q.-C.; Li, X.-D.; He, L.-N. *RSC Adv.* **2013**, *3*, 19009–19014.

S3 Kuzmina, O. M.; Knochel, P. *Org. Lett.* **2014**, *16*, 5208–5211.

S4 Xu, J.; Zhuang, R.; Bao, L.; Tang, G.; Zhao, Y. *Green Chem.* **2012**, *14*, 2384–2387.

S5 Kiss, N. Z.; Kaszás, A.; Drahos, L.; Mucsi, Z.; Keglevich, G. *Tetrahedron Lett.* **2012**, *53*, 207–209.

S6 Keglevich, G.; Kiss, N. Z.; Menyhárd, D. K.; Fehérvári, A.; Csontos, I. *Heteroatom Chem.* **2012**, *23*, 171–178.

S7 Ghafuri, H.; Rashidizadeh, A.; Zand, H. R. E. *RSC Adv.* **2016**, *6*, 16046–16054.

S8 Li, X.-C.; Gong, S.-S.; Zeng, D.-Y.; You, Y.-H.; Sun, Q. *Tetrahedron Lett.* **2016**, *57*, 1782–1785.

S9 Milen, M.; Ábrányi-Balogh, P.; Dancsó, A.; Frigyes, D.; Pongó, L.; Keglevich, G. *Tetrahedron Lett.* **2013**, *54*, 5430–5433.

S10 Gallardo-Macias, R.; Nakayama, K. *Synthesis* **2010**, 57–62.

S11 Keglevich, G.; Szekrényi, A. *Lett. Org. Chem.* **2008**, *5*, 616–622.

S12 Tang, J.; Wang, L.; Wang, W.; Zhang, L.; Wu, S.; Mao, D. *J. Fluorine Chem.* **2011**, *132*, 102–106.

S13 Sheldrick, G. M. *Acta Cryst*. **2008**, *64*, 112–122.

S14 Spek, A. L *Acta Cryst*. **2009**, *65*, 148–155.

S15 *Diamond - Crystal and Molecular Structure Visualization*, Crystal Impact - Dr. H. Putz & Dr. K. Brandenburg, GbR.: Kreuzherrenstr. 102, 53227 Bonn, Germany.

S16 Becke, A. D. *J. Chem. Phys.* **1993**, *98*, 5648–5656.

S17 Lee, C.; Yang, W.; Parr, R. G. *Phys. Rev. B* **1988**, *37*, 785–789.

S18 Stephens, P. J.; Devlin, F. J.; Chabalowski, C. F.; Frisch, M. J. *J. Phys. Chem.* **1994**, *98*, 11623–11627.

S19 Francl, M. M.; Pietro, W. J.; Hehre, W. J.; Binkley, J. S.; DeFrees, D. J.; Pople, J. A.; Gordon, M. S. *J. Chem. Phys.* **1982**, *77*, 3654–3665.

S20 Hariharan, P. C.; Pople, J. A. *Theoret. Chim. Acta* **1973**, *28*, 213–222.

S21 *Gaussian 09*, Revision D.01; Gaussian, Inc.: Wallingford, CT, **2009**.

S22 Tomasi, J.; Mennucci, B.; Cancès, E. *J. Mol. Struct. THEOCHEM* **1999**, *464*, 211–226.

S23 Tomasi, J.; Mennucci, B.; Cammi, R. *Chem. Rev.* **2005**, *105*, 2999–3093.
